# Antitumor immune effects of preoperative sitravatinib and nivolumab in oral cavity cancer: SNOW window-of-opportunity study

**DOI:** 10.1136/jitc-2021-003476

**Published:** 2021-10-01

**Authors:** Marc Oliva, Douglas Chepeha, Daniel V Araujo, J. Javier Diaz-Mejia, Peter Olson, Amy Prawira, Anna Spreafico, Scott V Bratman, Tina Shek, John de Almeida, Aaron R Hansen, Andrew Hope, David Goldstein, Ilan Weinreb, Stephen Smith, Bayardo Perez-Ordoñez, Jonathan Irish, Dax Torti, Jeffrey P. Bruce, Ben X. Wang, Anthony Fortuna, Trevor J. Pugh, Hirak Der-Torossian, Ronald Shazer, Nickolas Attanasio, Qingyan Au, Antony Tin, Jordan Feeney, Himanshu Sethi, Alexey Aleshin, Isan Chen, Lillian Siu

**Affiliations:** 1 Department of Medical Oncology, Institut Catala d’ Oncologia, L’Hospitalet de Llobregat, Barcelona, Spain; 2 Division of Medical Oncology and Haematology, Princess Margaret Cancer Centre, University Health Network, Toronto, Ontario, Canada; 3 Department of Otolaryngology and Head and Neck Surgery, Princess Margaret Cancer Centre, University Health Network, Toronto, Ontario, Canada; 4 Division of Medical Oncology, Hospital de Base São Jose do Rio Preto, Sao Paulo, Brazil; 5 Tumor Immunotherapy Program, Princess Margaret Cancer Centre, Toronto, Ontario, Canada; 6 Department of Research, Mirati Therapeutics, San Diego, California, USA; 7 Department of Medical Oncology, The Kinghorn Cancer Centre, St Vincent’s Hospital, Sidney, New South Wales, Australia; 8 Radiation Medicine Program, Princess Margaret Hospital, University Health Network, Toronto, Ontario, Canada; 9 Department of Radiation Oncology, University of Toronto, Toronto, Ontario, Canada; 10 Department of Pathology, University Health Network, Toronto, Ontario, Canada; 11 Ontario Institute for Cancer Research, Toronto, Ontario, Canada; 12 Department of Immunology, University of Toronto, Toronto, Ontario, Canada; 13 Department of Medical Biophysics, University of Toronto, Toronto, Ontario, Canada; 14 Clinical Development, Mirati Therapeutics, San Diego, California, USA; 15 Neogenomics Laboratories, Fort Myers, Florida, USA; 16 Natera Inc, San Carlos, California, USA

**Keywords:** head and neck neoplasms, immunotherapy, macrophages, clinical trials as topic, tumor biomarkers

## Abstract

**Background:**

Sitravatinib, a tyrosine kinase inhibitor that targets TYRO3, AXL, MERTK and the VEGF receptor family, is predicted to increase the M1 to M2-polarized tumor-associated macrophages ratio in the tumor microenvironment and have synergistic antitumor activity in combination with anti-programmed death-1/ligand-1 agents. SNOW is a window-of-opportunity study designed to evaluate the immune and molecular effects of preoperative sitravatinib and nivolumab in patients with oral cavity squamous cell carcinoma.

**Methods:**

Patients with newly-diagnosed untreated T2-4a, N0-2 or T1 >1 cm-N2 oral cavity carcinomas were eligible. All patients received sitravatinib 120 mg daily from day 1 up to 48 hours pre-surgery and one dose of nivolumab 240 mg on day 15. Surgery was planned between day 23 and 30. Standard of care adjuvant radiotherapy was given based on clinical stage. Tumor photographs, fresh tumor biopsies and blood samples were collected at baseline, at day 15 after sitravatinib alone, and at surgery after sitravatinib–nivolumab combination. Tumor flow cytometry, multiplex immunofluorescence staining and single-cell RNA sequencing (scRNAseq) were performed on tumor biopsies to study changes in immune-cell populations. Tumor whole-exome sequencing and circulating tumor DNA and cell-free DNA were evaluated at each time point.

**Results:**

Ten patients were included. Grade 3 toxicity occurred in one patient (hypertension); one patient required sitravatinib dose reduction, and one patient required discontinuation and surgery delay due to G2 thrombocytopenia. Nine patients had clinical-to-pathological downstaging, with one complete response. Independent pathological treatment response (PTR) assessment confirmed a complete PTR and two major PTRs. With a median follow-up of 21 months, all patients are alive with no recurrence. Circulating tumor DNA and cell-free DNA dynamics correlated with clinical and pathological response and distinguished two patient groups with different tumor biological behavior after sitravatinib alone (1A) versus sitravatinib–nivolumab (1B). Tumor immunophenotyping and scRNAseq analyses revealed differential changes in the expression of immune cell populations and sitravatinib-targeted and hypoxia-related genes in group 1A vs 1B patients.

**Conclusions:**

The SNOW study shows sitravatinib plus nivolumab is safe and leads to deep clinical and pathological responses in oral cavity carcinomas. Multi-omic biomarker analyses dissect the differential molecular effects of sitravatinib versus the sitravatinib–nivolumab and revealed patients with distinct tumor biology behavior.

**Trial registration number:**

NCT03575598.

## Introduction

Receptor tyrosine kinases (RTKs) including VEGFR, c-KIT, MET and the TYRO3, AXL, and MERTK (TAM) family are key regulators of cell survival pathways implicated in tumor growth and invasion, metastatic progression and tumor angiogenesis.^1–3^ The activation of these oncogenic pathways also plays a role in promoting an immunosuppressive tumor microenvironment (TME) by downregulating innate immune responses via induction of M2-polarized macrophages, natural killer cell dysfunction and suppression of antigen presentation. In addition, other mechanisms of immunosuppression include an increase in infiltration by inhibitory immune cell populations, such as regulatory T cells (Treg) and myeloid-derived suppressor cells (MDSC); and by enhancing tumor hypoxia that precludes recruitment of effector T cells.^4–7^

Tumors characterized by an immunosuppressive microenvironment and a lack of T cell infiltration are less likely to respond to anti-programmed death-1/ligand-1 (anti-PD-1/PD-L1) therapy, therefore representing a mechanism of primary resistance to these agents.^8^ As such, several RTK inhibitors with antiangiogenic properties are now being tested in combination with anti-PD-1/PD-L1 treatment in multiple cancer types with the aim of restoring effective antitumor immune responses, with a few achieving regulatory approval status.^9–12^ Sitravatinib is an orally available RTK inhibitor targeting the TAM family of receptors as well as VEGFR2, c-KIT and MET that is predicted to modulate the TME towards a less immunosuppressive state. Sitravatinib has demonstrated potent, concentration-dependent inhibition of these targets both in vitro and in vivo and has shown synergistic antitumor immune effects when combined with anti-PD-1 agents in syngeneic mouse models.^13^ Preliminary results from early phase 1 studies evaluating sitravatinib plus nivolumab showed the combination was well tolerated, as such it is now being explored in several advanced tumor types (NCT04727996 and NCT03906071).

Anti-PD-1 agents have shown durable responses and improved survival in patients with recurrent or metastatic head and neck squamous cell carcinoma (HNSCC), including oral cavity primaries, and are now being evaluated in the locoregionally advanced setting (ie, NCT03040999 and NCT02999087^14–16^). Neoadjuvant anti-PD-1 therapy in patients with resectable HNSCC has been investigated in small studies showing promising antitumor activity with an overall safe toxicity profile, leading to ongoing large randomized trials (ie, NCT03765918).^17 18^ However, about half of these patients did not respond to neoadjuvant anti-PD-1 therapy, thus, combination strategies using other immuno-oncology agents or targeted therapies such as antiangiogenic agents are under evaluation (NCT04199104 and NCT04675294).^19 20^ MET, AXL and VEGF overexpression is associated with early nodal and distant metastasis as well as with mechanisms of resistance to radiation and systemic therapies in HNSCC.^21–23^ The immune contexture within the TME of HNSCC has shown to be prognostic and also predictive of response to anti-PD-1/PD-L1 agents.^24^ Patients presenting with resectable oral cavity squamous cell carcinoma (OCSCC) represent a unique population whose primary tumors are relatively accessible to biopsies, and short treatment and assessment windows will not compromise curative intent, standard of care therapies.^25 26^ Clinical trials evaluating investigational agents in this patient population offer a ‘window-of-opportunity’ (WOO) to examine molecular endpoints and pharmacodynamic effects of novel drugs or drug combinations. SNOW is a biomarker-driven WOO study designed to evaluate the immunologic and molecular effects of preoperative sitravatinib and nivolumab in patients with resectable OCSCC.

## Results

### Preoperative treatment with sitravatinib and nivolumab was safe and led to deep pathological responses

A total of 10 patients were treated in the SNOW study between August 2018 and May 2020, median follow-up was 21 months (range 14–27 months) with data cut-off occurring on December 31, 2020. Cohort characteristics, clinical and pathological staging, treatment received and pathological treatment response (PTR) are summarized in table 1. Most patients were men, active or former smokers and presented with locoregionally advanced disease (stage III–IVA). Nine patients completed study treatment and had surgery within the planned window, while patient *S-009* discontinued sitravatinib on day 15 due to transient grade 2 related thrombocytopenia, which led to a 2-week delay of the initial surgery date. Of note, this patient did receive nivolumab on day 15. All patients but *S-008* (cT2N0) underwent postoperative radiotherapy (total dose 60–66 Gy) based on their clinical stage at baseline. None of the patients received adjuvant chemotherapy as none had positive margins or extranodal extension in their pathological specimens.

**Table 1:** Patient characteristics

Patient ID	Age (years)	Gender	Smoking status (pack-year)	HPV status	PD-L1 CPS	PD-L1 TPS	Primary location	Clinical TNM	Sitravatinib (days)	Nivolumab dosed	Surgery within window	Pathologic TNM	PTR (% of viable tumor)	Margins (>5 mm)	ENE	PORT
S-001	58	Male	Never	Negative	>20	>50	Alveolus	cT4aN2b	21	Yes	Yes	ypT0N0	cPR (0)	Negative	No	Yes
S-002	58	Female	Never	Negative	>20	>50	Alveolus	cT4aN2b	21	Yes	Yes	ypT4aN0*	mPR (<10)	Negative	No	Yes
S-004	59	Male	Current (30)	Negative	>20	1–50	Tongue	cT3N1	28	Yes	Yes	ypT2N0	iPR (65)	Negative	No	Yes
S-006	59	Male	Former (40)	Negative	>20	1–50	Tongue	cT3N1	21	Yes	Yes	ypT3N1	iPR (36)	Negative	No	Yes
S-007	63	Male	Current (50)	Negative	<1	<1	Floor of mouth	cT4aN2c	21	Yes	Yes	ypT4aN0	iPR (94)	Negative	No	Yes
S-008	60	Male	Former (30)	Negative	1–20	1–50	Tongue	cT2N0	21	Yes	Yes	ypT1pN0	iPR (100)†	Negative	No	No
S-009	71	Female	Never	Negative	>20	1–50	Alveolus	cT4aN1	15	Yes	No	ypT3pN0	iPR (95)	Negative	No	Yes
S-010	68	Male	Former (20)	Positive	1–20	1–50	Tongue	cT2N2b	28	Yes	Yes	ypT1pN2a	iPR (38)	Negative	No	Yes
S-011	50	Male	Current (40)	Negative	1–20	<1	Tongue	cT3N0	28	Yes	Yes	ypT1pN1	iPR (87)	Negative	No	Yes
S-013	53	Male	Never	Negative	>20	1–50	Alveolus	cT4aN0	28	Yes	Yes	ypT2pN0‡	mPR (<10)	Negative	No	Yes

*No invasive squamous cell carcinoma found in the mucosa, only remaining focis in the bone.

†iPR (100): this patient’s surgical specimen had residual viable tumor in all the areas evaluated.

‡Prominent lymphohistiocytic inflammatory infiltrate with multiple giant cells and cholesterol cleft, which involve the soft tissue underneath the mucosa extensively. Remaining 1 cm tumor is also heavily ulcerated with prominent inflammatory infiltrate.

CPS, combined positive score; ENE, extranodal extension; HPV, human papillomavirus; ID, identification; PD-L1, programmed ligand-1; PORT, postoperative radiotherapy; PTR, pathological treatment response; TNM, tumor-node-metastasis classification; TPS, tumor positive score.

Treatment with sitravatinib and nivolumab was safe: pre-surgery grade 3 treatment-related adverse events (TRAEs) occurred only in one patient (sitravatinib-related asymptomatic hypertension) with no grade 4 TRAEs observed. At least one grade 1–2 TRAE occurred in all patients (online supplemental table 1). Among the total number of TRAEs reported, the most common sitravatinib-related AEs were gastrointestinal disorders (26%), dysphonia (16%) and alanine transaminase (ALT)/asparte transaminase (AST) increase (13%); whereas the most common sitravatinib and nivolumab-related AEs were fatigue (27%) and anorexia (27%) (table 2). Besides *S-009* who discontinued sitravatinib on day 15, patient *S-013* required one level dose reduction (to sitravatinib 80 mg) due to grade 1 sitravatinib-related thrombocytopenia. None of the patients had treatment-related intraoperative complications. According to Clavien-Dindo classification,^27^ two patients had grade 3a postoperative complications, but only one was deemed related to study drugs: patient *S-004* had wound infection and tracheostomy bleeding requiring intravenous antibiotics and blood transfusion, but recovered without sequelae. No other treatment-related postoperative complications occurred.

10.1136/jitc-2021-003476.supp1 Supplementary data

**Table 2:** Treatment-related adverse events (TRAEs) by type

TRAEs	Sitravatinib and nivolumab-related		Sitravatinib-related		Nivolumab-related	
	Grade 1–2 n (%)	Grade 3–4	Grade 1–2	Grade 3	Grade 1–2	Grade 3
		N (%)	N (%)	N (%)	N (%)	N (%)
Total number of events	11 (100)		39 (100)		9 (100)	
Total number of events by grade	9 (81)	2 (19)	38 (97)	1 (3)	9 (100)	0
Fatigue	3 (27)	–	3 (8)	–	2 (23)	–
Gastrointestinal disorders			10 (26)			
Nausea/vomiting	–	–	2 (5)	–	1 (11)	–
Diarrhea	–	–	4 (11)	–	–	–
Anorexia	3 (27)	–	2 (5)		–	–
Other	–	–	2 (5)	–	1 (11)	–
Arthralgias/myalgias	–	–	1 (3)	–	1 (11)	–
Skin disorders						
Rash, dryness, pruritus	1 (9)	–	2 (5)	–	2 (22)	–
Palmar–plantar erythrodysesthesia	–	–	1 (3)	–	–	–
Dysphonia	–	–	6 (16)	–	1 (11)	
Mucositis	–		3 (8)	–	1 (11)	
Hypertension	–	–	4 (11)	1 (3)	–	–
Laboratory toxicity						
ALT/AST increase	1 (9)	–	5 (13)	–	–	–
Thrombocytopenia	–	–	2 (5)	–	–	–
Proteinuria	–	–	1 (3)	–	–	–
Lipase increase	1 (9)	–	–			
Other						
Wound infection	–	1 (9)	–	–	–	–
Tracheostomy bleeding	–	1 (9)	–	–	–	–

In this table, the denominators are based on the total number of TRAEs observed for each of three categories: sitravatinib–nivolumab related; sitravatinib-related; and nivolumab-related.

ALT, alanine transaminase; AST, asparte transaminase (AST).

Nine out of 10 patients had pathological downstaging, with one complete pathological response (*S-001*). Independent PTR assessment confirmed a complete PTR (cPTR) in patient *S-001,* and identified two patients with major PTR (mPTR), with the rest being incomplete PTR (iPTR). Of note, pathological responses occurred in both PD-L1 positive and negative tumors. 5-Deoxy-5-[18F]fluoro-arabinofuranosyl-2-nitroimidazole ([18F]FAZA) positron emission tomography (PET) performed in patients *S-001* and *S-002* showed reduction in tumor hypoxia after treatment with sitravatinib–nivolumab when compared with baseline (online supplemental figure 1). At the time of data cut-off, all patients were alive with no disease recurrence.

### Circulating tumor DNA and cell-free DNA dynamics identified differential tumor biological behavior following sitravatinib alone vs sitravatinib plus nivolumab

Whole-exome sequencing (WES) was performed on all per-protocol, mandatory, fresh frozen tumor biopsies obtained at baseline, day 15 and pre-surgery for each patient. The sample with higher tumor sequencing coverage from each patient was used to select a total of 16 clonal somatic mutations to design a personalized circulating tumor DNA (ctDNA) Signatera assay. Baseline patient-specific ctDNA was detected in 7 out of 10 patients (online supplemental figure 2): among these, the median number of detectable mutations was 16 (range: 5–16), the median variant-allele frequency (VAF) was 0.113% (range: 0.011%–6.37%) and median ctDNA levels measured in mean tumor molecules (MTM) per mL of plasma was 2.1 MTM/mL (range: 0.1–252.1). Baseline cell-free DNA (cfDNA) was quantified in all patients, and the median amount was 6.15 ng/mL (range: 4.8–28.7).

**Figure 1:**
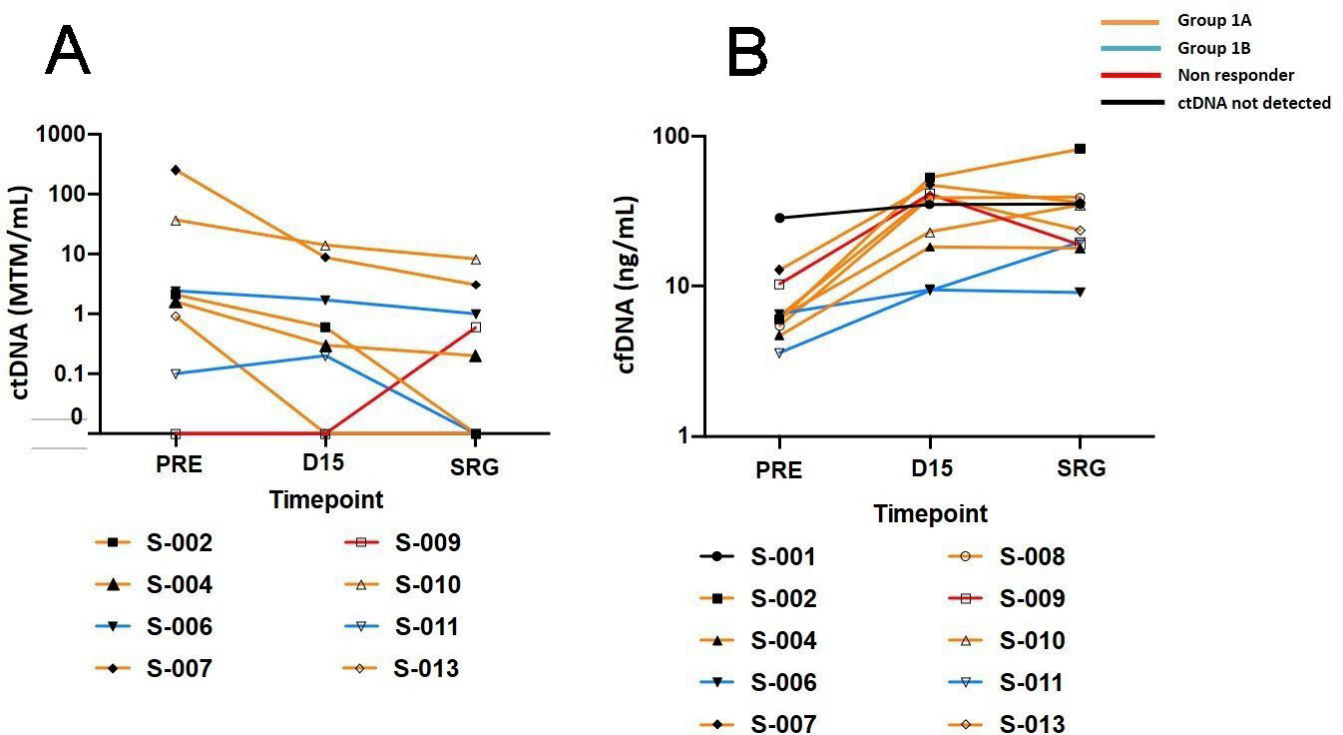
ctDNA (MTM/mL) (A) and cfDNA (ng/mL) (B) dynamics following study treatment. (A) Two-dimensional line charts showing MTM/mL at each of the three time points. (B) Two-dimensional line charts showing cfDNA in ng/mL at each of the three time points. The symbols represent each individual patient. The colors represent the groups according to the distinct tumor biological behavior following treatment. cfDNA, cell-free DNA; ctDNA, circulating tumor DNA; D15, day 15; MTM, mean tumor molecules; PRE, pretreatment; SRG, pre-surgery.

An overall reduction in ctDNA levels was observed after study treatment in all patients with detectable ctDNA at baseline (figure 1A). Median ctDNA concentration at day 15 and pre-surgery were 0.6 MTM/mL (range 0–14) and 0.6 MTM/mL (range 0–8.3), respectively, versus 2.1 MTM/mL at baseline. Patients *S-002* and *S-013*, both with mPTR, achieved ctDNA clearance before surgery (table 3). CfDNA concentration were increased following sitravatinib treatment in the whole cohort (figure 1B), with a median cfDNAd15 concentration of 36.1 ng/mL (range: 9.2–44.2) and a median fold-increase of 3.85 (range: 1.2–9) when compared with baseline (∆cfDNAd15).

**Table 3:** Tumor reduction and ctDNA/cfDNA dynamics at D15 and at SRG revealed differential tumor biological behavior

Patient ID	Tumor decreased D15	Tumor decreased pre-surgery	ctDNA detectable PRE	∆ctDNAd15	∆ctDNASRG (%)	∆cfDNAd15 (fold change)	Group classification*
S-001	Yes	Yes	No	–	–	×1.2	N/A
S-002	Yes	Ø	Yes	−71%	−100 (cleared)	×9	1A
S-004	Yes	Yes	Yes	−81%	−87	×4	1A
S-006	No	Yes	Yes	−29%	−71	×1.5	1B
S-007	Yes	Yes	Yes	−96%	−99	×3.5	1A
S-008	Yes	No	No	–	–	×7	1A
S-009	Ø	No (increased)	No	–	Detectable (+100)	×4	PD†
S-010	Yes	Yes	Yes	−62%	−77	×3.7	1A
S-011	No	Ø	Yes	+100%	−100 (cleared)	×2.5	1B
S-013	Ø	Ø	Yes	−100% (cleared)	Undetectable	×6.4	1A

*Cohort groups according to tumor biological behavior: Patients were classified into Group 1A if had at least two of the following criteria after treatment with sitravatinib alone and before nivolumab dosing (D15) = (1) ∆ctDNAd15>(−50%); (2) ∆cfDNAd15>3.8 fold change; (3) Tumor decrease at D15. Patients were classified into Group 1B if criteria 1 and 3 were not met at day 15 but were met pre-surgery (after nivolumab dosing). Patients S-001 and S-009 were excluded as they were not fitting any of these criteria.

†Patient S-009 was considered PD as per the criteria defined above (patient had tumor regrowth and spike in ctDNA before surgery) although patient had responded while on sitravatinib and had clinical to pathology downstaging from T4aN1 to pT3pN0.

Ø, not assessable; cfDNA, cell-free DNA; ctDNA, circulating tumor DNA; D15, day 15; ID, identification; N/A, not applicable; PD, patient with progressive disease; PRE, pretreatment; SRG, pre-surgery.

**Figure 2:**
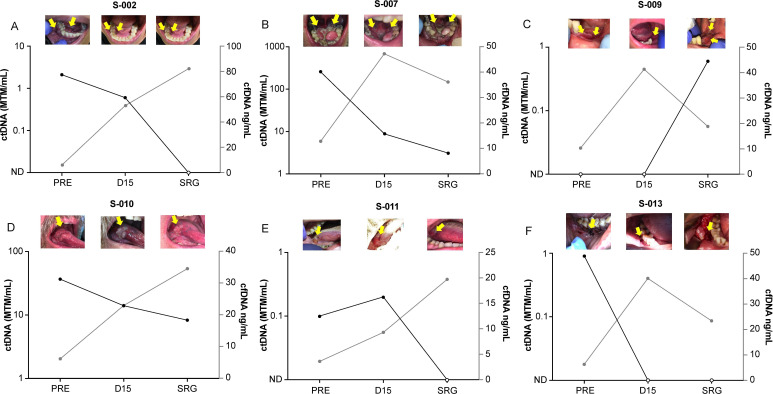
ctDNA dynamics correlated with tumor changes following sitravatinib and sitravatinib plus nivolumab. Charts showing log-scale changes in ctDNA and cfDNA (Y-axis) at each time point (X-axis) in each individual patient. Tumor photographs performed during study at each of the corresponding time points are shown above the line charts for each patient. Arrows indicate the location of the primary tumor. ctDNA, circulating tumor DNA; cfDNA, cell-free DNA; D15, day 15; MTM, mean tumor molecules; PRE, pretreatment; SRG, pre-surgery.

Dynamic changes in ctDNA and cfDNA levels at day 15 (∆ctDNAd15 and ∆cfDNAd15, respectively) and pre-surgery (∆ctDNASRG and ∆cfDNASRG, respectively), compared with baseline, correlated with the differential patterns of tumor biological behavior observed after sitravatinib alone and after sitravatinib plus nivolumab (table 3, figure 2). We observed a reduction in ∆ctDNAd15 >50% and an increase in ∆cfDNAd15 >3.85 fold in the majority of patients who had tumor reduction per investigator’s assessment following sitravatinib and prior to nivolumab. In patients *S-006* and *S-011*, who had no evident tumor reduction after sitravatinib alone, minimum or no change was observed in ∆ctDNAd15 and ∆cfDNAd15, while ∆ctDNASRG dropped following nivolumab dosing. We grouped patients according to their different tumor biological behavior following sitravatinib alone (Group 1A) and sitravatinib plus nivolumab (Group 1B) for further biomarker analyses. Patients *S-001 and S-009* were excluded from both groups: *S-001* had no detectable ctDNA at any time point and no change in cfDNA levels and thus was not included in this subanalysis; while *S-009* was classified as a progressor given the spike in the ctDNA level and tumor growth before surgery.

### Sitravatinib increased the M1 to M2-type macrophages ratio at D15 in Group 1A

In vitro studies using lung cancer cell lines have demonstrated that sitravatinib suppresses the expression of markers associated with immunosuppressive phenotype macrophages via MERTK inhibition, preventing polarization into M2-type macrophages.^13^ In addition, in vivo studies using immune-competent mice have shown that sitravatinib leads to a reduction in intratumoral M2 macrophages and monocytic MDSCs, an increase in CD8+ T cells and a higher expression of pro-inflammatory genes, including PD-L1.^13^

**Figure 3:**
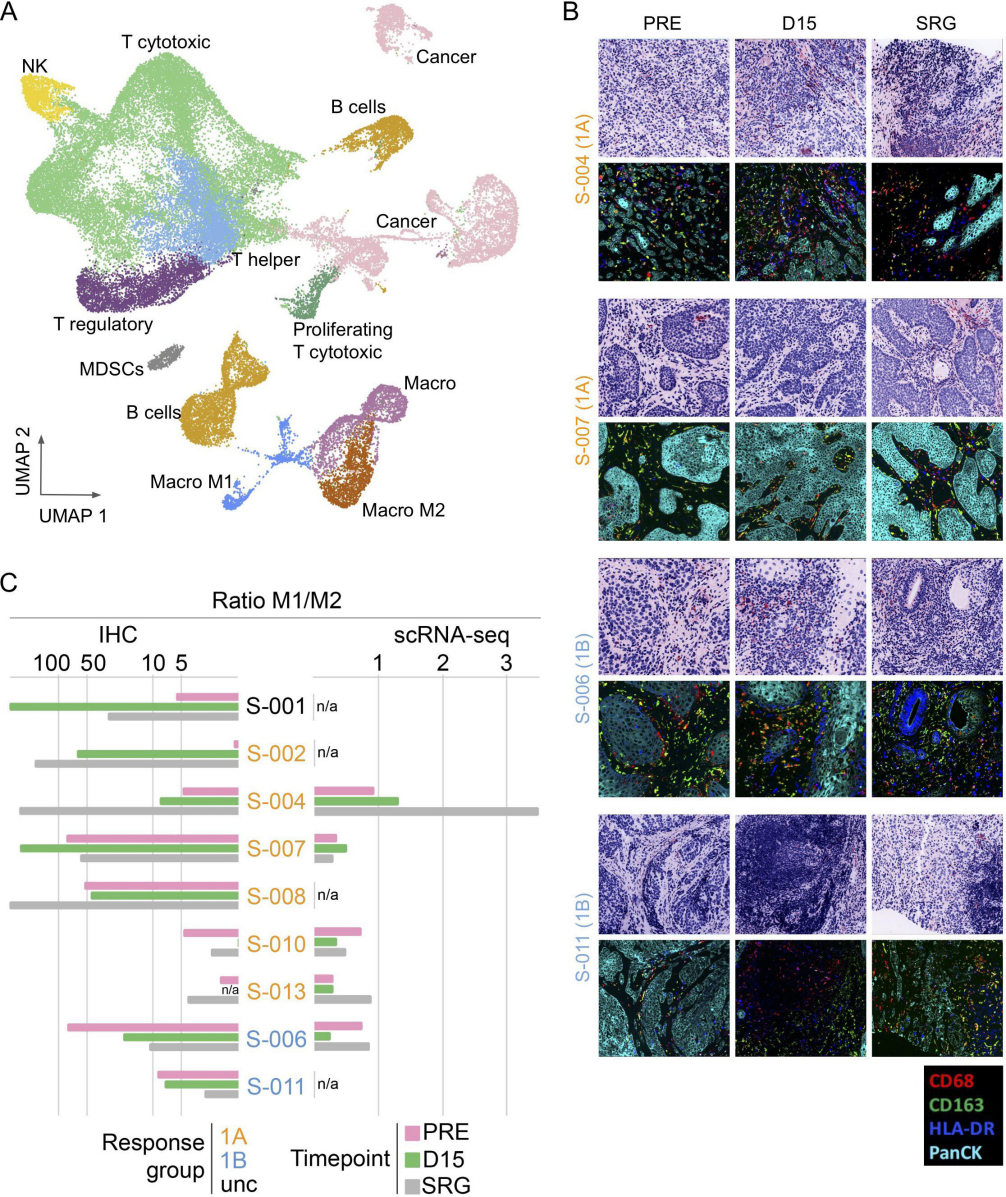
Changes in tumor-associated macrophage populations following sitravatinib alone (D15) and sitravatinib plus nivolumab (SRG). (A) Uniform Manifold Approximation and Projection (UMAP) plots showing the integration of 15 samples from five SNOW patients at three time points. Colors represent cell types. (B) Multiplexing immuno-fluorescence staining in tumor biopsies at pre-treatment (PRE), day 15 (D15) and pre-surgery (SRG) using NeoGenomics MultiOmyx panels showing changes in macrophages subpopulations: M1 type (CD68+CD163–) shown in red, M2 type (CD68+CD163+) shown in yellow and M1 intermediate type (CD68+HLA-DR+CD163–) shown in magenta. Upper images show H&E staining of tissue sample. (C) Ratio of M1/M2 macrophages detected using IHC and scRNAseq measurements. Orange: 1A=responders to sitravatinib; blue: 1B=responders to sitravatinib–nivolumab; black=unclassifiable. IHC, immunohistochemistry; NK, natural killer; MDSC, myeloid-derived suppressor cell; scRNAseq, single-cell RNA sequencing.

In the SNOW study, changes in intratumoral myeloid and lymphoid-derived immune cell populations were evaluated using multiplexed immunohistochemistry (IHC), tumor flow cytometry and single-cell RNA sequencing (scRNAseq) analyses (see online supplemental figure 3 for available samples for each patient and biomarker). An overall reduction of intratumoral M2-type macrophages (CD68+CD163+) and an increase in the M1 (CD68+CD163–) to M2 ratio were observed at day 15 in the majority of patients in Group 1A but in none of those in Group 1B, using both multiplexed IHC and scRNAseq techniques (figure 3). These effects were deeper in patients who had cPTR or mPTR such as *S-001 and S-00*2. Patient-to-patient comparisons by technique used (multiplexed IHC vs scRNAseq) showed overall similar findings, with a few exceptions (figure 3C). For instance, patient *S-010* had low cellularity in the multiplexed IHC analysis and macrophage changes were not consistent with findings of other patients in Group 1A: both IHC and scRNAseq analysis showed a decrease in M1 to M2 ratio in this patient at day 15.

Although macrophage subpopulations could not be distinguished using flow cytometry on tumor cells, we observed an overall decrease in the frequency of tumor-associated macrophages and monocytes at day 15 versus baseline in patients from Group 1A but not in Group 1B patient *S-006* (online supplemental figure 4). We observed a lower proportion of tumor-associated macrophages expressing the inhibitory molecules PD-L1+, PD-L2+ and B7-H4+ at surgery in comparison to baseline in patients from Group 1A. In Group 1B patient *S-006,* the proportion of PD-L1+ and PD-L2+ tumor-associated macrophages were lower at day 15 in comparison to baseline, but highest at surgery.

Additional potentially anticipated changes in other immune cell subsets following sitravatinib such as reduction of Tregs (CD3+CD4+CD127–FOXP3+) as well as increase in activated CD8+ T cells (CD3 +CD8+PD-1+) were evaluated (online supplemental figures 4,5): multiplex IHC analysis showed a decrease in Tregs at day 15 and surgery time points from major responders to sitravatinib, *S-001* and *S-002,* while there was an increase at day 15 in patients from Group 1B, *S-006* and *S-011*. Other changes were inconsistent across the cohort and no conclusions could be drawn.

**Figure 4:**
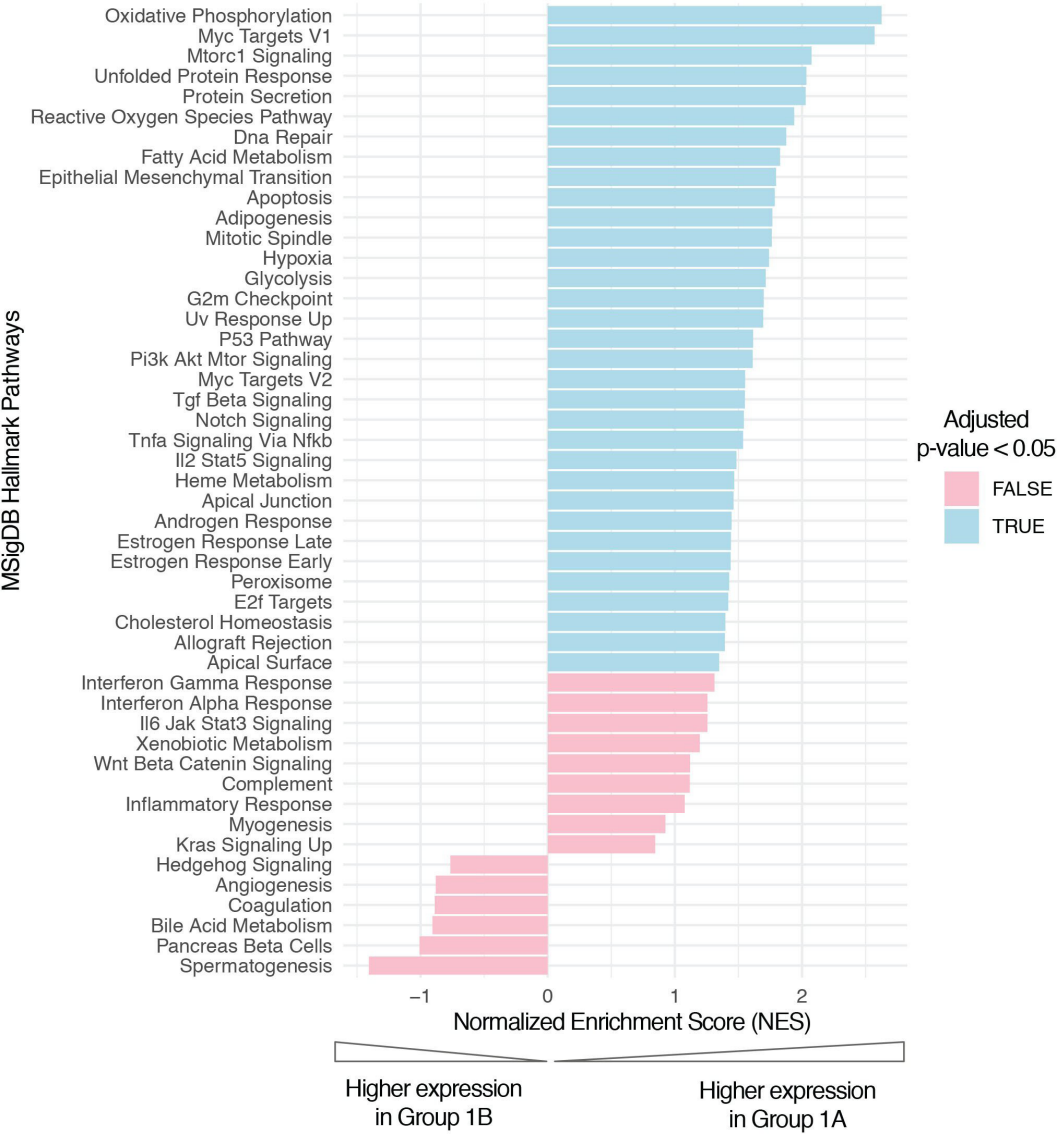
Single-cell RNA sequencing pathways enrichment analysis comparing Group 1A versus Group 1B patients at pre-surgery time point. Bar plot showing Gene Set Enrichment Analysis NES for the MSigDB (Molecular Signatures Database) hallmark pathways. Significant hits (adjusted p value<0.05) are colored in blue.

The scRNAseq revealed activation of hypoxia pathways scRNAseq was performed in tumor samples from patients *S-004*, *S-006*, *S-007*, *S-010* and *S-013*. We compared gene expression profiles of Group 1A patients versus those of Group 1B patients at each time point using Gene Set Enrichment Analysis (GSEA) and the Molecular Signatures Database (MSigDB) hallmark pathways.^28 29^ We found that cancer cells from the Group 1A patients were significantly enriched (adjusted p<0.05) for various pathways compared with cancer cells from Group 1B patients at the pre-surgery time point. These pathways included DNA repair, G2M checkpoint, protein secretion, unfolded protein response, oxidative phosphorylation, reactive oxygen species and hypoxia (figure 4). In addition, we found that hypoxia gene expression signatures previously defined in head and neck cancer,^30 31^ were significantly overexpressed (p value<0.05, false discovery rate [FDR]<0.25) in cancer cells and macrophages from Group 1A patients when compared with those from Group 1B patients, at both day 15 and pre-surgery time points.

### Genomic findings

We evaluated the WES data obtained from baseline, day 15 and pre-surgery tumor biopsies for each patient. In total, there were 27 samples, of which 24 had detectable mutations. The most frequently altered genes were *TP53* in 50% of patients and *NSD1* in 30% of patients (online supplemental figure 6). The most frequently mutated genes of patients from Group 1A were *TP53* (50%, *S-002*, *S-007* and *S-008*), *FAT1* (33%, *S-002* and *S-010*), *MST1* (33%, S-002 and S-010), *NOTCH1* (33%, *S-002* and *S-010*) and *NSD1* (33%, *S-007* and *S-010*). Patients *S-006* and *S-011* (Group 1B) both had *TP53* mutations. Patient *S-011* had missense mutations in *AXL* (L109H) and *HIF1A* (I830V). Patient *S-009*, the only clinical progressor had a truncating mutation in *NSD1* (R1031*). There were no relevant changes in the genomic profiles across samples (baseline vs day 15 vs pre-surgery) within patients.

## Discussion

SNOW is the first study to evaluate the safety and antitumor activity along with the immune and molecular effects of preoperative sitravatinib plus nivolumab in patients with resectable OCSCC. The combination was safe and did not compromise curative intent surgery or adjuvant therapy in this patient population. Sitravatinib and nivolumab led to clinical and pathological responses in almost all patients of our cohort despite a relatively short course of treatment, including one complete and two major pathological responses. CtDNA and cfDNA dynamics correlated with treatment benefit and helped distinguishing patients with different tumor biological behavior following sitravatinib alone versus sitravatinib–nivolumab combination; while multiplexed IHC and scRNAseq revealed differential changes in the expression of immune cell populations including an increased M1 to M2-polarized macrophage ratio following sitravatinib alone, consistent with the predicted immunomodulatory effects of this agent.

To date, two phase 2 studies evaluating neoadjuvant anti-PD-1 agents have shown antitumor activity in patients with resectable HNSCC, with clinical to pathological downstaging and PTR rates (defined as a reduction of viable tumor >50%) ranging from 19% and 22% with one dose of pembrolizumab, to 69% and 40% with two doses of nivolumab, respectively.^18 32^ Similar to what occurred in the recurrent/metastatic setting, PD-L1 expression seemed to enrich for responses in the pembrolizumab study, although this correlation was not observed in the nivolumab study. Anti-PD-1/PD-L1-based combinations with other immuno-oncology agents, targeted therapies or chemotherapy are currently being evaluated in the neoadjuvant space of this disease to increase tumor responses. So far, the addition of ipilimumab to nivolumab did not seem to improve the efficacy in terms of tumor downstaging or PTR (53% vs 69% and 38% vs 40%, respectively) while it did increase toxicity.^32^ In SNOW, clinical to pathological downstaging occurred in 9 out of 10 patients (90%), and a reduction in viable tumor >50% was observed in 50% of the patients regardless of PD-L1 expression, suggesting a potential additive and/or synergistic effect of sitravatinib and nivolumab.

Tumor downstaging as well as PTR are being used as surrogates for survival benefit in patients with resectable HNSCC based on prior evidence with the use of neoadjuvant chemotherapy in this patient population.^33^ Recurrence rates in the pembrolizumab and nivolumab plus/minus ipilimumab studies were lower than expected to historical controls for this disease, and preliminary data from the IMCISION phase II study by Zuur *et al* showed a strong correlation between PTR and both disease-free and overall survival.^34^ In the SNOW study, all patients were alive and with no recurrence after almost 2 years follow-up. These results are encouraging considering that over half of the patients had stage IVA, especially when recurrence rates in this group of patients can be as high as 50% in the first year.^35–37^ The ongoing phase III study of neoadjuvant pembrolizumab (KEYNOTE-689, NCT03765918) in resectable HNSCC evaluating PTR as a co-primary endpoint with overall survival will be key to demonstrate its value as a surrogate measure of survival benefit.

Notwithstanding the ongoing efforts in prospective validation, both clinical to pathological downstaging as well as PTR are still under debate in the field of HNSCC as they can be highly variable and require methodology standardization. For instance, clinical stage is dependent on assessments by the treating surgeon and radiologist as well as on imaging modality used (eg, CT vs PET-CT), which may lead to bias in downstaging rates across studies. Tumor downstaging might not always be representative of the degree of treatment response, particularly in OCSCC where depth of invasion is key to determine the T stage. While PTR could potentially overcome these downstaging limitations, it has its own complexities, such as how to define treatment-related changes and account for viable tumor, or how to identify presence or absence of response in lymphadenopathy in clinical N0 disease. For example, PTR assessment in the nivolumab–ipilimumab study was performed only in primary tumor specimens excluding lymphadenopathy.^32^ Expert consensus and guidelines on how to measure these outcome parameters in the head and neck cancer field are crucial and should be actively pursued alongside the development of neoadjuvant trials.

The multi-omic approach taken in the SNOW study was highly informative on the antitumor activity observed with sitravatinib and nivolumab. Even within a limited patient population, biomarker analysis enabled the elucidation of two distinct patient populations with differential biological changes after sitravatinib alone compared with after the combination of sitravatinib and nivolumab. The collective and integrative use of ctDNA dynamics, scRNAseq and multiplexed IHC of immune cell subsets to distinguish the contribution of components in this study can be extrapolated to other drug combinations of interest. The concomitant increase in cfDNA and decrease in ctDNA in response to sitravatinib is an important illustration of the mechanisms of action of this multi-kinase antiangiogenic tyrosine kinase inhibitors (TKI) in this setting. The spike in cfDNA suggests a non-specific release of nucleic acids into the circulation in response to sitravatinib, which may be a result of cell lysis as is typically observed after surgery or trauma, or alternatively due to cell death by apoptosis or necrosis stimulating DNA release.^38^ The coupling of cfDNA rise with ctDNA reduction would suggest that the latter is the most plausible mechanism at play, consistent with the clinical evidence of rapid tumor shrinkage observed after sitravatinib alone in Group 1A. The increase in M1 to M2 ratio, demonstrated by multiplexed IHC and corroborated by scRNAseq, was consistently seen across Group 1A patients on day 15, suggesting the potential contribution of sitravatinib in the modulation of TME. On the other hand, in patients whose tumors demonstrated clinical shrinkage mainly after receipt of both sitravatinib and nivolumab (Group 1B), an attenuated cfDNA spike at day 15 and a delayed ctDNA reduction until pre-surgery were observed, suggesting that the addition of checkpoint blockade was necessary to induce cell death. This is particularly interesting in patient *S-011* whose tumor PD-L1 expression was <1% by tumor positive score and tumor mutational burden of 1.94 mutations/MB, and thus not expected to respond to nivolumab alone. Interestingly, *S-011’s* tumor harbored an AXL mutation (*AXL L109H*), which may further confer resistance to anti-PD-1 therapy.^39^ The inhibitory effect of sitravatinib on AXL kinase may have increased sensitivity to PD-1 directed therapy, as demonstrated in lung cancer models.^40^ This patient had ctDNA rise on day 15 but achieved ctDNA clearance pre-surgery, suggesting that the combination created an antitumor effect beyond PD-L1 blockade.

Additional exploratory scRNAseq analysis revealed the activation of hypoxia and angiogenesis pathways in both cancer cells and macrophages at day 15 and surgery of Group 1A patients, which might be indicative of sitravatinib’s target effect and antitumor activity as single agent. Moreover, the changes in hypoxia could also suggest a potential synergy with nivolumab, as changes in intratumoral hypoxia following anti-PD-1 therapy have been shown to be predictive of PTR in the IMCISION study.^34^ Overall, these findings are also in line with the results of the FAZA-PET imaging in patients *S-001* (cPR) and *S-002* (mPR), which showed a significant reduction in hypoxia within the tumor area pre-surgery.

The authors acknowledge the limitations of the SNOW study. The limited number of patients and the lack of single-agent arms with nivolumab or sitravatinib alone does not allow evaluation of contribution of components, and also impedes benchmarking with other studies in the same setting and patient population, thus current results should be interpreted with caution. Although the safety profile of the combination appear acceptable, all patients experienced at least one grade 1 or 2 toxicity, and two patients required sitravatinib dose reduction/discontinuation, with one leading to a delay in surgery. The limited number of patients precludes a definitive conclusion in regards to the treatment tolerability in this setting and further evaluation in a larger cohort is recommended. Planned correlative analysis in tumor biopsies could not be performed in all patients at each time point due to sample availability and limited tissue quantity. Despite WES could be conducted in all tumor biopsies, TMB could not be properly evaluated in all patients due to the low purity of the samples analyzed. RNA expression profiling was not feasible due poor DNA quality in some samples.

To the best of our knowledge, this is the first study to characterize the immune and molecular effects of neoadjuvant sitravatinib plus nivolumab in OCSCC. Sitravatinib was able to alter the TME and its immune contexture, and led to deep antitumor responses when combined with nivolumab. It remains unclear whether sitravatinib’s contribution to response might be explained by the changes in macrophage subpopulations or by the inhibition of other kinase-related signaling pathways. Novel technologies helped to dissect the differential molecular effects of sitravatinib versus the sitravatinib–nivolumab combination in patients with HNSCC. These findings might serve as a ‘starting point’ for further evaluation of this drug combination in larger randomized studies and different settings of this disease.

## Methodology

### Study population and trial design

SNOW is a single-center, investigator-initiated, open-label, non-randomized WOO study of preoperative sitravatinib and nivolumab in resectable OCSCC. Eligible patients had previously untreated, pathologically-confirmed OCSCC (floor of mouth, anterior two-third tongue, buccal mucosa, upper and lower gingiva, retromolar trigone and hard palate), deemed surgically resectable (T2-4a, N0-2 or T1 greater than 1 cm-N2 as per American Joint Committee on Cancer (AJCC) eighth edition), with no evidence of distant metastasis (M0). Patients with prior history of tumor-related bleeding or tumor invading major vessels, Eastern Cooperative Oncology Group (ECOG) >2, inadequate organ function and/or history of autoimmune disorders were ineligible.

All patients were planned to receive sitravatinib 120 mg orally one time per day from day 1 until 48 hours before surgery or for a maximum period of 28 days. Nivolumab was given intravenously as a single dose of 240 mg on day 15. Surgery was planned between days 23 and 30 following study treatment initiation. Surgery included resection of all gross disease at the primary site, ipsilateral (and contralateral, in some patients) therapeutic/prophylactic neck dissection, and reconstruction as deemed appropriate. Surgical plan and extent of surgical tumor resection was defined by baseline assessments obtained before study drug administration. Tattooing was performed after the first patient to ensure the pre-treatment clinical extent of the primary tumor was delineated in case of tumor response. Adjuvant radiotherapy alone or with chemotherapy following surgery was planned as per standard of care and institutional protocols based on clinical stage and pathology features. Fresh tumor biopsies and serial blood samples for pharmacodynamic biomarker analyses, as well as clinical photographs of the tumor were collected at baseline, on day 15 prior to nivolumab and at the time of surgery. Optional ^18^FAZA-PET scans for the evaluation of intratumoral hypoxia were performed at baseline and before surgery (online supplemental figure 7).

The primary objective of this study was to evaluate the immune and pharmacodynamic effects of sitravatinib plus nivolumab, including changes in immune cell populations in the tumor, namely T-cell subsets, natural killer cells and myeloid-derived suppressive cell subsets. Secondary objectives were to determine safety and tolerability of the investigational regimen including rate of TRAEs; surgery completion within the planned window and rate of postoperative complications; antitumor activity including clinical and pathological responses and rates of pathological extranodal extension and positive margin (<5 mm).

### Safety and efficacy assessments

AEs were assessed using the Common Terminology Criteria for Adverse Events V.5.0. Surgical complications were assessed using the Clavien-Dindo classification.^27^ Patients were considered evaluable for safety and tolerability if they received at least one dose of either sitravatinib or nivolumab. Clinical response was defined as any reduction in primary tumor volume by physical examination assessed by the treating investigator with supporting photographic documentation. Radiological imaging after study treatment (pre-surgery) was not planned as per protocol unless suspected disease progression or if required prior to surgery based on clinical discretion. Clinical to pathological downstaging was assessed using AJCC eighth edition. PTR assessment of primary tumor and lymph nodes in surgical specimens was evaluated by central pathology review and categorized as follows: complete response (cPTR) if there was no residual viable tumor in the surgical specimen; major response (mPTR) if <10% residual viable tumor; incomplete response (iPTR) for cases with more than 10% residual viable tumor. Determination of treatment-related changes was based on the presence of necrosis, fibrosis, presence of inflammatory cells, and giant cell reaction in the surgical specimen.

### Pharmacodynamic and biomarker analyses

Patients were evaluable for correlative analysis if they had completed at least 11 days of sitravatinib in the first 2 weeks of therapy; received the nivolumab infusion on day 15; had tumor and blood samples available from pre-specified time points that yield acceptable quality and quantity for analysis. A consort diagram of the available samples for each patient and biomarker analysis is provided (online supplemental figure 2).

### Tumor sample collection and processing

The IHC core or tissue fragment from tumor biopsies was placed in a 60 mL collection container with 30 mL of 10% neutral buffered formalin for 12–24 hours, with a maximum fixation time of 96 hours at room temperature. Formalin-fixed paraffin-embedded (FFPE) samples were used for IHC analyses. Remaining fresh core biopsies were stored in normal saline at room temperature before fresh processing (within 4 hours of collection) for flow cytometry analysis. Archival specimens from standard of care diagnostic biopsy performed before inclusion in the study were additionally collected to determine human papillomavirus (HPV) status using linear array PCR. If positive, p16 immunohistochemical staining was additionally performed to confirm HPV-relatedness (p16 classified as positive if nuclear and cytoplasmic staining in ≥70% tumor cells).

### IHC analyses in tumor samples

Multiplexed immuno-fluorescence staining for tumor and immune cell expression markers was performed and quantified in tumor FFPE samples from screening, day 15 and surgery time points using NeoGenomics MultiOmyx technology. This technology evaluates the expression of a panel of 19 biomarkers including arginase 1, CD11b, CD14, CD15, CD16, CD3, CD4, CD8, CD33, CD56, CD68, CD163, HLA-DR, FOXP3, CTLA4, PD-1, PDL1, Ki67 and tumor segmentation marker PanCK. The staining was performed using a single 4 uM FFPE slide. Within each staining round, two cyanine dye-labeled (Cy3, Cy5) antibodies were paired together and recognized two markers. The staining signal was then imaged and followed by novel dye inactivation, enabling repeated rounds of staining. Proprietary deep learning-based workflows were applied to identify individual cells and perform cell classification for all individual markers. Individual cell classification results were combined to generate co-expression summaries and compute spatial distribution statistics for phenotypes of interest. PD-L1 expression was calculated using the combined-positive score and tumor-positive score. See online supplemental material for specifics on flow cytometry analyses.

10.1136/jitc-2021-003476.supp2 Supplementary data

### Personalized ctDNA analysis

cfDNA was extracted from plasma utilizing the Qiagen QIAmp Circulating Nucleic Acid Kit at PM-OICR TGL (Full protocol available at https://tgl.oicr.on.ca/lab-methods/). As previously published, design and application of personalized ctDNA (bespoke, multiplex-PCR, next-generation sequencing) assays were conducted with blinding to clinical data by Natera. For each patient, paired tumor WES data was used to identify and select tumor-specific, clonal, somatic single nucleotide variants that are present in the tumor but absent in the germline.^41 42^ Multiplex-PCR primer pairs targeting up to 16 highly ranked tumor-specific variants were designed as per Natera’s proprietary assay (Signatera). Next, multiplexed targeted PCR was conducted followed by amplicon deep sequencing on an Illumina platform with an average next generation sequencing (NGS) depth per amplicon of >100,000X. A sample was considered ctDNA positive when ≥2 out of the selected target mutations were measured above a predefined confidence threshold. Details of the analytical validation of the assay were previously described.^41^ VAF were determined for each of the Signatera target mutations. Absolute ctDNA levels (MTM per mL) in the plasma were determined by normalizing VAFs by the plasma volume used for each sample. At each time point, MTM per mL was calculated from all tested targets (including undetected targets) that passed a predefined QC threshold. The change in ctDNA from baseline to day 15 (∆ctDNAd15) and to pre-surgery (∆ctDNASRG) was defined as the percentage change in absolute ctDNA levels in plasma at day 15 and pre-surgery compared with baseline, respectively. CtDNA clearance was defined as ctDNA of zero at day 15 or pre-surgery time points, provided that ctDNA was detectable at baseline. Additionally, we collected the change in cfDNA from baseline to day 15 (∆cfDNAd15), defined as the fold change in absolute cfDNA levels in plasma at day 15 since baseline.

### Single-cell RNA sequencing

scRNAseq was performed in a subset of patients only (*S-004, S-006, S-007, S-010 and S-013* (see online supplemental figure 2). Sample processing, sequencing and analyses were conducted at the Princess Margaret Genomics Centre (see online supplemental materials). Raw scRNAseq sequencing reads were mapped against the GRCh38 genome using Cell Ranger V.4. The resulting gene×cell read counts were normalised using SCTransform, and all 15 samples (five patients×time points) were integrated and clustered using Seurat,^43^ as implemented in CReSCENT multisample pipeline.^44^ This pipeline includes batch effect correction, data dimension reduction, cell clustering, differential gene expression detection, and visualization using the Uniform Manifold Approximation and Projection. Cell types were assigned to each cluster, comparing average gene expression profiles for each cluster against manually curated cardiac cell type signatures (online supplemental table Sx), using Gene Set Variation Analysis as previously described.^45^ M1 and M2 macrophages were distinguished from each other manually using expression of markers: M1 (CD68+, CD163–, HLA-DR+) and M2 (CD68+, CD163+). Raw and processed gene×cell read count matrices, and interactive analysis visualizations are provided in CReSCENT (CRES-P29, https://crescent.cloud/ Username: reviewer_snow@crescent.cloud, Password: review_2021). *The project will be made Public on manuscript publication.

To conduct GSEA (V.3.0) for each cell type, we ran a differential gene expression (DGE) analysis for Group 1A patients versus Group 1B patients, using Seurat’s function FindMarkers, as implemented in the CReSCENT pipeline. We included all genes measured in the scRNAseq experiments in the DGE output to allow GSEA to detect coordinate pathway gene expression changes between the two groups of patients.^29^ We used GSEA’s preranked function, inputting as ranks, the DGE -Log10(p value) multiplied by the sign of the average fold change between the two groups of cells; and as gene sets, the MSigDB hallmarks^28^ and two previously reported hypoxia classifiers. Significance was determined by GSEA’s p value (0.05) and FDR (0.25) cutoffs.

WES methods are provided as online supplemental material.

### Statistical analysis

SNOW was a proof-of-concept study, with no specific statistical assumptions at trial onset. Planned accrual was 12 evaluable patients over the course of 24 months. Study was terminated at 10 patients due to COVID-19 pandemic. Overall survival and recurrence-free survival could not be calculated using Kaplan-Meier and competing risk method (considering death without an event as a competing risk), respectively, as all patients are alive with no recurrence as of data cut-off date December 31, 2020. Outcome parameters were defined from date of diagnosis to date of death or last follow-up. Descriptive statistics were used to summarize clinical and biomarker data, with median and range for continuous variables and frequency and percentage for categorical variables. CtDNA measurements were conducted with blinding to clinical data, and patient treatment and clinical data collection were conducted with blinding to ctDNA measurements.

## Data Availability

Data are available in a public, open access repository. All data relevant to the study are included in the article or uploaded as supplementary information. Raw and processed gene × cell read count matrices, and interactive analysis visualizations are provided in CReSCENT (CRES-P24, https://crescent.cloud/ Username: reviewer_snow@crescent.cloud, Password: review_2021). *The project will be made Public upon manuscript publication.

## References

[1] Blume-Jensen P, Hunter T. Oncogenic kinase signalling. Nature 2001;411:355–65. 10.1038/35077225 11357143

[2] Gherardi E, Birchmeier W, Birchmeier C, et al. Targeting MET in cancer: rationale and progress. Nat Rev Cancer 2012;12:89–103. 10.1038/nrc3205 22270953

[3] Lemke G, Rothlin CV. Immunobiology of the TAM receptors. Nat Rev Immunol 2008;8:327–36. 10.1038/nri2303 18421305 PMC2856445

[4] Paolino M, Penninger JM. The role of TAM family receptors in immune cell function: implications for cancer therapy. Cancers 2016;8 10.3390/cancers8100097 27775650 PMC5082387

[5] Akalu YT, Rothlin CV, Ghosh S. TAM receptor tyrosine kinases as emerging targets of innate immune checkpoint blockade for cancer therapy. Immunol Rev 2017;276:165–77. 10.1111/imr.12522 28258690 PMC5381815

[6] Terme M, Pernot S, Marcheteau E, et al. VEGFA-VEGFR pathway blockade inhibits tumor-induced regulatory T-cell proliferation in colorectal cancer. Cancer Res 2013;73:539–49. 10.1158/0008-5472.CAN-12-2325 23108136

[7] Lapeyre-Prost A, Terme M, Pernot S, et al. Immunomodulatory activity of VEGF in cancer. Int Rev Cell Mol Biol 2017;330:295–342. 10.1016/bs.ircmb.2016.09.007 28215534

[8] Sharma P, Hu-Lieskovan S, Wargo JA, et al. Primary, adaptive, and acquired resistance to cancer immunotherapy. Cell 2017;168:707–23. 10.1016/j.cell.2017.01.017 28187290 PMC5391692

[9] Ozao-Choy J, Ma G, Kao J, et al. The novel role of tyrosine kinase inhibitor in the reversal of immune suppression and modulation of tumor microenvironment for immune-based cancer therapies. Cancer Res 2009;69:2514–22. 10.1158/0008-5472.CAN-08-4709 19276342 PMC4370269

[10] Pircher A, Wolf D, Heidenreich A, et al. Synergies of targeting tumor angiogenesis and immune checkpoints in non-small cell lung cancer and renal cell cancer: from basic concepts to clinical reality. Int J Mol Sci 2017;18 10.3390/ijms18112291 29088109 PMC5713261

[11] Rini BI, Plimack ER, Stus V, et al. Pembrolizumab plus axitinib versus sunitinib for advanced renal-cell carcinoma. N Engl J Med 2019;380:1116–27. 10.1056/NEJMoa1816714 30779529

[12] Makker V, Taylor MH, Aghajanian C, et al. Lenvatinib plus pembrolizumab in patients with advanced endometrial cancer. J Clin Oncol 2020;38:2981–92. 10.1200/JCO.19.02627 32167863 PMC7479759

[13] Du W, Huang H, Sorrelle N, et al. Sitravatinib potentiates immune checkpoint blockade in refractory cancer models. JCI Insight 2018;3 10.1172/jci.insight.124184 30385724 PMC6238734

[14] Burtness B. KEYNOTE-048: phase 3 study of first-line pembrolizumab for recurrent/metastatic head and neck squamous cell carcinoma (R/M HNSCC), 2018.

[15] Ferris RL, Blumenschein G, Fayette J, et al. Nivolumab for recurrent squamous-cell carcinoma of the head and neck. N Engl J Med 2016;375:1856–67. 10.1056/NEJMoa1602252 27718784 PMC5564292

[16] Cramer JD, Burtness B, Ferris RL. Immunotherapy for head and neck cancer: recent advances and future directions. Oral Oncol 2019;99:104460. 10.1016/j.oraloncology.2019.104460 31683169 PMC7749717

[17] Stafford M, Kaczmar J. The neoadjuvant paradigm reinvigorated: a review of pre-surgical immunotherapy in HNSCC. Cancers Head Neck 2020;5:4. 10.1186/s41199-020-00052-8 32195008 PMC7077151

[18] Uppaluri R, Campbell KM, Egloff AM. Neoadjuvant and adjuvant pembrolizumab in resectable locally advanced, human Papillomavirus-Unrelated head and neck cancer: a multicenter, phase 2 trial. Clin Cancer Res 2020. 10.1158/1078-0432.CCR-20-1695 PMC7547532 32665297

[19] Melero I, Berman DM, Aznar MA, et al. Evolving synergistic combinations of targeted immunotherapies to combat cancer. Nat Rev Cancer 2015;15:457–72. 10.1038/nrc3973 26205340

[20] Sato-Kaneko F, Yao S, Ahmadi A, et al. Combination immunotherapy with TLR agonists and checkpoint inhibitors suppresses head and neck cancer. JCI Insight 2017;2 10.1172/jci.insight.93397 28931759 PMC5621908

[21] Uehara M, Sano K, Ikeda H, et al. Expression of vascular endothelial growth factor and prognosis of oral squamous cell carcinoma. Oral Oncol 2004;40:321–5. 10.1016/j.oraloncology.2003.08.020 14747064

[22] Brand TM, Iida M, Stein AP, et al. AXL is a logical molecular target in head and neck squamous cell carcinoma. Clin Cancer Res 2015;21:2601–12. 10.1158/1078-0432.CCR-14-2648 25767293 PMC5032632

[23] Hartmann S, Bhola NE, Grandis JR. HGF/Met signaling in head and neck cancer: impact on the tumor microenvironment. Clin Cancer Res 2016;22:4005–13. 10.1158/1078-0432.CCR-16-0951 27370607 PMC6820346

[24] Oliva M, Spreafico A, Taberna M, et al. Immune biomarkers of response to immune-checkpoint inhibitors in head and neck squamous cell carcinoma. Ann Oncol 2019;30:57–67. 10.1093/annonc/mdy507 30462163 PMC6336003

[25] Chinn SB, Myers JN. Oral cavity carcinoma: current management, controversies, and future directions. J Clin Oncol 2015;33:3269–76. 10.1200/JCO.2015.61.2929 26351335 PMC5320919

[26] Farlow JL, Birkeland AC, Swiecicki PL, et al. Window of opportunity trials in head and neck cancer. J Cancer Metastasis Treat 2019;5 10.20517/2394-4722.2018.100 31321307 PMC6638557

[27] Dindo D, Demartines N, Clavien P-A. Classification of surgical complications: a new proposal with evaluation in a cohort of 6336 patients and results of a survey. Ann Surg 2004;240:205–13. 10.1097/01.sla.0000133083.54934.ae 15273542 PMC1360123

[28] Liberzon A, Birger C, Thorvaldsdóttir H, et al. The molecular signatures database (MSigDB) hallmark gene set collection. Cell Syst 2015;1:417–25. 10.1016/j.cels.2015.12.004 26771021 PMC4707969

[29] Subramanian A, Tamayo P, Mootha VK, et al. Gene set enrichment analysis: a knowledge-based approach for interpreting genome-wide expression profiles. Proc Natl Acad Sci U S A 2005;102:15545–50. 10.1073/pnas.0506580102 16199517 PMC1239896

[30] Toustrup K, Sørensen BS, Nordsmark M, et al. Development of a hypoxia gene expression classifier with predictive impact for hypoxic modification of radiotherapy in head and neck cancer. Cancer Res 2011;71:5923–31. 10.1158/0008-5472.CAN-11-1182 21846821

[31] Brooks JM, Menezes AN, Ibrahim M, et al. Development and validation of a combined hypoxia and immune prognostic classifier for head and neck cancer. Clin Cancer Res 2019;25:5315–28. 10.1158/1078-0432.CCR-18-3314 31182433

[32] Schoenfeld JD, Hanna GJ, Jo VY, et al. Neoadjuvant nivolumab or nivolumab plus ipilimumab in untreated oral cavity squamous cell carcinoma: a phase 2 open-label randomized clinical trial. JAMA Oncol 2020;6:1563–70. 10.1001/jamaoncol.2020.2955 32852531 PMC7453348

[33] Bossi P, Lo Vullo S, Guzzo M, et al. Preoperative chemotherapy in advanced resectable OCSCC: long-term results of a randomized phase III trial. Ann Oncol 2014;25:462–6. 10.1093/annonc/mdt555 24401930

[34] Zuur L, Vos JL, Elbers JB, et al. LBA40 neoadjuvant nivolumab and nivolumab plus ipilimumab induce (near-) complete responses in patients with head and neck squamous cell carcinoma: the IMCISION trial. Ann Oncol 2020;31:S1169. 10.1016/j.annonc.2020.08.2270

[35] Huang S-H, O'Sullivan B. Oral cancer: current role of radiotherapy and chemotherapy. Med Oral Patol Oral Cir Bucal 2013;18:e233–40. 10.4317/medoral.18772 23385513 PMC3613874

[36] Chen W-C, Lai C-H, Fang C-C, et al. Identification of high-risk subgroups of patients with oral cavity cancer in need of postoperative adjuvant radiotherapy or chemo-radiotherapy. Medicine 2016;95:e3770. 10.1097/MD.0000000000003770 27258508 PMC4900716

[37] Woody NM, Ward MC, Koyfman SA, et al. Adjuvant chemoradiation after surgical resection in elderly patients with high-risk squamous cell carcinoma of the head and neck: a national cancer database analysis. Int J Radiat Oncol Biol Phys 2017;98:784–92. 10.1016/j.ijrobp.2017.03.019 28602410

[38] Stroun M, Lyautey J, Lederrey C, et al. About the possible origin and mechanism of circulating DNA. Clinica Chimica Acta 2001;313:139–42. 10.1016/S0009-8981(01)00665-9 11694251

[39] Hugo W, Zaretsky JM, Sun L, et al. Genomic and transcriptomic features of response to anti-PD-1 therapy in metastatic melanoma. Cell 2016;165:35–44. 10.1016/j.cell.2016.02.065 26997480 PMC4808437

[40] Tsukita Y, Fujino N, Miyauchi E, et al. Axl kinase drives immune checkpoint and chemokine signalling pathways in lung adenocarcinomas. Mol Cancer 2019;18:24. 10.1186/s12943-019-0953-y 30744655 PMC6369543

[41] Coombes RC, Page K, Salari R, et al. Personalized detection of circulating tumor DNA Antedates breast cancer metastatic recurrence. Clin Cancer Res 2019;25:4255–63. 10.1158/1078-0432.CCR-18-3663 30992300

[42] Bratman SV, Yang SYC, Iafolla MAJ, et al. Personalized circulating tumor DNA analysis as a predictive biomarker in solid tumor patients treated with pembrolizumab. Nat Cancer 2020;1:873–81. 10.1038/s43018-020-0096-5 35121950

[43] Butler A, Hoffman P, Smibert P, et al. Integrating single-cell transcriptomic data across different conditions, technologies, and species. Nat Biotechnol 2018;36:411–20. 10.1038/nbt.4096 29608179 PMC6700744

[44] Mohanraj S, Díaz-Mejía JJ, Pham MD, et al. Crescent: cancer single cell expression toolkit. Nucleic Acids Res 2020;48:W372–9. 10.1093/nar/gkaa437 32479601 PMC7319570

[45] Diaz-Mejia JJ, Meng EC, Pico AR, et al. Evaluation of methods to assign cell type labels to cell clusters from single-cell RNA-sequencing data. F1000Res 2019;8:296. 10.12688/f1000research.18490.1 PMC6720041 31508207

