# Supplementary material for: Antitumor immune effects of preoperative sitravatinib and nivolumab in oral cavity cancer: SNOW window-of-opportunity study
Source: J Immunother Cancer. 2021 Oct 1;9(10):e003476. doi: 10.1136/jitc-2021-003476 (PMC8488751; doi:10.1136/jitc-2021-003476)
Supplement: Supplementary data [file jitc-2021-003476supp001.pdf]

Supplementary data for research article: *Antitumor Immune Effects of Preoperative Sitravatinib and Nivolumab in Oral Cavity Cancer: SNOW Window of Opportunity Study*

Authors: Marc Oliva[1,2], Douglas Chepeha[3], Daniel Araujo[1], Javier Diaz-Mejia[4,5], Peter Olson[6], Amy Prawira[7], Anna Spreafico[1], Scott V. Bratman[8], Tina Shek[9,10], John de Almeida[3], Aaron Hansen[1], Andrew Hope[8], David Goldstein[3], Ilan Weinreb[11], Stephen Smith[11], Bayardo Perez-Ordoñez[11], Jonathan Irish[3], Dax Torti [12,13], Jeffrey Bruce[4,5], Ben X. Wang[5], Anthony Fortuna[12], Trevor Pugh[13,14], Hirak Der-Torossian[6], Ronald Shazer[6], Nickolas Attanasio[15], Qingyan Au[15], Antony Tin[16], Jordan Feeney[16], Himanshu Sethi[16], Alexey Aleshin[16], Isan Chen[6], Lillian L. Siu[1]

Affiliation:

[1]*Division of Medical Oncology and Hematology, Princess Margaret Cancer Centre, University Health Network, Toronto, Canada.*

[2]*Department of Medical Oncology, Catalan Institute of Oncology, L'Hospitalet de Llobregat, Spain.*

[3]*Department of Otolaryngology- Head & Neck Surgery/Surgical Oncology, Princess Margaret Cancer Centre, University Health Network, Toronto, Canada.*

[4] *Department of Computer Science, Princess Margaret Cancer Centre, University Health Network, Toronto, Canada.*

[5]*Department of Immunology, University of Toronto, Toronto, Canada*

[6]*Mirati Therapeutics*

[7]*Department of Medical Oncology, The Kinghorn Cancer Centre, St Vincent's Hospital Sydney, Australia*

[8]*Department of Radiation Oncology, Princess Margaret Cancer Centre, University Health Network, Toronto, Canada.*

[9]Department of Radiation Physics, Princess Margaret Cancer Centre, University Health Network, Toronto, Canada.

[10]Quantitative Imaging for Personalized Cancer Medicine, TECHNA Institute, University Health Network, Toronto, Canada

[11]Department of Pathology, Princess Margaret Cancer Centre, University Health Network, Toronto, Canada.

[12]Tumor Immunotherapy Program, Princess Margaret Cancer Centre, University Health Network, Toronto, Canada.

[13] Ontario Institute for Cancer Research, Toronto, Canada.

[14]Department of Medical Biophysics, University of Toronto, Toronto, Canada

[15] Neogenomics Laboratories, USA.

[16]Natera, Inc. San Carlos, USA

Corresponding Author: Lillian L. Siu, MD.

Division of Medical Oncology and Hematology,

Princess Margaret Cancer Centre.

700 University Avenue, Suite 7-624, Toronto, Ontario M5G 1Z5

T: 416-946-2911 F: 416-946-4467.

Email: lillian.siu@uhn.ca

Methodology

Tumor flow cytometry

Tumor tissues were enzymatically digested using the Miltenyi gentleMACS human tissue dissociation kit (130-095-929, Miltenyi Biotec, Bergisch Gladbach, Germany) following the manufacturer's protocols. For flow cytometry analyses, single-cell suspensions from digested tumor tissues were stained with three immune profiling panels comprised of 16-18 antibodies each (**Supplementary Table 2**). Data were acquired on an LSR Fortessa flow cytometer (5 lasers, 18 channels; BD, NJ, USA) and analyzed using FlowJo software (BD).

Single-cell RNA sequencing (scRNAseq)

Sample processing and sequencing: frozen single-cell suspensions from digested tumor tissues were thawed and stained with cell viability dye (65-0866-18, eBioscience, CA, USA), and live cells were sorted using the Mo-Flo Astrios (Beckman Coulter, CA, USA). 5 x 10^3 live cells/sample were used to generate sequencing libraries using the Chromium Next GEM single cell 5' Kit v2 and Chromium Controller (1000263, 10x Genomics, CA, USA) following the manufacturer's protocol. T cell receptor (TCR) and B cell receptor (BCR) libraries were generated using the Chromium Single Cell Human TCR Amplification Kit (1000252, 10x Genomics) and Chromium Single Cell Human BCR Amplification Kit (1000253, 10x Genomics), respectively, following the manufacturer's protocols. Gene expression libraries were first checked for quality (1 million reads) using the Illumina MiSeq V2 300 cycles nano kit (MS-102-2002, Illumina, CA, USA). 5' gene expression libraries were sequenced using the Illumina NovaSeq 6000 V1 100 Cycles Kit (20027464, Illumina). TCR and BCR libraries were sequenced using Illumina NextSeq 500 150 Cycles Kit (20024907, Illumina). Sample multiplexing was performed based on MiSeq results.

Whole exome sequencing (WES)

WES on fresh tumor from baseline, day 15 and pre-surgery was performed at the Princess Margaret Cancer Centre- Ontario Institute for Cancer Research Translational Genomics Laboratory (PM-OICR TGL). DNA and RNA were co-isolated from snap frozen PBMCs and dissociated tumor cells (see 4.3.5 Single-cell sequencing) using the Qiagen AllPrep DNA/RNA/miRNA Universal Kit according to manufacturer's directions. Tumor-normal exome library pairs were generated from 100 ng of gDNA using a modified KAPA Hyper Prep Kit protocol and Agilent XT V6 + COSMIC probe set and reagents (Full protocol available at https://tgl.oicr.on.ca/lab-methods/). Matched tumor-normal exomes were sequenced to an average depth of 160X and 50X respectively on Illumina HiSeq2500 platform. Sequence reads were analyzed with FastQC and aligned against human genome reference build GrCh37 (hg19) using BwaMem v 0.7.12 to generate raw sequence alignments in BAM format[1]. Preprocessing (PCR-duplicate marking, indel re-alignment and base quality recalibration) was performed using Picard v1.72[2]. Variant call files (VCFs) were generated utilizing GATK v3.6.0[3] and MuTect2[4]. Raw VCF files were annotated with Variant Effect Predictor v92 [5]. Variant calling for Signatera was performed separately based on Natera's proprietary algorithm described in section 4.3.4. Copy number variants were called with Sequenza 2.1[6]. Germline and somatic variants were annotated with GnomAD r2.0.1 allele frequencies[7] to remove common variants and against known cancer hotspots v2 (CancerHotspots.org) at the variant and gene level [7,8]. Analysis included actionable /oncogenic driver analysis using the Precision Oncology Knowledge Base (OncoKB) and pathogenic database ClinVar[9,10] . Tumor mutation burden was calculated as the number of non-synonymous mutations per callable megabase.

FAZA-PET analysis

^{18}F-FAZA PET/CT imaging at screening and before surgery was optional. FAZA (^{18}F-Fluoroazomycin arabinoside) is a PET radiotracer designed to accumulate within hypoxic cells. Two patients chose to participate in the FAZA imaging studies. A total of 5.2 MBq/kg (minimum 250 MBq, maximum 600 MBq)

of FAZA was administered intravenously to patients before each PET scan. All scans were done on a GE Discovery 610 PET/CT system using two bed positions (bin time of 10 minutes) and a field of view of 50x50 cm captured as a 256x256 image matrix (i.e., voxel size 1.95x1.95x3.27 mm). Each imaging session was a 20-minute static scan that took place 120 minutes after FAZA injection. A separate single-bed static scan was conducted to capture the left ventricle of the heart for a robust blood sample reference. Screening and pre-surgery primary tumor and contralateral normal tissue were contoured by a radiation oncologist (SB) along with a spherical region of interest (20 mm diameter) in the left ventricle of the heart. A semi-quantitative analysis compared each patient's FAZA retention in the primary tumor and contralateral normal tissue, using metrics of tumor-max to blood-mean ratio (TBR_{max}), mean uptake, and maximum uptake (in units of standardized uptake values, SUV, as calculated using body weight). Each primary tumor's fractional hypoxic volume (i.e., hypoxic fraction) was also quantified using a personalized SUV threshold derived from the contralateral tissue's mean SUV plus three times its standard deviation[11]. All voxels in the primary tumor with an SUV above this threshold were considered hypoxic, and thus normalized by the primary tumor's total volume to obtain the hypoxic fraction.

Supplementary Table 1. Treatment-related adverse events by patient

Patient ID	Grade 1-2	Grade 3
**S-001**	• Mucositis	
**S-002**	• Anorexia<br>• Fatigue<br>• Mucositis<br>• Rash<br>• Palmar-plantar erythrodysesthesia<br>• Nausea/vomiting<br>• ALT/AST increase	
**S-004**	• Anorexia<br>• Fatigue<br>• Pruritus<br>• Dysphonia<br>• Hypertension<br>• Diarrhea	• Wound infection<br>• Tracheostomy bleeding
**S-006**	• Fatigue<br>• Rash<br>• Hypertension<br>• Diarrhea	
**S-007**	• Anorexia<br>• Fatigue<br>• Dysphonia<br>• Hypertension<br>• Diarrhea<br>• Nausea<br>• ALT/AST increase	
**S-008**	• Dysphonia<br>• ALT/AST increase	
**S-009**	• Anorexia<br>• Fatigue<br>• Mucositis<br>• Other (abdominal pain)<br>• Nausea/vomiting<br>• ALT/AST increase<br>• Thrombocytopenia<br>• Proteinuria<br>• Arthralgias/Myalgias	• Hypertension
**S-010**	• Dysphonia<br>• Hypertension<br>• Diarrhea<br>• ALT/AST increase	
**S-011**	• Fatigue<br>• Rash<br>• Dysphonia	

**S-013**	• Anorexia<br>• Fatigue<br>• Diarrhea<br>• Thrombocytopenia<br>• Lipase increase<br>• Arthralgias/Myalgias	

Supplementary Table 2.

T/B/NK Panel			
**Epitope**	**Channel**	**Company**	**Cat. No.**
CD4	Alexa700	eBioscience	56-0048-82
CD45	APC-Cy7	BioLegend	368516
viability	eF506	eBioscience	65-0866-18
PD-1	BV605	BioLegend	329924
CD3	BUV395	BD	563546
CD8	BUV496	BD	564805
**Myeloid Panel**			
**Epitope**	**Channel**	**Company**	**Cat. No.**
CD16	FITC	eBioscience	11-0168-42
B7-H4	PerCP-Cy5.5	BioLegend	358110
CD14	PE	eBioscience	12-0149-42
CD45	PE-CF594	BD	562279
CD11c	PE-Cy7	eBioscience	25-0116-42
PD-L1	APC	eBioscience	17-5983-42
CD11b	APC-eF780	eBioscience	47-0118-42
HLA-DR	eF450	eBioscience	48-9956-42
CD3	BV510	BioLegend	317332
CD19	BV510	BioLegend	302242
CD56	BV510	BioLegend	318340
viability	eF506	eBioscience	65-0866-18
CD40	BV650	BD	740584
PD-L2	BV711	BD	564258
TREM-1	BV786	BD	743743
CD86	BUV737	BD	564428
**Treg Panel**			
**Epitope**	**Channel**	**Company**	**Cat. No.**
CD39	PerCP-eF710	eBioscience	46-0399-42
FOXP3	PE	eBioscience	12-4777-42
CTLA-4	PE-Cy7	eBioscience	25-1529-42
CD127	Alexa700	BioLegend	351344
viability	eF506	eBioscience	65-0866-18
CD4	BV605	BD	562658
CD45	BV650	BioLegend	304044
CD3	BUV395	BD	563546

Supplementary Figure 1 (online only).

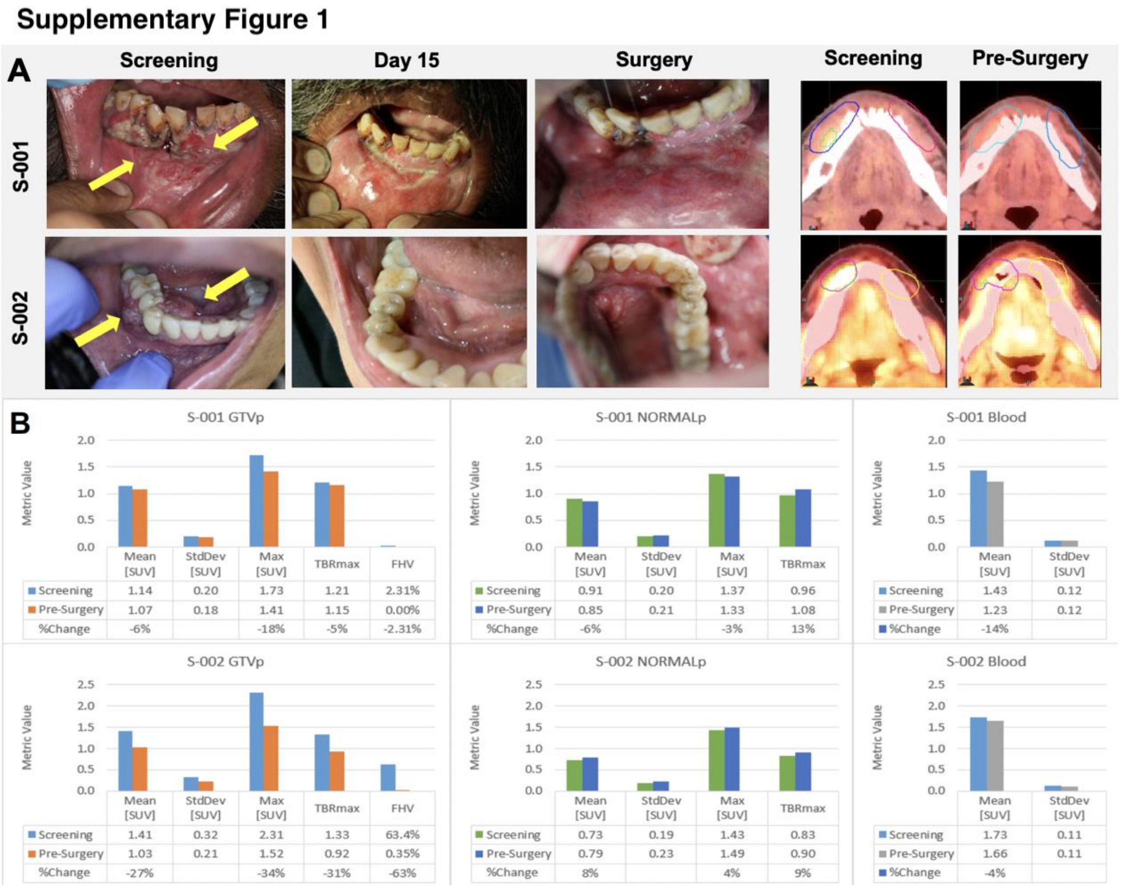

Tumor changes following sitravatinib (day 15) and sitravatinib-nivolumab (surgery) and changes in tumor hypoxia by FAZA-PET imaging presurgery vs baseline in patients *S-001* and *S-002*. **A.** A reduction in tumor extension is observed day 15 and Surgery along with a decrease in hypoxia in the tumor area at surgery vs screening. **B.** Mixed findings among the PET evaluation metrics were observed for *S-001* (non-classifiable), while *S-002* (part of Group 1A) showed a marked reduction in hypoxia across all evaluated metrics within the tumor by the time of surgery. The evolution of FAZA retention at 2 hrs in each patient's primary tumor (GTVp), contralateral normal tissue (NORMALp) and the left ventricle of the heart (Blood) are reported in PET evaluation metrics of mean SUV, standard deviation (StdDev), max SUV, tumor max to mean blood SUV ratio (TBR_{max}), and fractional hypoxic volume (i.e., hypoxic fraction, FHV).

Supplementary Figure 2 (online only).

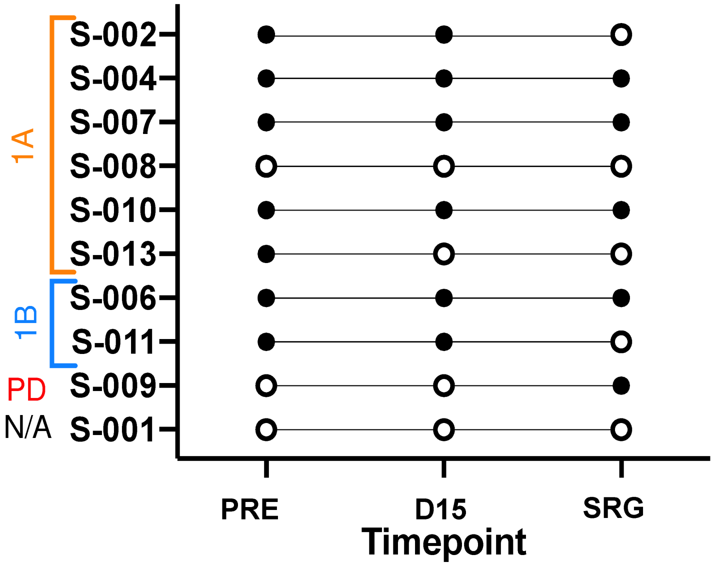

Samples with detectable ctDNA at each timepoint by patient. Black bullet-points equals detectable, while bullet-points equals not detectable. Orange: 1A = responders to sitravatinib; Blue 1B= responders to sitravatinib-nivolumab; Red= non-responder *S-009*; N/A= non-classifiable *S-001*. *Abbreviations: ctDNA= circulating tumor DNA; PRE= baseline; D15= day 15; SRG= pre-surgery.*

Supplementary Figure 3 (online only).

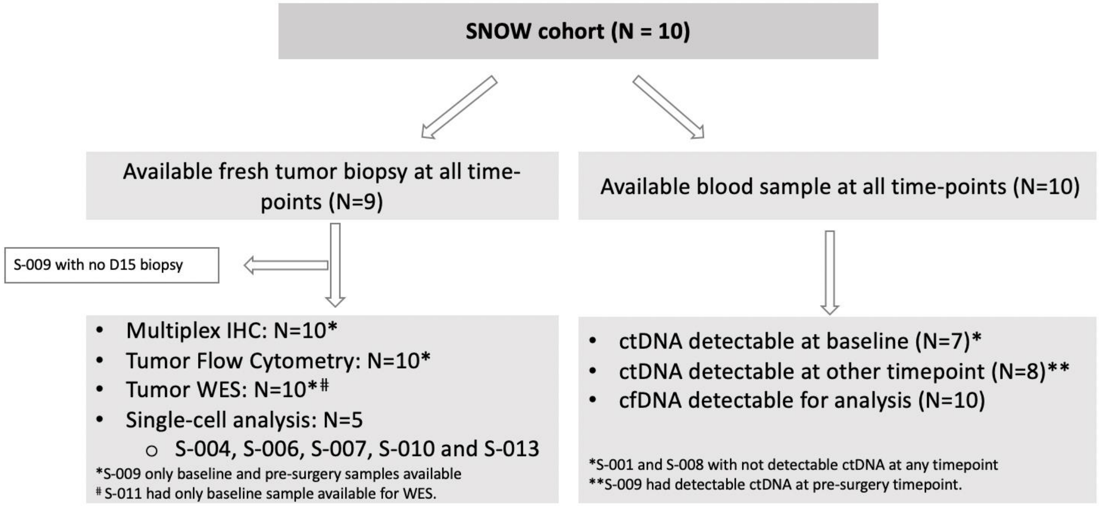

Consort diagram showing the number of available samples and timepoints for each biomarker analysis.

Abbreviations: IHC= immunohistochemistry; WES= whole exome sequencing; ctDNA= circulating tumor DNA; cfDNA= cell-free DNA.

Supplementary Figure 4 (online only)

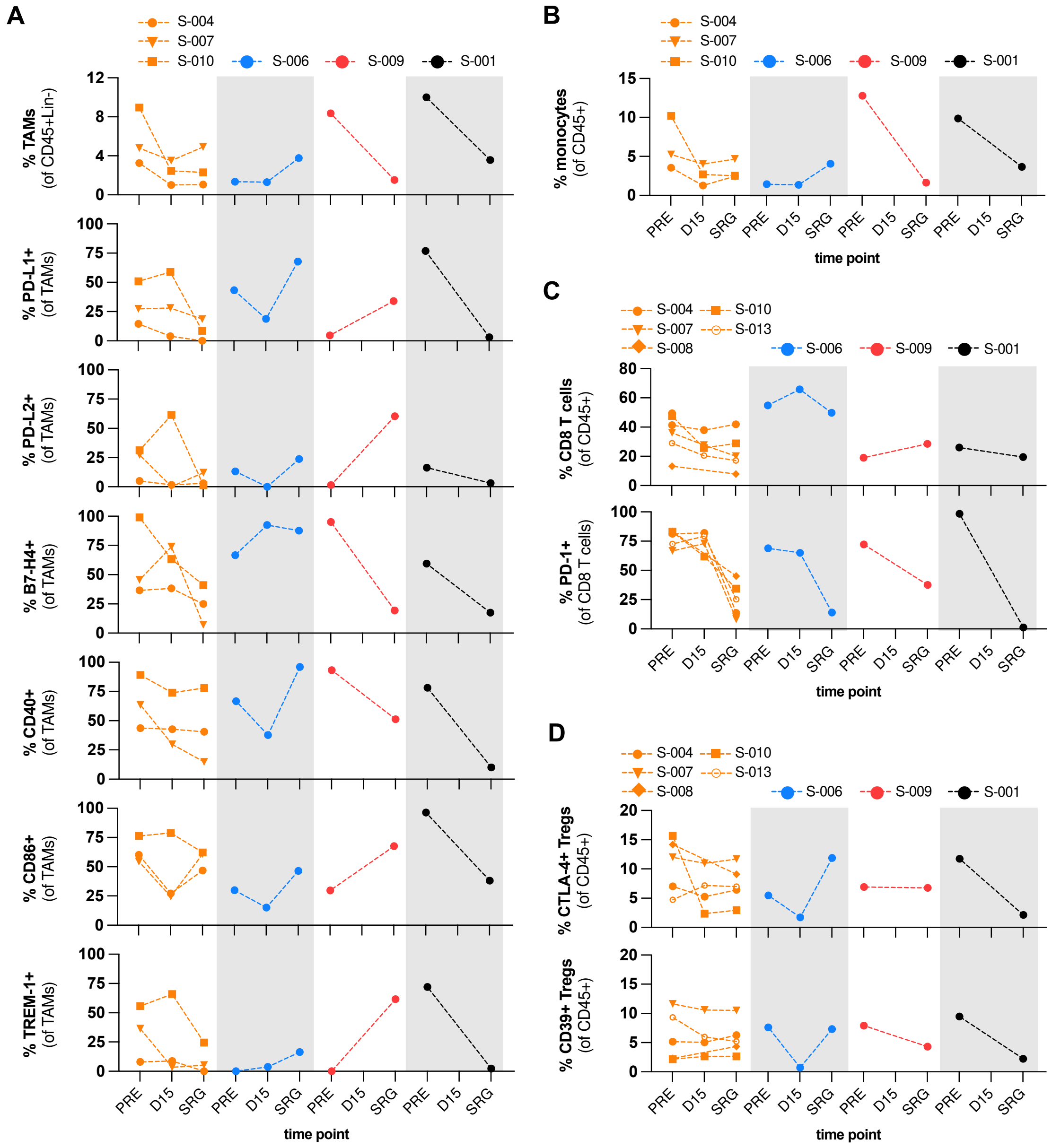

Immune correlates by flow cytometry. A) The frequency of tumor-associated macrophages (TAMs; HLA-DR+CD14+CD11b+) and the expression of inhibitory (PD-L1, PD-L2, B7-H4) and co-stimulatory (CD40, CD86) markers, and triggering receptor expressed on myeloid cells 1 (TREM-1). The frequency of B)

monocytes (CD14+CD16-) and C) CD8+ T cells and PD-1 expression on CD8 T cells. Nivolumab treatment at day 15 reduces detectable PD-1 on CD8+ T cells at surgery (anti-PD-1 antibody is clone EH12.2H7). D) The frequency of CTLA-4+ and CD39+ regulatory T cells (Tregs; CD4+FOXP3+CD127-). Inconsistent changes in CTLA-4+ and CD39+ regulatory T cells are observed in group 1A patients, while for patient S-006 we can observe a reduction at D15 followed by an increase at SRG in S-006. Orange, Group 1A (S-004, S-007,S-008,S-010, S-013); Blue, Group 1B (S-006); Red, S-009; Black, S-001. Lin = CD3, CD19, CD56.

Supplementary Figure 5 (online only).

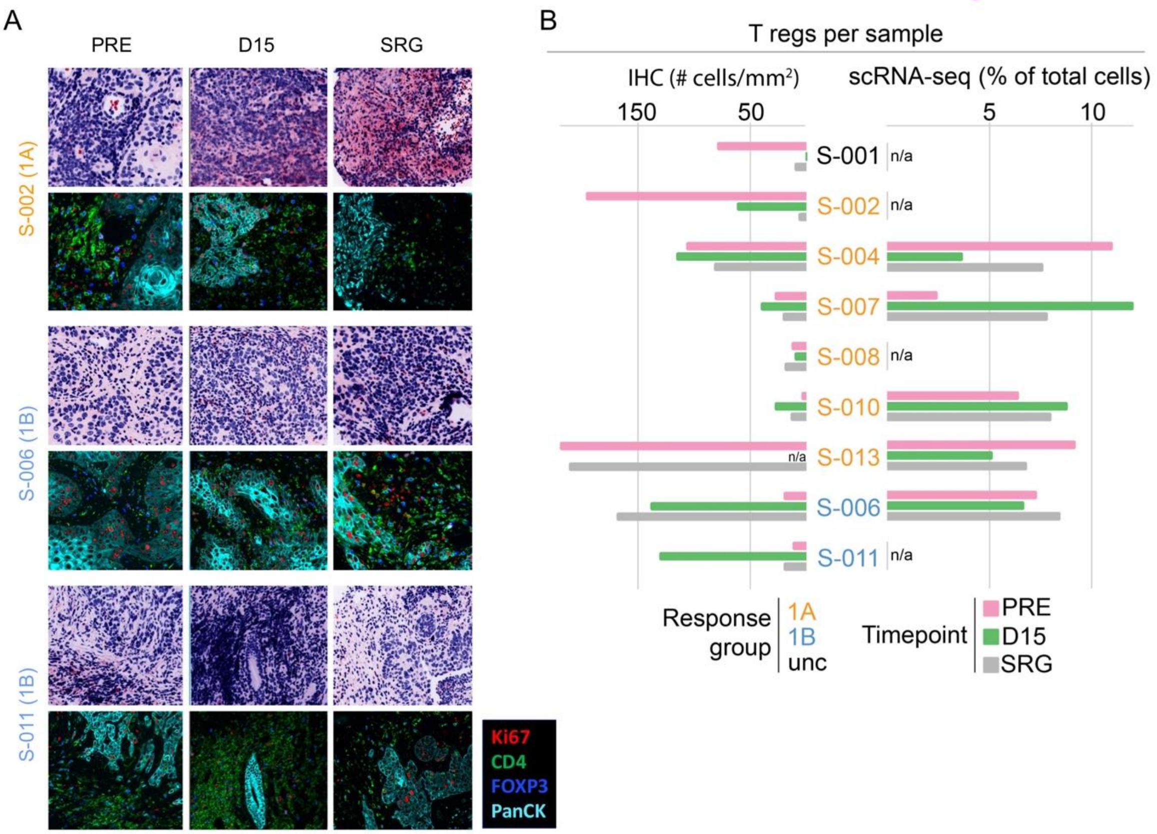

A. Multiplexing immuno-fluorescence (IF) staining in tumor biopsies at pre-treatment (PRE), day 15 (D15) and pre-surgery (SRG) using NeoGenomics MultiOmyx^{TM} panels showing changes in T-regulatory cells (T regs) (CD3+ CD4+ FoxP3+ cells) shown in yellow. Upper images show hematoxylin and eosin staining of tissue sample. B. Quantitative changes in Tregs at D15 and at SRG vs PRE for each patient detected using multiplex IHC and scRNA-seq measurements. Orange: 1A = responders to sitravatinib; Blue 1B= responders to sitravatinib-nivolumab; Black= Unclassifiable. *Abbreviations: PRE= baseline; D15= day 15; SRG= pre-surgery; IHC= immunohistochemistry;* scRNA-seq= single-cell RNA sequencing; n/a= not applicable due to lack of sample.

Supplementary Figure 6. Oncoprint (online only).

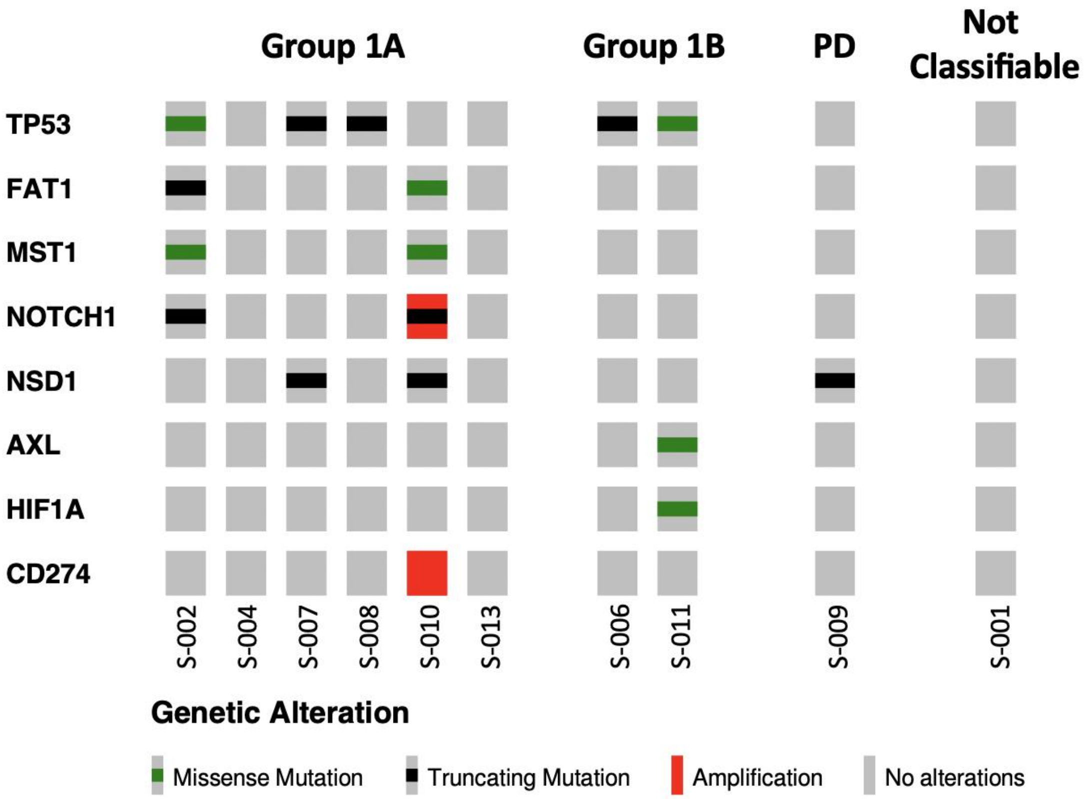

Oncoprint showing most frequently altered and genes of interest in this cohort.

Supplementary Figure 7 (online only). Study Design

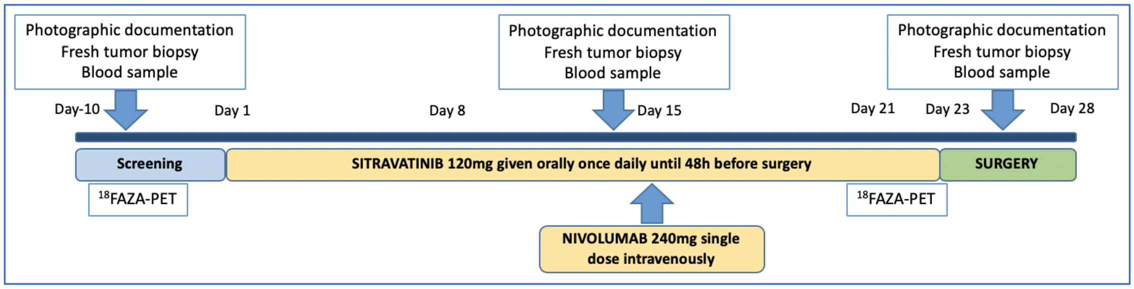

Sitravatinib 120 mg is given orally once daily from day 1 until 48h before surgery or for a maximum period of 28 days. Nivolumab 240mg is given intravenously on day 15 for one dose only. Surgery is planned between days 23-30 following study treatment initiation. Sample collection for pharmacodynamic and pharmacokinetic analyses performed at baseline, prior to day 15 and pre-surgery. Optional FAZA-PET performed at baseline and pre-surgery after treatment completion.

Methodology References

1. Li H, Durbin R: Fast and accurate short read alignment with Burrows-Wheeler transform. Bioinformatics 25:1754-60, 2009
2. Institute B: Picard Toolkit. . Broad Institute, GitHub Repository, 2019
3. McKenna A, Hanna M, Banks E, et al: The Genome Analysis Toolkit: a MapReduce framework for analyzing next-generation DNA sequencing data. Genome Res 20:1297-303, 2010
4. Benjamin D, Sato T, Cibulskis K, et al: Calling Somatic SNVs and Indels with Mutect2. bioRxiv:861054, 2019
5. McLaren W, Gil L, Hunt SE, et al: The Ensembl Variant Effect Predictor. Genome Biol 17:122, 2016
6. Favero F, Joshi T, Marquard AM, et al: Sequenza: allele-specific copy number and mutation profiles from tumor sequencing data. Ann Oncol 26:64-70, 2015
7. Lek M, Karczewski KJ, Minikel EV, et al: Analysis of protein-coding genetic variation in 60,706 humans. Nature 536:285-91, 2016
8. Chang MT, Asthana S, Gao SP, et al: Identifying recurrent mutations in cancer reveals widespread lineage diversity and mutational specificity. Nat Biotechnol 34:155-63, 2016
9. Memorial Sloan Kettering Cancer Centre QD: OncoKB, 2016
10. Landrum MJ, Lee JM, Benson M, et al: ClinVar: public archive of interpretations of clinically relevant variants. Nucleic Acids Res 44:D862-8, 2016
11. Mortensen LS, Johansen J, Kallehauge J, et al: FAZA PET/CT hypoxia imaging in patients with squamous cell carcinoma of the head and neck treated with radiotherapy: results from the DAHANCA 24 trial. Radiother Oncol 105:14-20, 2012
