# Supplementary material for: Antitumor immune effects of preoperative sitravatinib and nivolumab in oral cavity cancer: SNOW window-of-opportunity study
Source: J Immunother Cancer. 2021 Oct 1;9(10):e003476. doi: 10.1136/jitc-2021-003476 (PMC8488751; doi:10.1136/jitc-2021-003476)
Supplement: Supplementary data [file jitc-2021-003476supp002.pdf]

Drug Substance	**Sitravatinib and Nivolumab**
Protocol Number /Version	**SNOW-001 / Version 04**
Date	**01 Jul 2020**

Sitravatinib (MGCD516) and Nivolumab in Oral cavity cancer Window opportunity study (SNOW)

**Leading Institution:**	Princess Margaret Cancer Centre
**Principal Investigator:**	Dr Lillian Siu

Sub-Investigators:

Dr Douglas Chepeha
Dr Scott Bratman
Dr Marc Oliva
Dr Aaron Hansen
Dr Anna Spreafico
Dr Raymond Jang
Dr Jonathan Irish
Dr John de Almeida
Dr David Goldstein
Dr Ralph Gilbert
Dr Patrick Gullane
Dr Dale Brown
Dr Bayardo Perez-Ordonez
Dr Ilan Weinreb
Dr Pamela Ohashi
Dr Linh Nguyen
Dr Tracy McGaha
Dr Ben Wang

Corresponding Investigator:

Dr Lillian Siu
Princess Margaret Cancer Centre
700 University Avenue, 7-624
Toronto, ON Canada M5G 1Z5
Ph. 1-416-946-2911
Fax. 1-416-946-4467
Email: Lillian.Siu@uhn.ca

CONFIDENTIAL

TABLE OF CONTENTS

Title page 1
TABLE OF CONTENTS 2
LIST OF TABLES 5
LIST OF ABBREVIATIONS 6
Clinical Study Protocol Synopsis 8
1. Introduction 14
1.1 Rationale for conducting this study 15
1.1.1 Rationale for study design 15
1.1.2 Rationale for the combination of Sitravatinib and Nivolumab 16
1.1.3 Rationale for choice of primary biomarker endpoints 16
1.1.4 Rationale for tumor hypoxia assessment with ^{18}FAZA PET 17
1.2 Background for investigational agents 18
1.2.1 Background information for Sitravatinib 18
1.2.2 Background information for Nivolumab 23
2. Study Objectives 28
2.1 Primary objective 28
2.2 Secondary objectives 28
2.3 Exploratory objectives 28
2.4 Study endpoints 28
3. Study plan and procedures 30
3.1 Overall study design 30
3.2 Statistical design 30
3.2.1 Determination of sample size 30
3.2.2 Planned analyses 30
3.2.3 Evaluability 31
3.3 Study periods 31
3.3.1 Screening period 31
3.3.2 Treatment period 31
3.3.3 Follow-up period 31
3.4 End of study definition 31
3.5 Early study termination 31
4. Subject Selection Criteria 34
4.1 Inclusion criteria 34
4.2 Exclusion criteria 35
5. Study conduct 37
5.1 Subject enrollment 37
5.2 Patient numbering 38
5.3 Treatments 38
5.3.1 Identity of investigational product(s) 38
5.3.2 Doses and treatment regimens 39
5.3.3 Treatment duration 39
5.3.4 Ancillary treatments 39
5.3.5 Dose modifications and delays 40
5.3.6 Therapy after completion of protocol treatment 44
5.3.7 Follow-up for toxicities 44

5.4 Study drug handling 44
5.4.1 Sitravatinib preparation, packaging and labelling 44
5.4.2 Nivolumab preparation, packaging and labelling 45
5.4.3 Drug supply and storage 46
5.5 Concomitant and post-study treatment(s) 46
5.5.1 Permitted concomitant therapies 46
5.5.2 Prohibited concomitant therapy 47
Cytochrome P-450 Substrates 47
Medications for Gastric pH 48
Transporter Substrates and Inhibitors 48
Medications that Prolong QTc 48
5.5.3 Rescue Medications & Supportive Care 48
5.5.4 Diet/Activity/Other Considerations 49
5.6 Treatment compliance 51
5.6.1 Accountability 51
5.6.2 Disposal and destruction 51
5.7 Discontinuation of investigational product 51
5.7.1 Procedures for discontinuation of a subject from investigational product 51
5.7.2 Replacement policy 52
5.7.3 Lost to follow up 53

6. Collection of study variables 53
6.1 Study flow and visit schedule 53
6.1.1 Screening 53
6.1.2 Treatment period 54
6.1.3 Safety follow up period 54
6.1.4 Disease progression follow up period 54
6.2 Clinical assessments 58
6.2.1 Vital signs 58
6.2.2 Height and weight 58
6.2.3 Performance status 58
6.2.4 Laboratory evaluations 59
6.2.5 Cardiac assessments 59
6.2.6 Photographic documentation 59

7. Safety monitoring and reporting 59
7.1 Adverse events 59
7.1.1 Definitions and reporting 59
7.1.2 Laboratory test abnormalities 60
7.2 Serious adverse events 61
7.2.1 Definitions 61
7.2.2 SAE Reporting 62
7.2.3 Other Events Requiring Immediate Reporting 62
7.2.4 Expedited Reporting to Tumor Immunotherapy Program and Mirati 65
7.2.5 Pregnancy reporting by Investigator to Mirati 65
7.2.6 Reporting to Health Canada 66
7.3 Reporting of adverse events to the Research Ethics Board (REB) 66
7.4 Adverse Event updates 66
7.5 Adverse events of special interest 66

8. Correlative Studies 67

8.1 Pharmacokinetic assessments 67
8.1.1 Sample handling, labeling, and shipping instructions 68
8.2 Biomarker assessments 68
8.3 Preliminary activity assessments 71
8.4 ^{18}FAZA PET assessments 71

9. Ethical and regulatory requirements 71
9.1 Ethical conduct of the study 71
9.2 REB composition 72
9.3 Initial approval 72
9.4 Annual re-approvals 72
9.5 Amendments / Revisions 72
9.6 Informed consent document 72
9.7 Serious adverse events, safety updates, and Investigator brochure updates 72
9.8 Warnings and precautions 73

10. Publication policy 73
10.1 Authors 73
10.2 Responsibility for publication 73
10.3 Submission of materials for presentation or publication 73

11. Data collection and management 73
11.1 Data confidentiality 73
11.2 Source documents 74
11.3 Direct access to source data and documents 74
11.4 Retention of patient records and study files 74

12. Study Management 75
12.1 Training of study site personnel 75
12.2 Monitoring of the study 75

13. Data management guidelines 75
13.1 Case report form completion 75
13.2 Case report form submission schedule 75

14. Appendices 76
14.1 Pre-existing autoimmune diseases and immune deficiencies 76
14.2 Toxicity management guidelines 77
14.2.1 Immune-mediated toxicities 77
14.2.2 Management algorithms for Nivolumab immune-mediated toxicities 79
14.2.3 Infusion-related reactions 86
14.2.4 Non-immune mediated toxicities 86
14.2.5 Sitravatinib (MGCD516) adverse events of interest 87
14.3 Additional Safety Guidance 88
14.3.1 Assessment of Severity 88
14.4 Medications or substances to be avoided or used with caution during study treatment 90
14.4.1 Drugs that may prolong QTc inter 90
14.4.2 Transporter substrates 91

15. LIST OF References 92

LIST OF TABLES

Table 1. Objectives and related endpoints 30
Table 2. Dose and treatment schedule 39
Table 3. Sitravatinib Sequential Dose Reductions for Individual Patients 40
Table 4. General management of Sitravatinib Non-haematological toxicities 39
Table 5. Sitravatinib Dose Modification for Hypertension 40
Table 6. Highly effective methods of contraception (<1% failure rate). 50
Table 7. Schedule of Assessments. 56
Table 8. Eastern Cooperative Oncology Group (ECOG) performance status 58
Table 9. Tumor-based biomarker collection 69
Table 10. Blood-based biomarker collection 70
Table 11. Case report form completion schedule 75
Table 12. Autoimmune disease 76
Table 13. Overall management for immune-related adverse events 78
Table 14. Infusion-related reactions management guidelines 86
Table 15. Non-immune mediated toxicities management guidelines. 87
Table 16. Adverse Drug Reactions Associated with Sitravatinib 88
Table 17. NCI CTCAE version 5.0 88
Table 18. Event relationship to protocol procedure. 90

LIST OF FIGURES

Figure 1. Sitravatinib increases activity of anti-pd1 in singeneic mouse models 19
Figure 2. Study schema. 31

LIST OF ABBREVIATIONS

AE	Adverse Event
AESI	Adverse Event of Special Interest
ALT	Alanine Aminotransferase
APC	Antigen Presenting Cell
AST	Asparate Aminotransferase
Cmax	Maximum Concentration
CRA	Clinical Research Associate
CRF	Case Report Form
CTA	Clinical Trials Application
CTCAE	Common Terminology Criteria for Adverse Events
DC	Dendritic Cell
DCR	Disease Control Rate
DLT	Dose Limiting Toxicity
eCRF	Electronic CRF
FACS	Fluorescence-Activated Cell Sorting
GGT	Gamma-Glutamyltransferase
GLP	Good Laboratory Practice
HBV	Hepatitis B Virus
HCV	Hepatitis C Virus
HLA	Human Leukocyte Antigen
HPLC	High-Performance Liquid Chromatography
IB	Investigator Brochure
ICF	Informed Consent Form
ICH	International Council for Harmonization of Technical Requirements for Pharmaceuticals for Human Use
ICI	Immune Checkpoint Inhibitor
IP	Investigational Product
MDSC	Myeloid-derived Suppressor Cells
MHC	Major Histocompatibility Complexes
MTD	Maximum tolerated dose
NCI	National Cancer Institute
NOAEL	No Observed Adverse Effect Level
NSCLC	Non-small Cell Lung Cancer
ORR	Overall Response Rate
OS	Overall Survival
PD	Pharmacodynamic
PD-1	Programmed Death-1
PD-L1	Programmed Death Ligand-1
PPE	Palmar-Plantar Erythrodysesthesia.
PK	Pharmacokinetic
TIP	Tumor Immunotherapy Program
QD	Once daily

Q2W	Every 2 weeks
REB	Research Ethics Board
RECIST	Response Evaluation Criteria for Solid Tumors
RP2D	Recommended Phase II Dose
RTKs	Receptor tyrosine kinases
SAE	Serious Adverse Event
SCCHN	Squamous Cell Carcinoma of the Head and Neck
SCCOC	Squamous Cell Carcinoma of the Oral Cavity
SN	Subject Number
SUSAR	Suspected Unexpected Serious Adverse Reaction
TAA	Tumor-associated Antigen
TCR	Tcell Receptor
Treg	Regulatory T-cells
TME	Tumor Microenvironment

CLINICAL STUDY PROTOCOL SYNOPSIS

Title: **S**itravatinib and **N**ivolumab in **O**ral cavity cancer **W**indow opportunity study (SNOW).

Clinical Phase: Window of Opportunity Study

Study Type: Investigator Initiated Study

Protocol Number/Date: SNOW-001 V03/ 11 Jul 2018

Principal Investigator: Dr. Lillian Siu

Sub-Investigators: Dr Douglas Chepeha, Dr Scott Bratman, Dr Marc Oliva, Dr Aaron Hansen, Dr Anna Spreafico, Dr Raymond Jang, Dr Jonathan Irish, Dr John de Almeida, Dr David Goldstein, Dr Ralph Gilbert, Dr Patrick Gullane, Dr Dale Brown, Dr Bayardo Perez-Ordonez, Dr Ilan Weinreb, Dr Pamela Ohashi, Dr Linh Nguyen, Dr Tracy McGaha, Dr Ben Wang.

Institutions:
Princess Margaret Cancer Centre, University Health Network, Toronto, Ontario, Canada

Study duration / dates: 2 year / Q2 2018 – Q1 2020

Primary objective:
To evaluate the pharmacodynamic and immune effects of pre-operative therapy with Sitravatinib and Nivolumab on patients with squamous cell carcinoma of the oral cavity.

Secondary objectives:

1. To evaluate the safety and tolerability of pre-operative therapy with Sitravatinib and Nivolumab in patients with squamous cell carcinoma of the oral cavity.
2. To evaluate the preoperative clinical activity of the combination of Sitravatinib and Nivolumab in patients with squamous cell carcinoma of the oral cavity.
3. To determine Sitravatinib plasma levels alone and in combination with Nivolumab.

Exploratory objectives:

1. To evaluate the dynamic changes in immune cell activation and/or suppression using flow cytometry, DNA/RNA sequencing, and FACS sorting.
2. To evaluate the dynamic changes in intratumoral hypoxia with pre-operative Sitravatinib and Nivolumab therapy using ^{18}FAZA PET.

Planned number of subjects:
We plan to enroll 12 patients who are evaluable for correlative studies. Assuming 20% non-evaluable rate, an estimated total of 15 patients will be enrolled.

Study design and plan:
This is a single center, open-label, non-randomized, pre-operative window of opportunity study for patients with resectable squamous cell carcinoma of the oral cavity who are considered suitable for curative-intent surgical resection, with pre-operative Sitravatinib and Nivolumab. A total of 12 patients who are evaluable for correlative studies, are planned for enrollment.

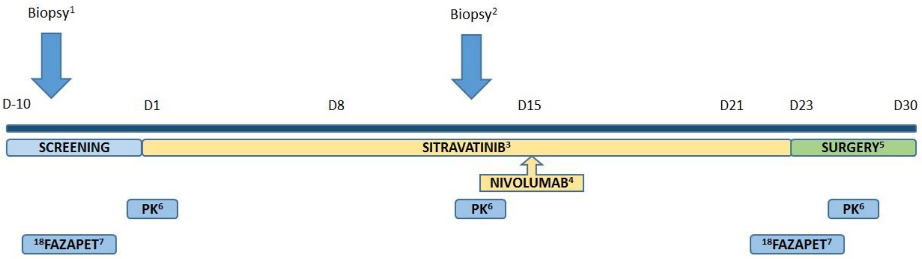

1. Baseline tumor biopsy to be performed ≤ 10 days prior to the start of Sitravatinib therapy.
2. On-treatment tumor biopsy will be performed within 48h hours prior to the planned Day 15 Nivolumab.
3. Patients will start therapy with Sitravatinib within 10 days of study enrollment. Sitravatinib will be given at 120mg once daily on a continuous basis until 48 hours before planned surgery, or for a maximum period of 28 days.
4. Nivolumab will be given as a single infusion at a dose of 240mg, over a period of 30 minutes on Day 15 of the study.
5. Surgery will be performed within 23-30 days following the start of study treatment, with at least 48 hours after the last dose of Sitravatinib.
6. Pharmacokinetic sample collections are to be performed on a single time-point at baseline, on the day of on-treatment biopsy and on the day of surgery.
7. ^{18}FAZA PET testing is optional, however is strongly encouraged to be performed within screening and pre-surgery assessment periods.

Inclusion criteria:
Informed consent

1. Signed written and voluntary informed consent.
2. Patient must be willing and able to comply with scheduled visits, treatment plan, laboratory tests and other study procedures.
3. Age ≥ 18 years, male or female.

Disease characteristics

4. Patient must be diagnosed with histologically confirmed squamous cell carcinoma of the oral cavity (SCCOC) (floor of mouth, anterior 2/3 tongue, buccal mucosa, upper and lower gingiva, retromolar trigone and hard palate)

previously untreated, considered resectable by the head and neck treating surgeon (T2-4a, N0-2 or T1 - greater than 1 cm - N2, M0; without evidence of distant metastasis).

5. Patient must be willing and able to provide 2 fresh tumor biopsies for histopathological and biomarker evaluation: one at baseline and one after treatment with Sitravatinib but prior to treatment with Nivolumab. Archival tissue sample will be requested if available.
6. No anti-neoplastic treatment is allowed between the time from obtaining baseline tumor specimen and enrollment.

Patient characteristics

7. ECOG performance status 0-1.
8. Patient must have adequate organ function as determined by the following:
   - Renal function:
     i. Serum creatinine ≤ 1.5 ULN (upper limit of normal range) or a calculated creatinine clearance of ≥ 50mL/min using the following formula:

        Creatinine clearance = [(140-age) x wt (kg) x Constant*] / creatinine (umol/L)

        *Constant = 1.23 for men, and 1.04 for women
   - Bone marrow function (without hematopoietic growth factors or transfusion):
     i. Absolute neutrophil count (ANC) ≥ 1.0 x 10^9/L
     ii. Leukocytes ≥ 2.0 x 10^9/L
     iii. Hemoglobin ≥ 90 g/L or ≥ 9g/dL
     iv. Platelets ≥ 100 x 10^9/L
   - Liver function:
     i. Total bilirubin ≤ 1.5 x ULN or ≤ 3 x ULN for patients with Gilbert Syndrome.
     ii. Aspartate aminotransferase (AST/SGOT) and alanine aminotransferase (ALT/SGPT) ≤ 2.5 x ULN
   - Cardiac function:
     i. A normal left ventricular ejection fraction (LVEF) of ≥ 50% by a MUGA scan performed within 4 weeks of the study commencement.
9. Evidence of post-menopausal status, or negative urinary or serum pregnancy test for female pre-menopausal patients. Women will be considered post-menopausal if they have been amenorrheic for 12 months without an alternative medical cause. The following age-specific requirements apply:
   - Women <50 years of age would be considered post-menopausal if they have been amenorrheic for 12 months or more following cessation of exogenous hormonal treatments and if they have luteinizing hormone and follicle-stimulating hormone levels in the post-menopausal range for the institution or underwent surgical sterilization (bilateral oophorectomy, bilateral salpingectomy or hysterectomy).
   - Women ≥50 years of age would be considered post-menopausal if they

have been amenorrheic for 12 months or more following cessation of all exogenous hormonal treatments, had radiation-induced menopause with last menses >1 year ago, had chemotherapy-induced menopause with last menses >1 year ago, or underwent surgical sterilization (bilateral oophorectomy, bilateral salpingectomy or hysterectomy).

10. Subject is willing and able to comply with the protocol for the duration of the study including undergoing treatment and scheduled visits and examinations including follow up.

Exclusion criteria:

1. Primary site of head and neck carcinoma unknown, lip, skin, or outside the oral cavity.
    - Patients with tumors that invade major vessels or are within ≤ 3 mm of the carotid artery, as shown unequivocally by imaging studies.
2. Patients with any prior history of clinically significant bleeding related to the current head and neck cancer.
3. Patients with a history of gross hemoptysis (bright red blood of ½ teaspoon or more per episode of coughing) ≤ 3 months prior to enrollment.
4. Prior or concurrent radiation therapy to tumor at site of planned resection.
5. Any concurrent chemotherapy, biologic, immunologic or hormonal therapy for cancer treatment.
    - Concurrent use of hormones for non-cancer-related conditions (eg, insulin for diabetes and hormone replacement therapy) is acceptable.
6. Current or prior use of immunosuppressive medication within 14 days prior to starting dosing. The following are exceptions to this criteria:
    - Intranasal, inhaled, topical steroids or local steroid injections (eg, intra-articular injection).
    - Adrenal replacement steroid > 10 mg daily prednisone equivalent are permitted in the absence of active autoimmune disease.
    - Steroids as premedication for hypersensitivity reactions (eg, computed tomography scan premedication).
7. Active or documented history of autoimmune disease within 2 years before screening, including:
    - Active or prior documented inflammatory bowel disease (eg. Crohn's disease, ulcerative colitis)
    - Patients with vitiligo, resolved childhood asthma/atopy, type I diabetes mellitus, Grave's disease, Hashimoto's disease, or psoriasis not requiring systemic steroids and/or immunosuppressive agents within the past 2 years, are not excluded.
8. History of primary immune deficiency.
9. History of stroke or transient ischemic attack within the previous 6 months.
10. History of uncontrolled hypertension (> 150 mm Hg systolic or > 100 mm Hg diastolic) on multiple observations despite standard of care treatment.
11. Any of the following cardiac abnormalities:

- Unstable angina pectoris,

- Previous history of congestive heart failure of any NYHA Class,
- QTc >480 milliseconds,
- Left ventricular ejection fraction (LVEF) < 50%.

12. Concomitant medication known to cause prolonged QT that cannot be discontinued or changed to a different medication prior to enrollment.
13. History of organ transplant that requires use of immunosuppressive medications.
14. Known allergy or reaction to any component of Sitravatinib and/or Nivolumab formulation.
15. Subjects who are known to be human immunodeficiency (HIV) positive.
16. Has a known history of or is positive for active hepatitis B (defined as hepatitis B surface antigen [HBsAg] reactive) or hepatitis C (defined as HCV RNA [qualitative] is detected).
    - HBV DNA must be undetectable and HBsAg negative at Screening Visit.
    - Participants who have had definitive treatment for HCV are permitted if HCV RNA is undetectable at Screening Visit.
17. Female patients who are pregnant or breast-feeding.
18. Uncontrolled intercurrent illness including, but not limited to, ongoing or active clinically significant infection requiring parenteral antibiotics, unstable cardiac arrhythmia, active peptic ulcer disease or gastritis, or psychiatric illness/social situations that would limit compliance with study requirement, substantially increase risk of incurring adverse events from Sitravatinib or Nivolumab, or compromise the ability of the subject to give written informed consent.
19. Any condition that, in the opinion of the Investigator, would interfere with evaluation of the study regimen or interpretation of patient safety or study results.
20. Any previous treatment with a PD1 or PD-L1 inhibitor, including Nivolumab.
21. History of another primary malignancy, except for:
    - Malignancy treated with curative intent and with no known active disease ≥3 years before the first dose of study drug and of low potential risk for recurrence,
    - Adequately treated non-melanoma skin cancer without evidence of disease,
    - Adequately treated carcinoma in situ without evidence of disease.
22. Major surgical procedure (as defined by the Investigator) within 28 days prior to the first dose of study medications.
23. Any prior Grade ≥3 immune-related adverse event (irAE) while receiving any previous immunotherapy agent, or any unresolved irAE >Grade 1.

Safety data:

- Overall safety profile as per National Cancer Institute Common Terminology Criteria for Adverse Events (NCI CTCAE) version 5.0.
- Rate of delay of surgery date.

- Rate of disease progression during pre-operative study period.
- Rate and severity of post-surgical complications.

Correlative studies:

- Baseline tumor biopsy to be performed within 10 days prior to the start of Sitravatinib therapy.
- On-treatment tumor biopsy will be performed within 48 hours prior to planned day 15 of Nivolumab. Sitravatinib will not be discontinued before tumor biopsy.
- Pharmacokinetic sample collections and correlatives to be taken at baseline, on the day of on-treatment biopsy and on the day of surgery.
- Surgery will be performed within 23-30 days following the start of study treatment (Day 1).

Assessment of subject evaluability:
A patient will be considered evaluable for correlative study analysis if they fulfill all of the following criteria:

1. Patient tumor sample is available from all 3 tumor sampling time-points, of acceptable quality and quantity for analysis, as assessed by study site correlatives team,
2. Patient has completed at least 11 days of Sitravatinib in the first 2 weeks of therapy and,
3. Patient has completed Nivolumab infusion on Day 15 of study.

Subjects who are not evaluable for correlative will be replaced at the discretion of the Principal Investigator.

1. INTRODUCTION

In addition to the information provided below, please also refer to the Investigator Brochure and any additional data supplied by Mirati Therapeutics.

Squamous Cell Carcinoma of the Oral Cavity (SCCOC).
The incidence of oral cavity cancers in Canada is predicted to increase by 58.9% in the next 17 years[1]. Although the majority of patients presents with early stage disease and undergo curative-intent therapies, prognosis for SCCOC is guarded with a 5-year survival rate of 60%[2]. This survival rate has improved only by approximately 5% over the past 2 decades, highlighting the pressing need for improved treatment options[2].

Receptor Tyrosine Kinase (RTK) inhibitors – Sitravatinib.
Receptor tyrosine kinases (RTKs) are key regulators of signaling pathways leading to cell growth, survival, and migration[3]. Sitravatinib is an orally available receptor tyrosine kinase (RTK) inhibitor that targets multiple closely related receptor tyrosine kinase pathways including VEGFR, PDGFR, c-KIT, c-MET, and the TAM family of receptors (TYRO3, AXL, and MERTK), which are involved in the regulation of cell survival pathways such as tumor growth and invasion, metastatic progression and tumor angiogenesis. These targets are genetically altered and act as oncogenic drivers in multiple cancers including head and neck cancers[4-5]. In head and neck squamous cell carcinoma (HNSCC), c-MET, AXL and VEGFR overexpression is associated with increased cell survival, tumor growth, angiogenesis, and early nodal and systemic metastasis as well as with mechanisms of resistance to radiation and anti-EGFR therapies[6-12]. RTKs inhibitors have still to show clinical activity in HNSCC, but there is evidence based on preclinical tumor models supporting the potential benefit of these agents in this disease[6,13-16].

Additionally, Sitravatinib targets not only act as oncogenic drivers but also play an important role in promoting an immunosuppressive tumor microenvironment (TME). TAM receptors are expressed in macrophages, dentritic cells (DC) and natural killer cells (NK) and downregulate innate immune responses through a negative feedback regulatory mechanism[17-20]; MET suppresses antigen presentation cell (APC) function when activated by its ligand - the hepatic grow factor (HGF) - secreted by the tumor stroma[21]; VEGFR overexpression promotes immune-inhibitory cells, such as regulatory T cells (Tregs), myeloid-derived suppressor cells (MDSCs) and tolerogenic dendritic cells (DCs), and creates an hypoxic microenvironment that precludes recruitment of immune effector cells[22-24]. In this regard, multiple studies using preclinical models have shown how antiangiogenic therapies and TKIs inhibitors targeting VEGF, MET and c-KIT can reverse immune suppression by modulating the tumor microenvironment[25-28]. By blocking MET, VEGFR and TAM family of receptors, Sitravitinib is hypothesized to act as an immunomodulator and enhance the antitumor response by restoring APC function, reducing or eliminating Tregs and MDSCs within the TME and increasing T-cell recruitment.

Immune Checkpoint Inhibitors (ICIs).
Promising response rates, activity across a broad range of histological diagnoses, and early evidence of response durability have resulted in unprecedented interests in the

development of immunotherapeutic agents. One mechanism of cancer immune evasion involves the failure of tumor neo-antigens to activate T cells following engagement of the T cell receptor (TCR). Two-signal hypothesis describes the requirement for concomitant engagement of co-signaling receptors following TCR antigen recognition to determine the path towards cytotoxicity or anergy. HNSCC exploits this protective system by overexpressing co-inhibitory ligands, such as PD-L1[29-32] to induce a relative immunodeficient state in the host[34-38].

Increasing understanding of mutational signatures in HNSCC reveals a landscape that is intrinsically immunogenic[39-40]. Immune checkpoint inhibitors (ICIs) potentiate anti-tumoral immunity by blocking the binding of co-inhibitory molecules to their respective ligands, therefore liberating T cells to exert their cytotoxic effects. Early phase clinical trials of ICIs in recurrent and/or metastatic HNSCC have shown promising results with up to 20% objective response rates, and early evidence of response durability in a proportion of patients[41-46]. Most recently, a phase III clinical trial investigating the anti-PD1 antibody Nivolumab, has shown a 30% reduction in the risk of death compared to single-agent chemotherapy of the Investigator’s choice for patients with platinum-pretreated recurrent or metastatic (R/M) HNSCC leading to its approval by the FDA in this setting[44]. Due to the recent encouraging results, multiple trials are now investigating the activity of ICI’s in first-line R/M disease and in the locally-advanced setting either alone or in combination with other treatments including radiotherapy, chemotherapy, targeted agents and immunotherapies.

1.1 Rationale for conducting this study

1.1.1 Rationale for study design

One of the major criticisms of clinical trials investigating biological therapies involves the validity of clinical tolerability and preliminary activity as primary endpoints in early clinical trials, where, unlike cytotoxic chemotherapeutics, increased doses of biological therapies are not known to be associated with directional improvement in treatment efficacy[47], especially when they are combined based on synergistic hypothesis. There is a growing need to redefine and revolutionize trial endpoints towards evidence of successful molecular targeting, especially when drug combinations are targeting different pathways based on the hypothesis of pharmacodynamics synergy that, theoretically, may be achieved in doses that do not cause higher rates of serious adverse events, therefore potentially limiting unnecessary side effects for patients.

Biomarker-driven window of opportunity studies provide an aperture to evaluate the pharmacodynamic effects of biological therapies in real-time. Resectable squamous cell carcinoma of the oral cavity is uniquely placed where primary tumors are relatively accessible to biopsies, and short treatment and assessment windows will not compromise curative-intent, standard-of-care therapies, while providing a juncture to assess achievement of molecular endpoints. This will significantly contribute to the design of future clinical trials.

1.1.2 Rationale for the combination of Sitravatinib and Nivolumab

TME plays a crucial role in cancer development and progression, as well as in antitumor immune responses[48-49]. Overexpression of c-MET, AXL, VEGFR, and TAM oncogenic pathways promotes an immunosuppressive TME by modulating intratumoral immune-cell population, decreasing T cell recruitment and dampening antigen presentation[17-24], which ultimately lead to ineffective immune responses[49]. Patients without a pre-existing T cell-infiltrated TME are less likely to respond to antiPD-1 checkpoint inhibitors[49-51], therefore representing a mechanism of primary resistance to these agents. A number of studies in HNSCC have shown the prognostic relevance of immune cells and TME as well as their predictive role as a biomarker of response to immune-checkpoint inhibitors[50-53].

Sitravatinib has demonstrated potent, concentration-dependent inhibition of the RTK targets in vitro and in vivo, as well as anti-tumor efficacy over a broad spectrum of human tumor xenograft models including a subset of models exhibiting genetic alterations in RTK targets MET, RET, FLT3 and others. More importantly, studies in syngeneic mouse models showed that the combination of Sitravatinib and anti-PD1 led to dramatic and statistically significant reduction in tumor volume and sustained growth suppression compared to single agent and vehicle controls (Figure 1), suggesting that it can enhance the activity of anti-PD-1 therapy and increase the percentage of patients who derive benefit from checkpoint blockade.

In the phase III trial for R/M HNSCC patients, Nivolumab's overall response rate (ORR) was only 13%, regardless PD-L1 expression[44]. PD-L1 positivity (>1%) was correlated with higher responses and survival, although non-significant[44]. However, other ICIs tested in HNSCC have shown significant higher responses in PD-L1 positive tumors[43,45-46] and, indeed, PD-L1 positive tumors in general tend to demonstrate improved response rates to anti-PD-1/PD-L1 therapies, in comparison to PD-L1 negative tumors[54].

More recently, preliminary data from Checkmate 358, a phase 1/2 study assessing safety and efficacy of neoadjuvant Nivolumab monotherapy in resectable HNSCC in which 16 patients with oral cavity tumors were included, showed that preoperative treatment was overall tolerated without surgery delays and promising tumor reduction rates[55].

We hypothesize that combination therapy with Sitravatinib and Nivolumab in the preoperative setting will achieve synergistic anti-tumor effect through the upregulation of the immune effector cell populations in the TME (increasing immune infiltration, antigen presentation and reducing immunosuppression and Treg) as well as through the blockade of oncogenic molecular and cellular pathways implicated in checkpoint inhibitor resistance and tumor progression, ultimately leading to a more effective anti-tumoral immune response.

1.1.3 Rationale for choice of primary biomarker endpoints

The repertoire of immune checkpoint molecules displayed on tumor cell surfaces at any time point is dynamic and responsive to changes in tissue environment[56]. Tumor immunophenotype, therefore, is fluid. Successful immunosurveillance relies firstly on host immune cell recognition of tumor cells as foreign, followed by the ability of immune effector cells to enter the tumor microenvironment and exert their cytotoxic

effects[57-61]. ICIs act to enhance the latter, denoting a need for pre-existing host immune recognition and intratumoral cell recruitment. Histological analysis of samples from metastatic melanoma patients who received ICI therapy with pembrolizumab revealed a highly significant correlation between the presence of pre-existing tumor infiltrating lymphocytes (TILs) at the invasive tumor margin/rim and treatment response. The presence of TILs in these locations is a theoretical pre-requisite for response to ICIs. Although very few data are available in HNSCC, few studies have demonstrated that the presence of TILs and TME macrophages are prognostic[50], and PD-1/PD-L1 expression in effector T cells might be predictive of response to antiPD-1/PD-L1 inhibitors[51-53].

We hypothesize that Sitravatinib therapy will increase the density of peri-tumoral and intra-tumoral TILS and decrease Tregs and MDSCs, changing PD-L1 expression within TME in the on-treatment biopsy specimen in comparison to baseline. Immune effector cells density within the tumor will be further enhanced in the surgical specimen following combined therapy with Sitravatinib and Nivolumab.

Cytokine production by activated T cells will be used as a marker of activation, and the dynamic change in serum pro-inflammatory cytokines and chemokines with therapy will also be investigated.

1.1.4 Rationale for tumor hypoxia assessment with ^{18}FAZA PET

Tumor hypoxia is well established as a poor prognostic feature in SCCOC[62]. Endogenous hypoxia markers such as HIF-1α, CA-IX, and GLUT1 have been associated with survival for SCCOC in multiple studies[63-69]. There is emerging evidence that tumor hypoxia hampers the endogenous anti-tumor immune response. SCCOCs with intra-tumoral hypoxia may benefit from novel therapeutic approaches that reverse downstream signaling pathways and combat the immunosuppressive effects of hypoxia.

Hypoxic tumors harbor fewer tumor-infiltrating lymphocytes. This may be due in part to reduced vascular access and perfusion; it is not known how well cytotoxic T-lymphocytes (CTLs) penetrate into regions of intra-tumoral hypoxia following PD-L1 blockade. In addition, tumor hypoxia and hypoxia-inducible factor (HIF) signaling impair tumor cell killing by CTLs[70] and raise PD-L1 expression on myeloid-derived suppressor cells (MDSCs), macrophages, dendritic cells, and tumor cells[71-73]. These findings suggest that tumor hypoxia could serve as a predictive biomarker for PD-L1 blockade and that simultaneous targeting of PD-L1 and HIF signaling could provide synergistic anti-tumor effects.

Tumor hypoxia has immunosuppressive effects downstream of HIF signaling. Inhibition of HIF signaling could reverse these immunosuppressive effects and enable greater efficacy of PD-L1 blockade. Although there is no evidence on the effects of Sitravatinib on HIF signaling, it is expected that VEGF blockade should potentially upstream downregulate HIF signaling. Thus, assessment of tumor hypoxia could identify patients most likely to benefit from treatment with Sitravatinib with or without simultaneous PD-L1 blockade.

Detection of tumor hypoxia by positron emission tomography (PET) tracers have shown feasibility for SCCOC[74]. PET imaging of hypoxia is a non-invasive method

that uses hypoxia-sensitive imaging tracers, usually nitroimidazoles, labeled with positron emitters. The nitroimidazole tracer binds in hypoxic regions of the tumor and undergoes radioactive decay, which is detected by PET imaging and spatially localized to form a three-dimensional image of the distribution of hypoxia. ^{18}F-Fluoroazomycin arabinoside (^{18}FAZA) is a newer nitroimidazole that has higher tumor to background contrast ratios than previously used tracers (e.g. ^{18}F-MISO)[75]. ^{18}FAZA-PET is minimally invasive, repeatable over time, and allows evaluation of the entire tumor, thereby overcoming concerns about intra-tumoral heterogeneity and sampling error associated with tumor tissue sampling.

We hypothesize that the combination of Sitravatinib and Nivolumab reduces intra-tumoral hypoxia as determined by pre- and post-treatment ^{18}FAZA-PET, and that the combination of Sitravatinib and Nivolumab results in CTL trafficking into regions of intra-tumoral hypoxia.

1.2 Background for investigational agents

1.2.1 Background information for Sitravatinib

Sitravatinib (MGCD516) is an orally-available, potent small molecule inhibitor of a closely related spectrum of tyrosine kinases including MET, Axl family, VEGFR family, PDGFR family, KIT, FLT3, Trk family, RET, DDR2, and selected Eph family members, which has shown antitumor activity in a variety of in vitro and in vivo model systems.

Preclinical experience with Sitravatinib

Sitravatinib demonstrated potent, concentration-dependent inhibition of the kinase activity of MET, Axl family, VEGFR family, PDGFR family, KIT, FLT3, Trk family, RET, DDR2, and selected Eph family members in vitro cell-based assays. Sitravatinib also inhibited oncogenic functions associated with target RTKs including MET-dependent cell viability and migration and endothelial tube formation and angiogenesis. Consistent with this anti-tumor and anti-angiogenic mechanism of action, Sitravatinib demonstrated also anti-tumor efficacy over a broad spectrum of human tumor xenograft models in vivo, including models exhibiting genetic alterations in RTK targets including MET, RET, FLT3 and others. Sitravatinib also demonstrated inhibition of MET, Axl, RET, and/or EPHA2 activity in selected tumor xenograft models and a PK/PD relationship of plasma concentration of Sitravatinib to anti-tumor activity was established in defining target plasma exposures utilized for projection of target human efficacious plasma levels and dose.

Additionally, Sitravatinib was evaluated in combination with a mouse surrogate anti PD-1 antibody in a syngeneic mouse tumor model (PH-MRTX-004). Treatment with Sitravatinib or an anti-mouse PD-1 antibody resulted in limited growth delay effects whereas the combination led to dramatic and statistically significant reduction in tumor volume and sustained growth suppression compared to single agent and vehicle controls (Figure 1).

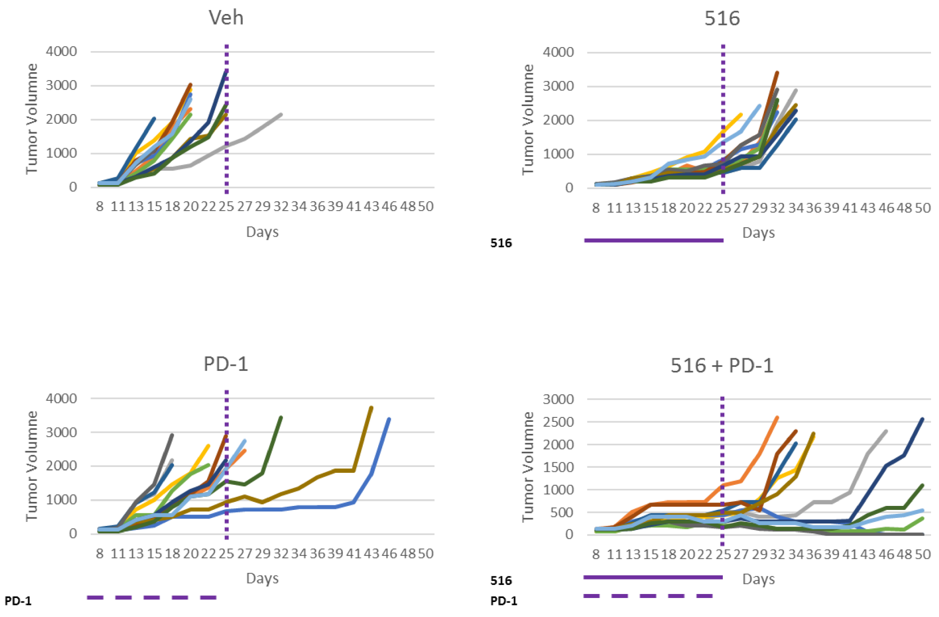

Figure 1. Sitravatinib increases activity of anti-pd1 in syngeneic mouse models: Mice with established CT26 tumors were administered MGCD516 and / or a mouse anti-PD-1 antibody from day eight until day 25 post implant (end of dosing indicated by dotted vertical line). Sitravatinib was dosed orally daily (day 8 to 25), 20 mg/kg; PD-1 antibody was dosed IP, 10 mg/kg (days 8, 11, 15, 18, 22). Decreased tumor volume in the 516 + PD-1 combination cohort was statistically significant compared to vehicle, PD-1 and 516 single agent cohorts by the Student's t-test, p-values < 0.05 from day 22 through the end of the study.

The nonclinical safety profile has been examined in repeated-dose toxicology studies for durations of 7 days and 28 days in rats and dogs. In these repeated-dose toxicology studies increases in C_{max} and AUC_{0-24} values were roughly dose proportional on Day 1 and at the end of study, indicating no accumulation. No consistent significant differences were observed between males and females. In repeat dose toxicity studies in the dog, no target organs were identified, despite over decreases in body weight and food consumption. In the rat, VEGF-related target organs were identified, including the adrenal gland, Brunner's glands in the duodenum, bone and bone marrow, spleen, lymph nodes, thymus, ovary, kidney (glomerulopathy, tubule necrosis, increased basophilic tubules), pancreas, and tongue. All effects, except those in the kidney and pancreas, either recovered or showed partial recovery.

Clinical experience with Sitravatinib

Sitravatinib (MGCD516) has been evaluated as a single agent in the first-in-human Study 516-001 and is currently being evaluated in combination with the PD-1 inhibitor Nivolumab in Study MRTX-500 (NCT02954991).

Study 516-001

Study 516-001 is a multi-center, Phase 1/1b clinical trial characterizing the safety, PK, metabolism, PD and clinical activity of Sitravatinib in patients with advanced solid tumor malignancies. Sitravatinib was administered as a single oral dose for PK profiling, followed by continuous once daily oral administration in cycles of 21 days The dose escalation phase based on PK data and safety assessments proceed using the modified toxicity probability interval (mTPI) method, starting at a dose of 10mg. Dose of 200 mg was demonstrated to exceed the MTD as 4 DLTs were observed among the 4 evaluable patients (intolerable Grade 2 neuropathy, intolerable Grade 2 fatigue, Grade 3 palmar-plantar erythrodysesthesia and intolerable Grade 2 mucositis in 1 patient each). Therefore, the recommended Phase 2 dose (RP2D) for single agent Sitravatinib was initially determined to be 150mg. In the combination setting, 120 mg once daily was selected as the RP2D as this dose level was determined adequate for inhibition of VEG-F and TAM receptors necessary to achieve antitumor efficacy.

As of data cut-off, 26 June 2017 for the Sitravatinib Investigator Brochure, safety data are available on 86 patients (49 men / 37 women; median age 66 years; range 27-85 years) with advanced solid tumor malignancies (18 renal carcinoma, 16 non-small cell lung cancer, 13 prostate cancer, 7 soft tissue sarcoma, 6 colon cancer, 26 other). The most commonly observed AEs reported as related to study drug were fatigue (44%), diarrhea (35%), hypertension (34%), nausea (24%), decreased appetite (22%), vomiting (22%), decreased weight (17%), palmar-plantar erythrodysesthesia (13%) and hypothyroidism (12%). Dose-limiting toxicity (DLT) was reported in four patients who developed palmar-plantar erythrodysesthesia, neuropathy, mucositis, or fatigue. Grade 3 adverse events reported as related to treatment in more than one patient included hypertension (19%); diarrhea (11%); pulmonary embolism, increased lipase, and fatigue (5% each); palmer-plantar erythrodysesthesia, decreased ejection fraction, left ventricular dysfunction, mucosal inflammation, and hyponatraemia (2% each). Grade 4 adverse events reported as related to treatment in 1 patient each (1%) included febrile neutropenia, hypotension and tachycardia. Treatment-related SAEs were reported in 12 patients (14%) and included diarrhea (4%); pulmonary embolism, hypertension and fatigue (2% each); back pain, cardiac failure, ejection fraction decreased, febrile neutropenia, hiccups, hypertension, hypoalbuminaemia, hypokalemia, hypotension, left ventricular dysfunction, odynophagia, oropharyngeal pain, pancreatitis, rash follicular and tachycardia (1% each). Twenty-six deaths were reported in this study, with the primary cause of death being the disease under study (n=17), unknown (n=5), hypoxic respiratory failure, respiratory failure, GI bleed or sepsis (n=1 each).

Study MRTX-500

Study MRTX-500 is a parallel Phase 2 study of Glesatinib, Sitravatinib or Mocetinostat in combination with Nivolumab in advanced or metastatic non-small cell lung cancer patients who have experienced disease progression either on or after

prior treatment with a checkpoint inhibitor therapy (CIT-experienced) or after treatment with platinum-based doublet chemotherapy (CIT-naïve), currently on recruitment. Sitravatinib is administered orally once daily and Nivolumab is administered by intravenous infusion, 240 mg every 15 days in 28-day cycles. Sitravatinib at 120 mg once daily was selected as the RP2D for the combination as no DLTs were observed with the first 6 patients, and this dose level was determined adequate for inhibition of VEG-F and TAM receptors necessary to achieve antitumor efficacy in the combination setting.

As of the data cut-off date (26 June 2017) for the Investigator Brochure, safety data are available for 10 patients. The most commonly observed AEs reported (≥20%) as related to Sitravatinib were; fatigue (50%), diarrhea (40%), aspartate aminotransferase increase (30%), palmar-plantar erythrodysaesthesia (30%), dysphonia (30%), lipase increase (20%), weight decrease (20%), nausea (20%), vomiting (20%), decreased appetite (20%), hyponatremia (20%), hypertension (20%) and hypothyroidism (20%). One patient experienced SAEs of pulmonary embolism and deep-vein thrombosis attributed to Sitravatinib treatment. No immune-mediated AEs have been reported. The study has met the criterion for expansion to Stage 2 of patient enrollment and is ongoing.

Safety data update as of April 13 2018 included 54 patients and confirmed the overall AE profile for the combination remained consistent with what described above. The most commonly observed AEs (>10%) reported related to Sitravatinib were: diarrhea (30%), fatigue (30%), nausea (26%), decreased appetite (24%), vomiting (17%), aspartate aminotransferase increase (15%), alanine aminotransferase increase (15%), dysphonia (15%), hypertension (13%), palmar-plantar erythrodysaesthesia (13%) weight decrease (13%), mucosal inflammation (11%). Grade ≥ 3 Sitravatinib-related AEs were: diarrhea (9%), Fatigue (2%), palmar-plantar erythrodysaesthesia (2%), hypertension (9%), hypertensive crisis (grade 4) (2%), mucosal inflammation (6%), lipase increase (4%), hyponatremia (4%), Syncope (2%), confusional state (2%). 1 patient (2%) died due to a cardiac arrest while on study (grade 5). The Sponsor assessed the Cardiac Arrest as unlikely related to Sitravatinib and not related to Nivolumab because the Investigator stated that the death was due to the subject's disease process and the event occurred 4 days after initiating treatment with Sitravatinib (steady state PK is reached in a mean time of 11 to 15 days).

Non-clinical toxicology studies as well as clinical safety data from the Phase 1/1b and Phase 2 studies suggest that adverse events associated with Sitravatinib are similar to those observed with other small molecule inhibitors of the VEGFR pathway.

For more information regarding Sitravatinib-related toxicity profile and management, please refer to section 14.2.5 and Table 17 of the Appendix.

Potential increased toxicities with the combination

Frequent adverse events, such as fatigue, musculoskeletal pain, decreased appetite, cough, and constipation, which are non-specific and typical of cancer treatment regimens have been observed with Nivolumab and Sitravatinib monotherapy. Potential exists for these AEs to be observed with increased severity or frequency

during use of the combined agents. Management of these effects in patients receiving cancer therapy is well precedented.

More importantly, immune-related AEs (irAEs) of Special Interest based on observed safety events using Nivolumab monotherapy include pneumonitis, colitis, hepatitis, endocrinopathy, nephritis/renal dysfunction, rash, and encephalitis. While Sitravatinib may have immunostimulatory effects, autoimmune adverse effects have not been reported in clinical trials of this investigational study agent, including to date in combination with Nivolumab, nor are they recognized as class effects for this agent. However, the potential for Sitravatinib to exacerbate or promote these adverse events when administered in combination with Nivolumab should be borne in mind. Adverse event incidence data presented below are as reported in the OPDIVO (Nivolumab) Health Canada Product Monograph dated April 2018 and the Sitravatinib Investigator's Brochure dated September 2017. Updates to these data during the conduct of this clinical trial will be found in the current OPDIVO (Nivolumab) Health Canada Product Monographand current Sitravatinib Investigator's Brochure.

A clinically relevant overlap in toxicity may arise between the immune-related colitis attributed to nivolumab and the non-specific, most often mild to moderate diarrhea observed with Sitravatinib. Immune-related colitis has been reported in 2.9% (58/1994) of patients treated with nivolumab, with a median time to onset of 5.3 months (range: 2 days to 20.9 months). Diarrhea has been reported in approximately 35% of patients treated with Sitravatinib, most often beginning within the first month of the start of treatment. The time to onset may be helpful in distinguishing diarrhea that may be attributed to autoimmune effects versus non-specific toxicity.

Tyrosine kinase inhibitors in general, and MET inhibitors in particular, have been associated with non-specific, most often mild to moderate elevation in AST and ALT. Mild to moderate elevations in liver transaminases have also been observed in less than 10% of patient treated with Sitravatinib. The elevations observed with Sitravatinib generally occur within the first cycle of treatment and resolve with interruption of treatment. In patients receiving nivolumab as a single agent, immune-mediated hepatitis occurred in 1.8% (35/1994) of patients; the median time to onset was 3.3 months (range: 6 days to 9 months).

Hypothyroidism has been reported in approximately 12% (10/86) of subjects treated with Sitravatinib.

A clinically relevant overlap in toxicity may arise between the immune-related rash attributed to nivolumab and the non-specific, most often mild (Grade 1) rash observed with Sitravatinib. Rash has been reported in 9% (8/86) of patients treated with Sitravatinib.

Sitravatinib pharmacokinetics and product metabolism

Sitravatinib capsules (10 and 40mg) should be taken with at least 200 mL (1 cup) of water. After single dose administration, Sitravatinib reaches peak concentration in a median time of 3 to 9 hours. Exposure parameters (maximum concentration [C_{max}] and area under the curve [AUC]) are approximately dose proportional with doses up to 200 mg. Mean elimination half-life varies between 40 and 53 hours after oral

administration. The steady state PK is reached in a mean time of 11 to 15 days. Drug accumulation is observed after multiple dose administration and averaged 2.6-fold for C_{max} and 3.1-fold for AUC_{0-24}.

Sitravatinib is metabolized by several cytochromes including CYP 3A4, 2B6, 2D6, and with a low risk of any CYP demonstrating a disproportionate contribution to its metabolism. Although systemic inhibition or induction of the tested CYPs is unlikely at the exposure observed in patients treated with 150 mg QD (RP2D) or lower doses, no formal studies have been performed to assess the potential for drug interactions based on the PK of Sitravatinib. Caution should therefore be used when administering Sitravatinib to patients taking medications that are strong inhibitors or inducers of the cytochrome P450 system. Sitravatinib is an inhibitor of BCRP and P-gp transporters based on in vitro studies, therefore medications that are substrates for BCRP or P-gp transporters should be avoided. The solubility of Sitravatinib is pH dependent. For this reason, medications that are associated with sustained increase in gastric pH may result in decreased exposure to Sitravatinib and should be avoided during treatment when possible.

Potential for Drug-Drug Interactions

Sitravatinib administered in combination with Nivolumab is unlikely to result in clinically relevant drug-drug interactions (DDI) based on absorption, metabolism, elimination or protein binding. Nivolumab is a mAb and is intravenously administered, whereas Sitravatinib is a small molecule therapeutic administered orally; no absorption interactions are expected.

No studies on the metabolism of Nivolumab have been reported in vitro or in humans. Like most therapeutic proteins, Nivolumab is not expected to be metabolized by liver cytochrome P-450 (CYP) or other drug metabolizing enzymes and is unlikely to have an effect on CYPs or other metabolizing enzymes in terms of inhibition or induction.

1.2.2 Background information for Nivolumab

Nivolumab (OPDIVO®) is a human IgG4 kappa immunoglobulin that binds to the PD-1 receptor and blocks its interaction with PD-L1 and PD-L2, releasing PD-1 pathway-mediated inhibition of the immune response, including the anti-tumor immune response. In syngeneic mouse tumor models, blocking PD-1 activity resulted in decreased tumor growth. Background information in addition to that presented below is available in the OPDIVO (Nivolumab) Health Canada Product Monograph.

Preclinical experience with Nivolumab

The non-clinical experience is described in the OPDIVO (Nivolumab) Health Canada Product Monograph.

Clinical experience with Nivolumab

The following includes reprints of selected sections of safety data provided in the OPDIVO (Nivolumab) Health Canada Product Monograph.

Oliva M, et al. J Immunother Cancer 2022; 9:e003476. doi: 10.1136/jitc-2021-003476

A. Safety - Immune-mediated adverse events

Data for the following immune-mediated adverse reactions are based on patients who received Nivolumab monotherapy, or Nivolumab in combination with Ipilimumab in clinical studies across tumor types (melanoma, NSCLC, renal cell carcinoma, SCCHN, classical Hodgkin lymphoma and hepatocarcinoma. Analyses also include safety data from completed studies in other tumor types. Although the rates of immune-mediated adverse reactions were generally similar across tumor types for patients who received Nivolumab monotherapy, hepatic and renal adverse reactions occurred most commonly in renal cell carcinoma (11.3% and 6.9%, respectively); gastrointestinal and skin adverse reactions occurred most commonly in melanoma (17.7% and 38.4%, respectively); pulmonary reactions, specifically pneumonitis occurred most commonly in renal cell carcinoma and NSCLC (3.9% and 3.6%, respectively); and endocrine and gastrointestinal adverse reactions occurred most commonly in SCCHN (11.0% and 14.8%, respectively). For patients receiving Nivolumab in combination with Ipilimumab, there was a higher frequency of liver and thyroid test abnormalities reported in the Nivolumab in combination with Ipilimumab group compared with the monotherapy groups. Grade 3-4 abnormalities in liver were also higher frequency in the Nivolumab in combination with ipilimumab group (19.8%) compared with the monotherapy Nivolumab (5.1%) and monotherapy Ipilimumab (4.5%) groups.

Immune-Mediated Pulmonary Adverse Reactions

Across the clinical trial program, fatal immune-mediated pneumonitis occurred in 5 patients receiving Nivolumab in a dose-finding study at doses of 1 mg/kg (two patients), 3 mg/kg (two patients), and 10 mg/kg (one patient). One patient with Grade 3 pulmonary embolism and Grade 3 pneumonitis subsequently died in the SCCHN clinical trial. In patients treated with Nivolumab monotherapy, the incidence of pneumonitis, including interstitial lung disease, was 3.3% (74/2230). The majority of cases were Grade 1 or 2 in severity reported in 0.8% (17/2230) and 1.8% (40/2230) of patients, respectively. Grade 3 and 4 cases were reported in 0.7% (16/2230) and 0.1% (1/2230) of patients, respectively. No grade 5 cases were reported. The median time to onset was 3.6 months (range: 0.2-19.6). Fifty-two patients received high dose corticosteroids (at least 40 mg prednisone equivalents) for a median duration of 3.5 weeks (range: 0.1-13.1). Fifteen patients (0.7%), fourteen with Grade 3, and one with Grade 4 required permanent discontinuation of Nivolumab. Resolution occurred in 61 patients (82%); with a median time to resolution of 5.6 weeks (0.6-53.1+).

Immune-Mediated Gastrointestinal Adverse Reactions

In patients treated with Nivolumab monotherapy, the incidence of diarrhea or colitis was 13.0% (290/2230). The majority of cases were Grade 1 or 2 in severity reported in 8.9% (198/2230) and 2.8% (62/2230) of patients, respectively. Grade 3 cases were reported in 1.3% (30/2230) of patients. No Grade 4 or 5 cases were reported in these studies. The median time to onset was 1.6 months (range: 0.0-20.9). Thirty-eight patients received high dose corticosteroids (at least 40 mg prednisone equivalents) for a median duration of 2.9 weeks (range: 0.4-40.3). Thirteen patients (0.6%) with Grade 3 diarrhea or colitis required permanent discontinuation of Nivolumab. Resolution

occurred in 259 patients (91%) with a median time to resolution of 2.0 weeks (range: 0.1-88.3+).

Immune-Mediated Hepatic Adverse Reactions

In patients treated with Nivolumab monotherapy, the incidence of liver function test abnormalities was 6.9% (153/2230). The majority of cases were Grade 1 or 2 in severity reported in 3.6% (80/2230) and 1.3% (30/2230) of patients, respectively. Grade 3 and 4 cases were reported in 1.5% (34/2230) and 0.4% (9/2230) of patients, respectively. No Grade 5 cases were reported in these studies. The median time to onset was 2.1 months (range: 0.0-18.7). Thirty-three patients received high dose corticosteroids (at least 40 mg prednisone equivalents) for a median duration of 2.9 weeks (range: 0.1-8.9). Twenty-three patients (1.0%), eighteen with Grade 3 and five with Grade 4 liver function test abnormalities, required permanent discontinuation of Nivolumab. Resolution occurred in 115 patients (76%) with a median time to resolution of 5.1 weeks (range: 0.1-82.6+).

Immune-Mediated Endocrinopathies

In patients treated with Nivolumab monotherapy, the incidence of thyroid disorders, including hypothyroidism or hyperthyroidism, was 9.7% (217/2230). The majority of cases were Grade 1 or 2 in severity reported in 4.0% (90/2230) and 5.2% (115/2230) of patients, respectively. Grade 3 or Grade 4 thyroid disorders were reported in 0.5% (11/2230) and ,1% (1/2230) of patients. Hypophysitis (one Grade 1; one Grade 2, three Grade 3, and one Grade 4), hypopituitarism (four Grade 2 and one Grade 3), adrenal insufficiency (one Grade 1; five Grade 2; and four Grade 3), diabetes mellitus (one Grade 2), and diabetic ketoacidosis (two Grade 3) were reported. No Grade 5 cases were reported in these studies. The median time to onset was 2.8 months (range: 0.3-14.0). Thirteen patients received high-dose corticosteroids (at least 40 mg prednisone equivalents) for a median duration of 1.6 weeks (range 0.1-9.6). Two patients with Grade 3 and one with Grade 4 endocrinopathies required permanent discontinuation of Nivolumab. Resolution of endocrinopathies occurred in 98 patients (45%) with a median time to resolution of 66.6 weeks (0.4-96.1+); + denotes a censored observation.

Immune-Mediated Renal Adverse Reactions

In patients treated with Nivolumab monotherapy, the incidence of nephritis or renal dysfunction was 2.7% (61/2230). The majority of cases were Grade 1 or 2 in severity reported in 1.6% (35/2230) and 0.7% (16/2230) of patients, respectively. Grade 3 and 4 cases were reported in 0.4% (9/2230) and 1% (1/2230) of patients, respectively. No Grade 5 nephritis or renal dysfunction was reported in these studies. The median time to onset was 2.3 months (range: 0.0-18.2). Sixteen patients received high-dose corticosteroids (at least 40 mg prednisone equivalents) for a median duration of 3 weeks (range: 0.1-67.0). Two patients (<0.1%), one with Grade 3 and one with Grade 4 nephritis or renal dysfunction required permanent discontinuation of Nivolumab. Resolution occurred in 37 patients (63%) with a median time to resolution of 11.1 weeks (range: 0.1-77.1+).

Immune-Mediated Skin Adverse Reactions

In patients treated with Nivolumab monotherapy, the incidence of rash was 26.0% (579/2230). The majority of cases were Grade 1 in severity reported in 20.2% (451/2230) of patients. Grade 2 and Grade 3 cases were reported in 4.8% (107/2230) and 0.9% (21/2230) of patients, respectively. No Grade 4 or 5 cases were reported in these studies. Median time to onset was 1.4 months (range: 0.0-17.3). Twenty-one patients received high dose corticosteroids (at least 40 mg prednisone equivalents) for a median duration of 2.1 weeks (range: 0.1-38.7). Three patients (0.1%) with Grade 3 rash required permanent discontinuation of Nivolumab. Resolution occurred in 361 patients (63%) with a median time to resolution of 16.0 weeks (0.1-113.7+).

Immune-Mediated Encephalitis

Nivolumab can cause immune-mediated encephalitis with no clear alternate etiology. This has been observed in less than 1% of patients (0.2%, 3/1994) treated with Nivolumab monotherapy in clinical trials across doses and tumor types, including one fatal case of limbic encephalitis. Evaluation of patients with neurologic symptoms may include, but not be limited to, consultation with a neurologist, brain MRI, and lumbar puncture. Withhold Nivolumab in patients with new-onset moderate to severe neurologic signs or symptoms and evaluate to rule out infectious or other causes of moderate to severe neurologic deterioration.

Other Immune-Mediated Adverse Reactions

Nivolumab can cause other clinically significant immune-mediated adverse reactions. These have been observed with Nivolumab treatment. Across clinical trials of Nivolumab and Nivolumab in combination with Ipilimumab investigating various doses and tumor types, the following immune-mediated adverse reactions were reported in less than 1% of patients: uveitis, Guillain Barré syndrome, pancreatitis, autoimmune neuropathy (including facial and abducens nerve paresis), demyelination, myasthenic syndrome, gastritis, sarcoidosis, duodenitis, myositis, myocarditis, and rhabdomyolysis. Cases of Vogt-Koyanagi-Harada syndrome have been reported during post approval use of Nivolumab or Nivolumab in combination with Ipilimumab.

Infusion Reactions

Nivolumab can cause severe infusion reactions. These have been reported in clinical trials of Nivolumab and Nivolumab in combination with Ipilimumab. Severe infusion reactions have been reported in less than 1.0% of patients in clinical trials of Nivolumab.

Carcinogenesis and Mutagenesis

The mutagenic and carcinogenic potential of Nivolumab have not been evaluated. Fertility studies have not been performed with Nivolumab.

B. Overall Safety and antitumor activity of Nivolumab in HNSCC

The safety of Nivolumab was evaluated in a randomized, open-label, Phase 3 trial (Study CHECKMATE-141) in patients with recurrent or metastatic SCCHN and progression during or after one prior platinum-based therapy. Patients received 3

mg/kg of Nivolumab (n=236) administered intravenously over 60 minutes every 2 weeks or investigator's choice of either cetuximab (n=13), 400 mg/m2 loading dose followed by 250 mg/m2 weekly, or methotrexate (n=46) 40 to 60 mg/m2 weekly, or docetaxel (n=52) 30 to 40 mg/m2 weekly (see CLINICAL TRIALS). The median duration of therapy was 1.9 months (range: 0.03-16.1+ months) in Nivolumab-treated patients and was 1.9 months (range: 0.03-9.1 months) in patients receiving investigator's choice. In this trial, 18% of patients received Nivolumab for greater than 6 months and 2.5% of patients received Nivolumab for greater than 1 year. In Study CHECKMATE-141, therapy was discontinued for adverse reactions in 4% of patients receiving Nivolumab and in 10% of patients receiving investigator's choice. Twenty-four percent (24%) of Nivolumab-treated patients had a drug delay for an adverse reaction. Serious adverse reactions occurred in 7% of Nivolumab-treated patients and in 15% receiving investigator's choice. There were two treatment-related deaths associated with Nivolumab (pneumonitis and hypercalcemia) versus none in patients treated with investigator's choice therapy.

Nivolumab pharmacokinetics

The pharmacokinetics (PK) of Nivolumab is linear in the dose range of 0.1 to 20 mg/kg. The geometric mean clearance (CL), volume of distribution at steady state (Vss), and terminal half-life (t1/2) of Nivolumab were 9.5 mL/h, 8.0 L and 26.7 days, respectively. Nivolumab CL increased with increasing body weight. Dosing normalized to body weight produced approximately uniform steady-state trough concentrations over a wide range of body weights. The metabolic pathway of Nivolumab has not been characterized. As a fully human IgG4 monoclonal antibody, Nivolumab is expected to be degraded into small peptides and amino acids via catabolic pathways in the same manner as endogenous IgG.

Special Populations and Conditions

A population PK analysis based on data from 909 patients suggested the effects of age, gender, race, tumor type, tumor size, hepatic impairment and eGFR on Nivolumab clearance are not clinically relevant (point estimate and 95% CI within 80-120%). ECOG status and body weight had a modest effect on Nivolumab clearance (upper limit of 95% CI less than 30%). These effects are unlikely to be clinically relevant, given the flat exposure-response relationships for both efficacy and safety.

Hepatic Insufficiency

The effect of hepatic impairment on the CL of Nivolumab was evaluated in patients with mild hepatic impairment (TB 1.0 to 1.5 times ULN or AST >ULN as defined using the National Cancer Institute criteria of hepatic dysfunction; n=92) compared to patients with normal hepatic function (TB and AST ≤ULN; n=804) in the population PK analyses. No clinically important differences in the CL of Nivolumab were found between patients with mild hepatic impairment and normal hepatic function. Nivolumab has not been studied in patients with moderate (TB >1.5 to 3 times ULN and any AST) or severe hepatic impairment (TB >3 times ULN and any AST).

Renal Insufficiency

No dedicated clinical studies were conducted to evaluate the effect of renal impairment on the PK of Nivolumab. In population PK analyses, the effect of renal impairment on the CL of Nivolumab was evaluated in patients with mild (GFR <90 and ≥60 mL/min/1.73 m2; n=379), moderate (GFR <60 and ≥30 mL/min/1.73 m2; n=179), or severe (GFR <30 and ≥15 mL/min/1.73 m2; n=2) renal impairment compared to patients with normal renal function (GFR ≥90 mL/min/1.73 m2; n=342). No clinically important differences in the CL of nivolumab were found between patients with mild or moderate renal impairment and patients with normal renal function. Data are not sufficient for drawing a conclusion on patients with severe renal impairment.

Nivolumab 30-minute duration rational

Nivolumab is infused over 30 minutes as per institutional guidelines based on data from Bristol-Myers Squibb (BMS) showing comparable patient safety profiles when Nivolumab is infused over 30 minutes compared to 60 minutes.

2. STUDY OBJECTIVES

2.1 Primary objective

To evaluate the pharmacodynamic and immune effects of pre-operative therapy with Sitravatinib in combination with Nivolumab in patients with squamous cell carcinoma of the oral cavity.

2.2 Secondary objectives

1. To evaluate the safety and tolerability of pre-operative therapy with Sitravatinib and Nivolumab in patients with squamous cell carcinoma of the oral cavity.
2. To evaluate the pre-operative clinical activity of the combination of Sitravatinib and Nivolumab in patients with squamous cell carcinoma of the oral cavity.
3. To determine Sitravatinib plasma levels alone and in combination with Nivolumab.

2.3 Exploratory objectives

1. To evaluate the dynamic changes in immune cell activation and/or suppression using flow cytometry, DNA/RNA sequencing, and FACS sorting.
2. To evaluate the dynamic changes in intratumoral hypoxia with pre-operative Sitravatinib and Nivolumab therapy using ^{18}FAZA PET.

2.4 Study endpoints

Objectives and related endpoints are described in Table 1 below.

Objectives	Endpoints	Analysis
**Primary**		
To evaluate the pharmacodynamic and immune effects of pre-operative therapy with Sitravatinib and Nivolumab in patients with SCCOC	• Tumor PD-L1 expression by IHC<br>• Density of immune cell population in the tumor and/or peripheral blood, including circulating tumor DNA (ctDNA)*, T-cell subsets, NK cells and myeloid-derived cell subsets.<br>• Serum pro-inflammatory cytokines and chemokines<br><br>*Applicable to peripheral blood as described in Table 10	• Section 8.2
**Secondary**		
To evaluate the safety and tolerability of pre-operative therapy with Sitravatinib and Nivolumab on patients with SCCOC	• Toxicities as per NCI CTCAE v5.0<br>• Rate of completion of surgery within the initially planned window<br>• Rate of post-operative complications	• Section 8.2
To evaluate the pre-operative clinical activity of the combination of Sitravatinib and Nivolumab in patients with SCCOC	• Rate of disease progression as per RECIST v1.1 during the pre-operative treatment period<br>• Pathologic treatment effect in tumor and/ or lymph nodes<br>• Rate of nodal extracapsular extension and positive margins	• Sections 8.3
To determine Sitravatinib plasma levels alone and in combination with Nivolumab.	• Analysis of plasma Sitravatinib concentration before and after Nivolumab therapy	• Section 8.1
**Exploratory**		
To evaluate the dynamic changes in immune cell activation and/or suppression using flow cytometry, DNA/RNA sequencing, and FACS sorting	• Additional tumor cell surface marker expression<br>• Flow cytometry analysis of activation markers on circulating immune cells<br>• Tumor and immune cell genome and transcriptome analysis	• Section 8.2

To evaluate the dynamic changes in intratumoral hypoxia with pre-operative Sitravatinib and Nivolumab therapy	• Analysis of tumor ^{18}FAZA-PET uptake patterns	• Section 8.4

Table 1. Objectives and related endpoints

3. STUDY PLAN AND PROCEDURES

3.1 Overall study design

This is a single center, open-label, non-randomized, pre-operative window of opportunity study for patients with resectable squamous cell carcinoma of the oral cavity who are considered suitable for curative-intent surgical resection, with pre-operative Sitravatinib and Nivolumab.

Up to 15 patients with a clinical diagnosis of resectable squamous cell carcinoma of the oral cavity will be enrolled into the study in order to identify a total of 12 evaluable patients for the primary endpoint of determining the pharmacodynamic and immune effects of the combination (see evaluability in Section 3.2.3).

Patients will be treated pre-operatively with Sitravatinib, which will be administered orally at a dose of 120mg once daily on a continuous basis until 48 hours before planned surgery, or for a maximum period of 28 days.

Nivolumab will be given as a single intravenous infusion of 240mg over 30 minutes of duration, on Day 15 of the study.

Surgery will be performed within 23-30 days following the start of study treatment (Day 1) (see Figure 2 for study schema).

3.2 Statistical design

3.2.1 Determination of sample size

This is a proof-of-concept study, with no specific statistical assumptions at trial onset. We aim to recruit 12 patients who are evaluable for both pharmacodynamics and correlative studies, per the criteria defined in Section 3.2.3. Assuming a 20% unevaluable rate, an estimated total of 15 patients will be recruited.

3.2.2 Planned analyses

This is a single stage study with all analyses to be conducted at the end of the study data lock. No interim analysis is planned.

Pharmacodynamic and immune effects of pre-operative therapy with Sitravatinib and Nivolumab on patients with squamous cell carcinoma of the oral cavity will be summarized for the evaluable population as defined in Section 3.2.3.
Pharmacokinetic concentration data will be listed for each patient, and a summary will be provided for the evaluable population as defined in Section 3.2.3

Safety and tolerability data will be summarized for all enrolled patients who have received at least 1 dose of either Sitravatinib or Nivolumab.

3.2.3 Evaluability

A patient will be considered evaluable for correlative study analysis if they fulfill all of the following criteria:

1. Patient tumor sample is available from all 3 tumor sampling time-points, of acceptable quality and quantity for analysis, as assessed by study site correlatives team,
2. Patient has completed at least 11 days of Sitravatinib in the first 2 weeks of therapy and,
3. Patient has completed Nivolumab infusion on Day 15 of study.

Subjects who are not evaluable for correlative will be replaced at the discretion of the Principal Investigator.

3.3 Study periods

3.3.1 Screening period

The screening period will begin once the patient has signed the study informed consent. Patients will be evaluated against study inclusion and exclusion criteria as outlined in sections 4.1 and 4.2. Newly obtained tumor biopsy sample will be required.

All screening assessments must be performed $\leq$ 10 days to first dose of Sitravatinib. The exception is CT or MRI scans, which should be done as standard of care within clinically acceptable windows. Assessment of left ventricular function by MUGA is to be performed within 4 weeks prior to the first dose of Sitravatinib.

3.3.2 Treatment period

The treatment period will begin on the first dosing day of Sitravatinib. Study treatment, assessment and correlative sample collections are as outlined in Table 7. Pre-surgery assessments are to be performed within 7 days prior to day of surgery.

3.3.3 Follow-up period

Four-to-eight weeks after completion of surgery, patients will be reviewed for safety evaluation. Subsequent follow-up for disease progression will continue by telephone or review of patient medical records for up to 2 years after the completion of surgery.

3.4 End of study definition

The end of study will be 2 years after the last-enrolled patient's day of surgery.

3.5 Early study termination

This study can be terminated at any time for any reason by the Sponsor and Mirati Therapeutics. Should this be necessary, active patients should be seen as soon as

possible for End of Treatment (EoT) visit and the assessments for EoT should be performed as described in Table 7 for study follow-up visit after surgery. The Investigator will be responsible for informing the REB and/or other Regulatory Authorities of the early termination of the trial, as well as to ensure that adequate consideration is given to the protection of the patient's interests.

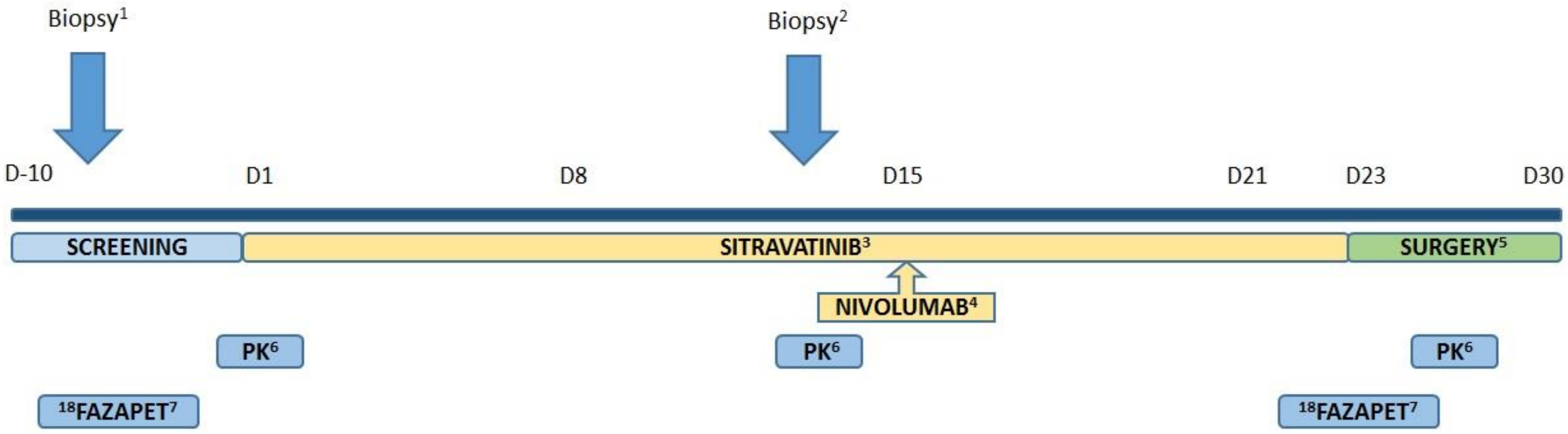

Figure 2. Study Schema.

1. Newly-obtained tumor biopsy at baseline to be performed ≤ 10 days prior to the start of Sitravatinib therapy.
2. On-treatment tumor biopsy will be performed within 48 hours prior to the planned Day 15 Nivolumab therapy.
3. Patients will start therapy with Sitravatinib within 10 days of study enrolment at the RPH2D 120mg once daily on a continuous basis until 48h before surgery and for maximum period of 28 days.
4. Nivolumab will be given as a single infusion at a dose of 240 mg over a period of 30-minutes, on Day 15 of the study.
5. Surgery will be performed within 23 to 30 days following the start of study treatment, with at least a 48-hour period after the last dose of Sitravatinib.
6. Pharmacokinetic sample collections are to be performed on a single time-point at baseline, on the day of on-treatment biopsy and on the day of surgery.
7. ^{18}FAZA PET testing is optional, however is strongly encouraged to be performed within screening and pre-surgery assessment periods.

4. SUBJECT SELECTION CRITERIA

The following eligibility criteria are designed to select patients for whom protocol treatment is considered appropriate. Patients will be recruited from a population of patients with oral cavity cancers referred to the University Health Network and Princess Margaret Cancer Centre, for whom surgical resection of the primary tumor is indicated as standard treatment. All relevant medical and non-medical conditions should be considered when deciding whether this protocol is suitable for a particular patient.

4.1 Inclusion criteria

For inclusion in the study subjects should fulfill the following criteria:

Informed consent

1. Signed written and voluntary informed consent.
2. Patient must be willing and able to comply with scheduled visits, treatment plan, laboratory tests and other study procedures.
3. Age ≥ 18 years, male or female.

Disease characteristics

4. Patient must be diagnosed with histologically confirmed squamous cell carcinoma of the oral cavity (SCCOC) (floor of mouth, anterior 2/3 tongue, buccal mucosa, upper and lower gingiva, retromolar trigone and hard palate) previously untreated, considered resectable by the head and neck treating surgeon (T2-4a, N0-2, or T1 - greater than 1 cm - N2, M0; without evidence of distant metastasis).
5. Patient must be willing and able to provide 2 fresh tumor biopsies for histopathological and biomarker evaluation: one at baseline and one after treatment with Sitravatinib but prior to treatment with Nivolumab. Archival tissue sample will be requested if available.
6. No anti-neoplastic treatment is allowed between the time from obtaining baseline tumor specimen and enrollment.

Patient characteristics

7. ECOG performance status 0-1.
8. Patient must have adequate organ function as determined by the following:
    - Renal function:
        i. Serum creatinine ≤ 1.5 ULN (upper limit of normal range) or a calculated creatinine clearance of ≥ 50mL/min using the following formula:

Creatinine clearance = [(140-age) x wt (kg) x Constant*] / creatinine (umol/L)

*Constant = 1.23 for men, and 1.04 for women

- Bone marrow function (without hematopoietic growth factors or transfusion):
  i. Absolute neutrophil count (ANC) $\geq$ 1.0 x 10^9/L
  ii. Leukocytes $\geq$ 2.0 x 10^9/L
  iii. Hemoglobin $\geq$ 90 g/L or $\geq$ 9g/dL
  iv. Platelets $\geq$ 100 x 10^9/L
- Liver function:
  i. Total bilirubin ≤ 1.5 x ULN or ≤ 3 x ULN for patients with Gilbert Syndrome.
  ii. Aspartate aminotransferase (AST/SGOT) and alanine aminotransferase (ALT/SGPT) $\leq$ 2.5 x ULN
- Cardiac function:
  i. A normal left ventricular ejection fraction (LVEF) of ≥50% by a MUGA scan performed within 4 weeks of the study commencement.

9. Evidence of post-menopausal status, or negative urinary or serum pregnancy test for female pre-menopausal patients. Women will be considered post-menopausal if they have been amenorrheic for 12 months without an alternative medical cause. The following age-specific requirements apply:
   - Women <50 years of age would be considered post-menopausal if they have been amenorrheic for 12 months or more following cessation of exogenous hormonal treatments and if they have luteinizing hormone and follicle-stimulating hormone levels in the post-menopausal range for the institution or underwent surgical sterilization (bilateral oophorectomy, bilateral salpingectomy or hysterectomy).
   - Women ≥50 years of age would be considered post-menopausal if they have been amenorrheic for 12 months or more following cessation of all exogenous hormonal treatments, had radiation-induced menopause with last menses >1 year ago, had chemotherapy-induced menopause with last menses >1 year ago, or underwent surgical sterilization (bilateral oophorectomy, bilateral salpingectomy or hysterectomy). Women of child-bearing potential (WOCBP) or men whose partner is a WOCBP agrees to use contraception while participating in this study, and for a period of 6 months following termination of study treatment.
10. Subject is willing and able to comply with the protocol for the duration of the study including undergoing treatment and scheduled visits and examinations including follow up.

4.2 Exclusion criteria

Subjects should not enter the study if any of the following exclusion criteria are fulfilled:

1. Primary site of head and neck carcinoma unknown, lip, skin, or outside the oral cavity.
   - Patients with tumors that invade major vessels or are within ≤ 3 mm of the carotid artery as shown unequivocally by imaging studies.

2. Patients with any prior history of clinically significant bleeding related to the current head and neck cancer.
3. Patients with a history of gross hemoptysis (bright red blood of ½ teaspoon or more per episode of coughing) ≤ 3 months prior to enrollment.
4. Prior or concurrent radiation therapy to tumor at site of planned resection.
5. Any concurrent chemotherapy, biologic, immunologic or hormonal therapy for cancer treatment.
    - Concurrent use of hormones for non-cancer-related conditions (eg, insulin for diabetes and hormone replacement therapy) is acceptable.
6. Current or prior use of immunosuppressive medication within 14 days prior to starting dosing. The following are <u>exceptions</u> to this criteria:
    - Intranasal, inhaled, topical steroids, or local steroid injections (eg, intra-articular injection).
    - Adrenal replacement steroid > 10 mg daily prednisone equivalent are permitted in the absence of active autoimmune disease.
    - Steroids as premedication for hypersensitivity reactions (eg, computed tomography scan premedication).
7. Active or documented history of autoimmune disease within 2 years before screening, including:
    - Active or prior documented inflammatory bowel disease (eg. Crohn's disease, ulcerative colitis).
    - Patients with vitiligo, resolved childhood asthma/atopy, type I diabetes mellitus, Grave's disease, Hashimoto's disease, or psoriasis not requiring systemic steroids and/or immunosuppressive agents within the past 2 years, are <u>not</u> excluded.
8. History of primary immune deficiency.
9. History of stroke or transient ischemic attack within the previous 6 months.
10. History of uncontrolled hypertension (> 150 mm Hg systolic or > 100 mm Hg diastolic) on multiple observations despite standard of care treatment.
11. Any of the following cardiac abnormalities:
    - Unstable angina pectoris,
    - Previous history of congestive heart failure of any NYHA Class,
    - QTc >480 milliseconds,
    - Left ventricular ejection fraction (LVEF) < 50.
12. Concomitant medication known to cause prolonged QT that cannot be discontinued or changed to a different medication prior to enrollment.
13. History of organ transplant that requires use of immunosuppressive medications.
14. Known allergy or reaction to any components of Sitravatinib and/or Nivolumab formulation.
15. Subjects who are known to be human immunodeficiency (HIV) positive.
16. Has a known history of or is positive for active hepatitis B (defined as hepatitis B surface antigen [HBsAg] reactive) or hepatitis C (defined as HCV RNA [qualitative] is detected).
    - HBV DNA must be undetectable and HBsAg negative at Screening

Visit.
- Participants who have had definitive treatment for HCV are permitted if HCV RNA is undetectable at Screening Visit.

17. Female patients who are pregnant or breast-feeding.
18. Uncontrolled intercurrent illness including, but not limited to, ongoing or active clinically significant infection requiring parenteral antibiotics, unstable cardiac arrhythmia, active peptic ulcer disease or gastritis, or psychiatric illness/social situations that would limit compliance with study requirement, substantially increase risk of incurring adverse events from Sitravatinib or Nivolumab, or compromise the ability of the subject to give written informed consent.
19. Any condition that, in the opinion of the Investigator, would interfere with evaluation of the study regimen or interpretation of patient safety or study results.
20. Any previous treatment with a PD1 or PD-L1 inhibitor, including Nivolumab.
21. History of another primary malignancy, <u>except for</u>:
    - Malignancy treated with curative intent and with no known active disease ≥3 years before the first dose of study drug and of low potential risk for recurrence,
    - Adequately treated non-melanoma skin cancer without evidence of disease,
    - Adequately treated carcinoma in situ without evidence of disease.
22. Major surgical procedure (as defined by the Investigator) within 28 days prior to the first dose of study medications.
23. Any prior Grade ≥3 immune-related adverse event (irAE) while receiving any previous immunotherapy agent, or any unresolved irAE >Grade 1.

5. STUDY CONDUCT

5.1 Subject enrollment

Prior to registering a patient, the site must have submitted all necessary regulatory documentation to the Tumor Immunotherapy Program Central Office. The eligibility checklist will only be sent once this has been received.

All patients will be screened by one of the investigators prior to entry on this study. An explanation of the study and discussion of the expected side effects and presentation of the informed consent document will take place.

No patient can receive protocol treatment until registration with the Central Office as taken place. All eligibility criteria must be met at the time of registration. There will be no exceptions. Any questions should be addressed with the Central Office prior to registration.

To register a patient, the following documents are to be completed by the research nurse or data manager and emailed to the TIP Central Office:

- Signed patient consent form
- Eligibility Checklist signed by the investigator

To complete the registration process, central office will review the checklist and once eligibility has been confirmed:

- Assign a patient serial number
- Confirm the patient dose
- Register the patient on the study
- Fax or e-mail the confirmation worksheet with the patient serial number and dose to the participating site

To ensure immediate attention is given to the emailed checklist, the site is advised to also call the Central office study coordinator. Patient registrations will be accepted between the hours of 9am to 5pm Monday to Friday, excluding Canadian statutory holidays when the central office will be closed.

All eligible patients enrolled in the study will be entered in a patient registration log maintained by the Tumor Immunotherapy Program central office. Following registration, patients should begin protocol treatment within 72 hours.

No randomization will be performed in this study.

5.2 Patient numbering

Each patient is identified in the study by a Subject Number (SN) that is assigned when the patient signs informed consent. The subject number is retained as the primary identifier for the patient throughout his/her entire participation in the trial. The SN consists of the trial number with a sequential patient number suffixed to it, so that each subject is numbered uniquely across the entire database. Upon signing registration, the patient is assigned to the next sequential SN available to the Investigator.

5.3 Treatments

5.3.1 Identity of investigational product(s)

For this study, the investigational drugs refer to Sitravatinib and Nivolumab. Sitravatinib will be provided by Mirati Therapeutics. Commercial supply of Nivolumab will be used and reimbursed by Mirati Therapeutics. Nivolumab will be packaged as per commercial standards. Labels containing Health Canada requirements will be affixed to packaging containing the following information in English and French:
Protocol Number: SNOW-001
Investigational Drug: to be used by qualified investigators only.
Sponsor: Tumor Immunotherapy Program
Princess Margaret Cancer Centre
700 University Avenue, 7-624
Toronto, ON Canada M5G 1Z5

All dosages prescribed and dispensed to patients and all dose changes during the study must be recorded in the Dosage Administration Record eCRF.

Study Treatment	Manufacturer	Pharmaceutical form and route of administration	Dose	Frequency and/or Regimen
Sitravatinib	Mirati	Oral capsule	120mg	Once daily
Nivolumab	BMS	Solution for intravenous infusion	240mg	Once

Table 2. Dose and treatment schedule.

5.3.2 Doses and treatment regimens

Sitravatinib is to be given orally once a day as scheduled. Nivolumab is to be given on Day 15, as a single infusion at a dose of 240mg, over 30-minute duration. Table 3 describes dose and dose reduction of Sitravatinib. On day 15, when Sitravatinib and Nivolumab dosing are both scheduled, Nivolumab can be administered any time in relation to Sitravatinib.

5.3.3 Treatment duration

Sitravatanib is only administered for a maximum of 28 doses (once a day until 48 hours before scheduled surgery, or for a maximum period of 28 days) during the first 4 weeks, and Nivolumab is only given once on Day 15 (during week 3). There is no additional dosing of study drugs. Surgery will be performed within approximately 23 to 30 days following the start of study treatment. There is no dosing of study drugs post-surgery.

A patient will be discontinued from study if they do not meet the evaluability criteria for correlative study, as defined in Section 3.2.3.

A patient may discontinue treatment in the event of unacceptable toxicity, clinical or radiological disease progression as per RECIST 1.1, and/or withdrawal of consent.

5.3.4 Ancillary treatments

Patients should not receive any pre-medications before the first Sitravatinib dose and/or Nivolumab dose. If a patient experienced a suspected allergic reaction, he/she may receive pre-medications according to the Investigator's discretion and local institutional guidelines for subsequent doses. Guidelines for the management of infusion-related reactions are outlined in Section 14.2.3. The use of corticosteroid-based pre-medication will be approved on a case-by-case basis by the Principal Investigator.

Acute allergic reactions should be treated according to institutional guidelines. In the event of anaphylactic/anaphylactoid reactions, any therapy necessary to restore normal cardiopulmonary status should be implemented immediately. These events

should be designated reportable as a SAE regardless of whether the patient requires hospitalization. Please refer to SAE reporting section for details.

5.3.5 Dose modifications and delays

5.3.5.1 Sitravatinib dose modifications and delays

For patients who do not tolerate protocol-specified dosing schedule, dose adjustments are permitted in order to allow the patient to continue the study treatment. In the event of adverse events attributed to Sitravatinib and deemed intolerable by the Investigator, treatment should be either temporarily or permanently discontinued. For patients who temporarily discontinue treatment, treatment may resume following resolution of treatment-related adverse events to Grade 1 or baseline with the administration of Sitravatinib at a reduced dose level as outlined in Table 3. If treatment-related adverse events do not resolve to grade 1 or baseline in within 5 days, treatment will be permanently discontinued. Once the dose has been reduced, re-escalation is generally not recommended but may be considered on a case-by-case basis. If the administration of Sitravatinib is interrupted for reasons other than toxicity, then treatment with the study drug may be resumed at the same dose.

	**Dose level 0**	**Dose level -1**	**Dose level -2**
**Sitravatinib**	120 mg once daily	80 mg once daily	60 mg once daily

Table 3. Sitravatinib Sequential Dose Reductions for Individual Patients

Dose reduction below 60 mg once daily (QD) may be undertaken after discussion with the Principal Investigator. If treatment with Sitravatinib is withheld for ≥ 5 consecutive days, then permanent discontinuation from this study drug should be considered.

5.3.5.2 Sitravatinib Adverse Event Management Guidelines

The following are guidelines for management of potential adverse events related to treatment with Sitravatinib or agents in the same class of cancer treatment.

General Management of Non-haematological Toxicities

Non-haematological toxicities ≥ Grade 3 and considered to be related to Sitravatinib treatment should be managed with Sitravatinib interruption, with or without dose reduction, until resolution of toxicity to ≤ Grade 1 or to baseline value. If the toxicity is adequately managed by routine supportive care (such as anti-emetics, anti-diarrheals, or electrolyte supplementation), Grade 3 fatigue, or amylase or lipase elevation, treatment may be resumed at the same dose; if not, treatment may be resumed at a reduced dose as outlined in **Error! Reference source not found.**. Recurrence of the toxicity may be managed similarly. If treatment is interrupted for ≥ 5 days, permanent discontinuation from study treatment should be considered.

Toxicity[1,2]	Treatment Delay	Dose Reduction
Grade 1 or 2	May be implemented based on Investigator discretion	
Grade 3 or 4	Hold until ≤ Grade 1 or return to baseline	Resume at dose level -1 or levels below that inducing the toxicity. Exceptions presented in footnotes[3]

Table 4: General Management of Sitravatinib Non-haematological Toxicities

1. Special cases for Sitravatinib specific adverse events are presented in Section 5.3.5.3.
2. The current OPDIVO (Nivolumab) Health Canada Product Monograph must be consulted to determine appropriate causality and use the toxicity management guidelines for Nivolumab accordingly.
3. Patients may resume at the same dose in the following cases: grade 3 nausea, vomiting or diarrhea that is treated with supportive care and persists for ≤ 72 hours; grade 3 or 4 electrolyte abnormality that is not clinically complicated and resolves spontaneously or with conventional medical treatment within 72 hours; grade 3 fatigue that persists for ≤ 5 days; grade 3 or 4 amylase or lipase elevation that is not associated with symptoms or clinical manifestations of pancreatitis.

General Management of Haematological Toxicities

Haematological toxicities are not a frequent cause of treatment interruption or discontinuation of Sitravatinib treatment. Observed ≥ Grade 3 haematological events that are considered to be causally related to Sitravatinib should initially be managed using treatment interruption. In addition, dose reduction of Sitravatinib should be implemented in the following cases:

- Grade 3 or 4 febrile neutropenia;
- Grade 4 neutropenia persisting for ≥ 5 days; or
- Grade 4 thrombocytopenia of any duration or Grade 3 thrombocytopenia with bleeding.

5.3.5.3 Sitravatinib Specific Adverse Event Management Guidelines

The following are guidelines for management of potential adverse events more specific to treatment with Sitravatinib or agents in the same class of cancer treatment.

Hypertension

Hypertension, including Grade 3 events, has been reported with Sitravatinib. Dihydropyridine calcium channel blockers such as nifedipine, amlodipine, and nicardipine may be considered if anti-hypertensive therapy is required and should be considered for patients with Grade 3 hypertension without clinically significant increases in blood pressure (see

). On the other hand, in cases of Grade 3 hypertension with clinically significant increases in blood pressure (see

), temporary suspension of Sitravatinib dosing is recommended until blood pressure is controlled. Treatment with Sitravatinib may resume at the same or a lower dose at the discretion of the Investigator. If significant hypertension recurs, options include change in medical management of the patient, reduction of Sitravatinib dose, or

discontinuation of study treatment, at the discretion of the Investigator. In the event of Grade 4 hypertension, Sitravatinib should be permanently discontinued (see

Hypertension	Treatment Interruption	Dose Reduction
Grade 1 or 2	May be implemented based on Investigator discretion as per Table 4.	
Grade 3 without clinically significant increases in blood pressure as defined below	May be implemented based on Investigator discretion as per Table 4. Consider introducing antihypertensive medication.	
Grade 3 with clinically significant increases in blood pressure *defined as* either an increase of ≥ 30 mmHg in systolic blood pressure to ≥ 180 mmHg *or* increase of ≥ 20 mmHg in diastolic blood pressure to ≥ 110 mmHg, confirmed with repeated testing after at least 5 minutes	Hold until ≤ Grade 2 or return to baseline	May be implemented based on Investigator discretion
Grade 4	Discontinue Sitravatinib	Discontinue Sitravatinib

).

Table 4: Sitravatinib Dose Modification for Hypertension.

<u>Palmar-Plantar Erythrodysesthesia (PPE)</u>

Palmar plantar erythrodysesthesia (PPE) has been reported as a dose-limiting toxicity in the Phase 1 study of Sitravatinib. Measures that can be taken to manage PPE include avoidance of exposure of hands and feet to hot water when washing dishes or bathing, or to other sources of heat, avoidance of activities that cause unnecessary force or friction (rubbing) on the hands or feet, avoiding contact with harsh chemicals such as cleaning products, use of tools or household items that result in pressure on the hands, such as garden tools, knives, and screwdrivers, and wearing of loose fitting, well-ventilated shoes and clothes. Treatment may include use of topical moisturizing agents, topical anesthetics, or topical anti-inflammatory medications such as corticosteroid creams. In more severe cases, dose interruption and reduction may be warranted.

<u>Diarrhea</u>

Diarrhea has been reported with Sitravatinib treatment, though the mechanism remains unclear, as with other small molecule RTK inhibitors. Patients should be counseled that diarrhea is a possible side effect and advised to take loperamide or a similar medication as needed if diarrhea develops. Any patients developing dehydration or clinically significant electrolyte abnormalities should interrupt treatment, but treatment may be restarted once diarrhea is controlled. Investigators should also evaluate whether diarrhea may be attributable to the irAE of colitis.

Oliva M, *et al. J Immunother Cancer* 2022; 9:e003476. doi: 10.1136/jitc-2021-003476

Hemorrhagic Events

The risk of hemorrhagic events with Sitravatinib is unknown; however, such events have been reported with inhibitors of VEGFR. Patients with active hemoptysis or gastrointestinal bleeding should not take Sitravatinib, and suspension of treatment is recommended for patients developing clinically significant bleeding.

Thrombotic Events

Though thrombotic events (e.g., pulmonary embolism) have been reported with Sitravatinib and with inhibitors of VEGFR, the risk of such events with Sitravatinib is unknown. Precautions should be taken in patients with recent, clinically significant thrombotic events, and treatment should be discontinued in patients who develop clinically significant thromboembolic complications such as acute myocardial infarction or severe pulmonary embolism.

Thyroid Dysfunction Other than Immune-Mediated

Hypothyroidism and increases in TSH have been reported in patients taking Sitravatinib. Patients diagnosed with hypothyroidism should be treated with thyroid replacement and may continue treatment with Sitravatinib at the Investigator's discretion.

Decreased Left Ventricular Ejection Fraction

Decreased left ventricular ejection fraction (LVEF) has been reported with Sitravatinib. In addition, decreases of LVEF to <50% on-study were observed in patients undergoing scheduled multigated acquisition (MUGA) scans or echocardiograms. The dose of Sitravatinib should be permanently discontinued in patients without clinical evidence of congestive heart failure (CHF) but with an ejection fraction <50% and >20% below baseline.

Proteinuria

Although the risk with Sitravatinib is unknown, proteinuria has been described with other inhibitors of the VEGFR pathway. Patients who develop ≥2+ proteinuria should undergo 24-hour urine collection for assessment of urine protein; treatment with Sitravatinib should be discontinued in the presence of ≥2 grams of proteinuria/24 hours and may restart when protein levels decrease to less than 2 grams/24 hours. Patients who develop nephrotic syndrome should be withdrawn from treatment with Sitravatinib.

5.3.5.4 Dose modifications and delays for the combination therapy (Day 15).

Nivolumab will be given at the flat dose of 240mg and dose modifications will not be allowed. However, in the event of on-going adverse events on day 15 attributed to Sitravatinib that lead to its temporary or permanent discontinuation, Nivolumab administration should be delayed if it is deemed by the Principal Investigator. Patients should undergo blood and tumor collection for day 15 serum and tumor-based correlative studies as scheduled and described in Section 8.

Following resolution of the toxicity to grade 1 or to the patient's baseline value, the patient may resume Sitravatinib at 1 dose level lower. Nivolumab will be given at the flat dose of 240mg. Should a patient require a delay of day 15 Nivolumab for >7 days, or if the delay would result in the surgery date occurring after the initially planned window at study commencement, he/she will be discontinued from study. Please refer to Section 7 for reporting guidelines should this event occurs.
Please refer to Section 14.2 for guidelines on toxicity management.

5.3.6 Therapy after completion of protocol treatment

Patients will undergo standard of care surgical management within approximately 23-30 days following the start of study treatment. Patients who come off study prior to completion of study treatment will be managed in their physicians' discretion.

5.3.7 Follow-up for toxicities

Patients whose treatment is interrupted or permanently discontinued due to an AE or clinically significant laboratory value must be followed up at least once a week (or more if clinically indicated) until resolution or stabilization of the event, whichever comes first. Appropriate clinical experts should be consulted as deemed necessary.

All patients must be followed up for AEs and SAEs for 180 days after the last dose of Sitravatinib or Nivolumab, whichever occurs later.

Guidelines for toxicity management are outlined in section 14.2.

5.4 Study drug handling

5.4.1 Sitravatinib preparation, packaging and labelling

Sitravatinib will be provided by Mirati Therapeutics as 10 mg as well as 40 mg unit dose strength capsules. The composition of the drug product consists of a blend of MGCD516 free base drug substance, microcrystalline cellulose (Avicel® PH302) and polysorbate 80 (Tween® 80), and Aerosil® 200 Pharma. The blend is filled into Size 1 Light Blue Opaque (10 mg strength) or Swedish Orange Opaque (40 mg strength), hard gelatin capsules.

Sitravatinib drug product is packaged in 30-count, high-density polyethylene (HDPE), white opaque, round 60 cc bottles. A tamper-proof heat induction seal and a child-resistant closure are used. The provided bottles may be labeled for specific patient use and given to the patient.

Sitravatinib capsules should be stored in a secure storage area in the clinical trials pharmacy in cartons containing sealed HDPE bottles, at refrigerated conditions (2-8ºC) according to instructions on the carton label. After dispensing to the patient, the bottles are stored at ambient room temperature.

Sitravatinib capsules will be administered orally, once daily (QD), continuously up to maximum of 28-days. The starting dose for Sitravatinib will be 120 mg QD. Dose reduction might be applicable as stated in Section 5.3.5

The following guidelines should be followed for Sitravatinib administration:

- Dosing in the morning is preferred.
- Capsules should be taken on an empty stomach (at least 2-hour fast before each dose and no food for a minimum of 1 hour after each dose).
- Capsules should be taken with at least 200 mL (1 cup) of water.
- Patients should swallow the capsules whole and not chew them.
- If vomiting occurs after dosing, Sitravatinib doses should not be replaced.

On Day 15, when Sitravatinib and Nivolumab dosing are both scheduled, Nivolumab can be administered any time in relation to Sitravatinib.

5.4.2 Nivolumab preparation, packaging and labelling

Nivolumab is a sterile, preservative-free, non-pyrogenic, clear to opalescent, colorless to pale-yellow liquid that may contain light (few) particles. Nivolumab injection for intravenous infusion is supplied in either 40 mg or 100 mg single-dose vials. Each mL of Nivolumab solution contains Nivolumab 10 mg, mannitol (30 mg), pentetic acid (0.008 mg), polysorbate 80 (0.2 mg), sodium chloride (2.92 mg), sodium citrate dihydrate (5.88 mg), and Water for Injection, USP. The formulation may contain hydrochloric acid and/or sodium hydroxide to adjust pH to 6.

Visually inspect drug product solution for particulate matter and discoloration prior to administration. Nivolumab is a clear to opalescent, colorless to pale-yellow solution. Discard the vial if the solution is cloudy, discolored, or contains extraneous particulate matter other than a few translucent-to-white, proteinaceous particles. Do not shake the vial.

- Withdraw the required volume of Nivolumab and transfer into an intravenous container (PVC container, or non-PVC container)
- Dilute Nivolumab with either 0.9% Sodium Chloride Injection, USP or 5% Dextrose Injection, USP to prepare an infusion with a final concentration ranging from 1 mg/mL to 10 mg/mL.
- Mix diluted solution by gentle inversion. Do not shake.
- Discard partially used vials or empty vials of Nivolumab.
- Nivolumab infusion must be completed within 24 hours of preparation. If not used immediately, the infusion solution may be stored under refrigeration conditions: 2 to 8°C and protected from light for up to 24 hours (a maximum of 8 hours of the total 24 hours can be at room temperature 20 to 25°C and room light)
- Do not freeze.

The recommended dose of Nivolumab is 240 mg administered as an intravenous infusion over 30 minutes. Administer the Nivolumab infusion over 30 minutes through an intravenous line containing a sterile, non-pyrogenic, low protein binding in-line filter (pore size of 0.2 micrometer to 1.2 micrometer). Flush the intravenous line at end of infusion.

Monitoring for Nivolumab administration
Subjects will be monitored before and after the infusion with assessment of vital signs at the times specified in Table 7. Subjects are monitored (body temperature, pulse rate, blood pressure and pulse oximetry on room air).

As with any antibody, allergic reactions to dose administration are possible. Appropriate drugs and medical equipment to treat acute anaphylactic reactions must be immediately available, and study personnel must be trained to recognize and treat anaphylaxis. The study site must have immediate access to emergency resuscitation teams and equipment in addition to the ability to admit subjects to an intensive care unit if necessary. Guidelines for management of infusion-related reactions to Nivolumab are outlined in Table 14.

5.4.3 Drug supply and storage

Study treatments must be received by designated personnel at the study site, handled and stored safely and properly, and kept in a secured location with limited access. Upon receipt, Sitravatinib and Nivolumab should be stored according to the instructions specified on the drug label and/or IB.

5.5 Concomitant and post-study treatment(s)

5.5.1 Permitted concomitant therapies

All treatments that the Investigator considers necessary for a subject's welfare may be administered at the discretion of the Investigator in keeping with the community standards of medical care. All concomitant medication will be recorded in the eCRF including all prescription, over-the-counter, and IV medications and fluids. If changes occur during the trial period, documentation of drug dosage, frequency, route, and date may also be included on the CRF.

All concomitant medications received within 28 days before the first dose of trial treatment and 30 days after the last dose of trial treatment should be recorded. Concomitant medications administered 30 days after the last dose of trial treatment should be recorded for SAEs and AESIs as defined in Sections 7.1 and 7.2.

Anticoagulation is permitted if the patients are already at stable doses of warfarin or stable doses of low molecular weight heparin at the time of first Sitravatinib dose. Where applicable, INR should be monitored as clinically indicated per Investigator's discretion. However, ongoing anticoagulant therapy should be temporarily discontinued to allow tumor biopsy according to institutional guidelines.

Patients must be told to notify the investigational site about any new medications, herbal remedies and dietary supplements he/she takes after the start of the study

treatment. All medications (other than study treatment) and significant non-drug therapies (including physical therapy, herbal/natural medications and blood transfusions) administered during the study must be listed on the Concomitant Medications CRF.

5.5.2 Prohibited concomitant therapy

Subjects must be instructed not to take any medications, including over-the-counter products, without first consulting with the Investigator.

The following medications are considered exclusionary during the study:

- Any investigational anticancer therapy;
- Monoclonal antibodies against CTLA-4, PD-1, or PD-L1;
- Any concurrent chemotherapy, radiotherapy (except palliative radiotherapy after consultation with the medical monitor), immunotherapy, biologic or hormonal therapy for cancer treatment. Concurrent use of hormones for non-cancer-related conditions (e.g., insulin for diabetes and hormone replacement therapy) is acceptable;
- Immunosuppressive medications including, but not limited to systemic corticosteroids at doses exceeding 10 mg/day of prednisone or equivalent, methotrexate, azathioprine, and TNF-α blockers. Use of immunosuppressive medications for the management of investigational product-related AEs or in subjects with contrast allergies is acceptable. Temporary uses of corticosteroids for concurrent illnesses (e.g., food allergies, CT scan contrast hypersensitivity, etc.) are acceptable upon discussion with the study's Principal Investigator.
- Herbal and natural remedies should be avoided.

The use of systemic steroid therapy is permitted in the following settings: infusion reactions, immune-mediated toxicities and toxicity management, and replacement-dose steroids in the setting of adrenal insufficiency. Systemic steroid therapy required during the course of the study to manage other concurrent medical conditions is to be reviewed on a case-by-case basis by the Principal Investigator.

Medications or vaccinations specifically prohibited in the exclusion criteria are not allowed during the ongoing trial. If there is a clinical indication for one of these or other medications or vaccinations specifically prohibited during the trial, discontinuation from trial therapy or vaccination may be required. The investigator should discuss any questions regarding this with the Principal Investigator. The final decision on any supportive therapy or vaccination rests with the investigator and/or the subject's primary physician.

<u>Cytochrome P-450 Substrates</u>

Sitravatinib is a potential inducer of CYP 2B6 and 3A4, as well as a potential inhibitor of CYP 2C8, 2D6, and 3A4, though neither time dependent nor metabolism dependent inhibition has been observed. Medications that are substrates for CYP 2C8, 2D6 or 3A4 and are either sensitive substrates or have a narrow therapeutic

index should be used with caution during treatment with Sitravatinib. In vitro experiments in microsomes and recombinant human P450 enzymes suggest that Sitravatinib is metabolized by several cytochromes including CYP 3A4, 2B6, 2D6, and with a low risk of any one CYP demonstrating a disproportionate contribution to its metabolism. Although the clinical used dose of Sitravatinb has low potential for inhibiting or inducing CYP enzymes, caution should be used when administering Sitravatinib to patients taking medications that are strong inhibitors or inducers of the cytochrome P450 system. If a patient is taking such a medication, it is recommended that a different drug be prescribed for the condition being treated, if possible.

Medications for Gastric pH

The solubility of Sitravatinib is pH dependent. For this reason, medications that are associated with sustained increase in gastric pH may result in decreased exposure to Sitravatinib and should be avoided during treatment with Sitravatinib when possible. Patients still requiring gastric pH medications should switch from use of proton pump inhibitors or H_2 antagonists to use of antacids, which should be avoided 4 hours before and 2 hours after administration of investigational study treatment when possible.

Transporter Substrates and Inhibitors

Sitravatinib is an inhibitor of BCRP and P-gp transporters based on in vitro studies. Medications that are substrates for BCRP or P-gp transporters should be avoided during treatment with Sitravatinib. List provided in Appendix 14.4 (Please note that this may not be an exhaustive list, please refer to current websites for most up to date lists:https://www.fda.gov/Drugs/DevelopmentApprovalProcess/DevelopmentResources/DrugInteractionsLabeling/ucm093664.htm#PgpTransport).

Medications that Prolong QTc

The risk of QTc prolongation in patients receiving treatment with Sitravatinib has not been characterized. Use of medications known to prolong QTc and pose risk of Torsades de Pointes is prohibited during treatment with Sitravatinib (see Appendix 14.4).

5.5.3 Rescue Medications & Supportive Care

Supportive Care Guidelines

Subjects should receive appropriate supportive care measures as deemed necessary by the treating investigator. Suggested supportive care measures for the management of drug-related adverse events are outlined in Appendix 14.3. Where appropriate, these guidelines include the use of oral or intravenous treatment with corticosteroids as well as additional anti-inflammatory agents if symptoms do not improve with administration of corticosteroids. Note that several courses of steroid tapering may be necessary as symptoms may worsen when the steroid dose is decreased. For each disorder, attempts should be made to rule out other causes such as metastatic disease or bacterial or viral infection, which might require additional supportive care. The treatment guidelines are intended to be applied when the investigator determines the events to be related to Sitravatinib and/or Nivolumab.

Note: if after the evaluation the event is determined not to be related, the investigator is instructed to follow the AESI reporting guidelines as outlined in Section 7.5.

5.5.4 Diet/Activity/Other Considerations

Diet

Subjects should maintain a normal diet unless modifications are required to manage an AE such as diarrhea, nausea or vomiting.

Contraception

It is not known whether Sitravatinib presents a risk to the embryo or the fetus.
Sitravatinib is contraindicated in women who are pregnant or lactating.
Female patient of child-bearing potential:

- Females of childbearing potential who are sexually active with a non-sterilized male partner must use at least 1 **highly** effective method of contraception (Table 6) from the time of screening and must agree to continue using such precautions for 180 days after the last dose of Sitravatinib and/or Nivolumab therapy, whichever occurs later.
- Non-sterilised male partners of a female patient must use male condom plus spermicide throughout this period. Cessation of birth control after this point should be discussed with a responsible physician.
- Not engaging in sexual activity for the total duration of the drug treatment and the drug washout period is an acceptable practice; however, periodic abstinence, the rhythm method, and the withdrawal method are not acceptable methods of birth control.
- Female patients should also refrain from breastfeeding throughout this period.

Male patients with a female partner of childbearing potential:

- Non-sterilized males who are sexually active with a female partner of childbearing potential must use a male condom plus spermicide from screening through 180 days after receipt of the last dose of Sitravatinib or Nivolumab therapy whichever occurs later.
- Not engaging in sexual activity from screening through 180 days after receipt of the last dose of Nivolumab or Sitravatinib therapy is an acceptable practice; however, occasional abstinence, the rhythm method, and the withdrawal method are not acceptable methods of contraception. Throughout this period, male patients should refrain from sperm donation, and female patients should refrain from egg cell donation.
- Female partners (of childbearing potential) of male patients must also use a highly effective method of contraception throughout this period (Table 6).

N.B Females of childbearing potential are defined as those who are not surgically sterile (ie, bilateral tubal ligation, bilateral oophorectomy, or complete hysterectomy) or post-menopausal.

Women will be considered post-menopausal if they have been amenorrheic for 12 months without an alternative medical cause. The following age-specific requirements apply:

- Women <50 years of age would be considered post-menopausal if they have been amenorrheic for 12 months or more following cessation of exogenous hormonal treatments and if they have luteinizing hormone and follicle-stimulating hormone levels in the post-menopausal range for the institution or underwent surgical sterilization (bilateral oophorectomy, bilateral salpingectomy or hysterectomy).
- Women ≥50 years of age would be considered post-menopausal if they have been amenorrheic for 12 months or more following cessation of all exogenous hormonal treatments, had radiation-induced menopause with last menses >1 year ago, had chemotherapy-induced menopause with last menses >1 year ago, or underwent surgical sterilization (bilateral oophorectomy, bilateral salpingectomy or hysterectomy).

Highly effective methods of contraception defined as one that results in a low failure rate (ie, less than 1% per year) when used consistently and correctly are described in Table 6. Note that some contraception methods are not considered highly effective (e.g. male or female condom with or without spermicide; female cap, diaphragm, or sponge with or without spermicide; non-copper containing intrauterine device; progestogen-only oral hormonal contraceptive pills where inhibition of ovulation is not the primary mode of action [excluding Cerazette/desogestrel which is considered highly effective]; and triphasic combined oral contraceptive pills).

Barrier/Intrauterine methods	Hormonal Methods
• Copper T intrauterine device<br>• Levonorgesterel-releasing intrauterine system (eg, Mirena®)[a]	• Etonogestrel implants: e.g. Implanon or Norplan<br>• Intravaginal device: e.g. ethinylestradiol and etonogestrel<br>• Medroxyprogesterone injection: e.g. Depo-Provera<br>• Normal and low dose combined oral contraceptive pill<br>• Norelgestromin/ethinylestradiol transdermal system<br>• Cerazette (desogestrel)

Table 6. Highly effective methods of contraception (<1% failure rate)

[a] This is also considered a hormonal method

Subjects should be informed that taking the study medication may involve unknown risks to the fetus (unborn baby) if pregnancy were to occur during the study. In order to participate in the study they must adhere to the contraception requirement (described above) for the duration of the study and during the follow-up period. If there is any question that a subject will not reliably comply with the requirements for contraception, that subject should not be entered into the study.

Blood donation

Subjects should not donate blood while participating in this study, and for at least 180 days following the last dose of Sitravatinib or Nivolumab therapy, whichever occurs later.

Use in pregnancy

If a subject inadvertently becomes pregnant while on treatment with Nivolumab or Sitravatinib, the subject will immediately be removed from the study. The site will contact the subject at least monthly and document the subject's status until the pregnancy has been completed or terminated. The outcome of the pregnancy will be reported without delay and within 24 hours if the outcome is a serious adverse experience (e.g., death, abortion, congenital anomaly, or other disabling or life-threatening complication to the mother or newborn).

The study investigator will make every effort to obtain permission to follow the outcome of the pregnancy and report the condition of the fetus or newborn to Mirati Therapeutics. If a male subject impregnates his female partner the study personnel at the site must be informed immediately and the pregnancy reported and followed as described above.

Use in Nursing Women

Since many drugs are excreted in human milk, and because of the potential for serious adverse reactions in the nursing infant, subjects who are breast-feeding are not eligible for enrollment.

5.6 Treatment compliance

Compliance will be assured by administration of the study treatment under the supervision of Investigator or his/her designee.

5.6.1 Accountability

The Investigator or designee must maintain an accurate record of the shipment and dispensing of study treatment according to local institutional drug accountability processes.
At study close-out, and, as appropriate during the course of the study, the Investigator will destroy as per local procedures any expired or unused study treatment.

5.6.2 Disposal and destruction

The study drug supply can be destroyed at the local facility, Drug Supply group or third party, as appropriate.

5.7 Discontinuation of investigational product

5.7.1 Procedures for discontinuation of a subject from investigational product

Patients may voluntarily discontinue from the study treatment for any reason at any time. If a patient decides to discontinue from the study treatment, the Investigator

must make every effort (e.g. telephone, e-mail, letter) to determine the primary reason for this decision and record this information in the patient's chart and on the appropriate CRF pages. They may be considered withdrawn if they state an intention to withdraw, fail to return for visits, or become lost to follow-up for any other reason.

The Investigator should discontinue study treatment for a given patient if, on balance, he/she believes that continuation would be detrimental to the patient's well-being, in addition to the criteria described in Section 5.3.5.

Study treatment may be discontinued if any of the following occur:

- Intolerable adverse event,
- Lost to follow-up,
- Physician's decision,
- Progressive disease per RECIST version 1.1,
- Study terminated by the Sponsor and Mirati Therapeutics.
- Subject/guardian decision,
- Protocol deviations that result in significant risk to the patients' safety, and/or
- Technical problems

Patients will be withdrawn from study if any of the following occur:

- Death, or
- Pregnancy

Patients who discontinue study treatment should NOT be considered withdrawn from the study, unless they fail to fulfill the evaluability criteria for correlative study analysis, as detailed in Section 3.2.3 They should return for the assessments indicated in Table 7 for follow up 4-8 weeks after day of surgery. If they fail to return for these assessments for unknown reasons, every effort (e.g. telephone, email, letter) should be made to contact them. If a patient discontinues study treatment, but continues study assessments, the patient remains on study until such time as he/she completes protocol criteria for ending study assessments. At that time, the reason for study completion should be recorded on the appropriate CRF page.

If a patient discontinue study treatment and is non-evaluable for correlative study analyses, as per criteria defined in Section 3.2.3, then they should be considered withdrawn from study.

5.7.2 Replacement policy

If a patient is considered to be non-evaluable for correlative study analyses, enrollment of a new patient to the current cohort will be considered at discretion of the Principal Investigator.

Patients may voluntarily withdraw consent to participate in the study for any reason at any time. Withdrawal of consent occurs only when a patient does not want to participate in the study any longer, and does not want any further visits or assessments, and does not want any further study related contact.

Tumor Immunotherapy Program will continue to retain and use all research results that have already been collected for the study evaluation. All biological samples that have already been collected may be retained and analyzed at a later date.

If a patient withdraws consent, the Investigator must make every effort (e.g. telephone, e-mail, letter) to determine the primary reason for this decision and record this information. Study treatment must be discontinued and no further assessments conducted. Further attempts to contact the patient are not allowed unless safety findings require communication or follow up.

5.7.3 Lost to follow up

For patients whose status is unclear because they fail to appear for study visits without stating an intention to withdraw consent, the Investigator should show "due diligence”" by contacting the patient, family or family physician as agreed in the informed consent and by documenting in the source documents steps taken to contact the patient, e.g. dates of telephone calls, registered letters, etc. A patient should not be considered lost to follow-up until due diligence has been completed. Patients lost to follow up should be recorded as such on the appropriate CRF.

6. COLLECTION OF STUDY VARIABLES

6.1 Study flow and visit schedule

Table 7 lists all of the assessments and indicates with an “X” the visits when they are performed. All data obtained from these assessments must be supported in the patient’s source documentation. No CRF will be used as a source document.

All screening assessments must be performed $\leq$ 10 days to first dose of Sitravatinib. The exception is CT scans or MRI scans which should be done as a standard of care within clinically acceptable windows.

If the assessments required on Day 1 are performed as part of the screening evaluations within 72 hours prior to the first dose of study treatment, they do not need to be repeated on Day 1. Laboratory and radiological assessments performed as part of standard of care prior to signing informed consent may be used if performed within the screening time window.

During the course of the study visits, test and/or procedures should occur on schedule whenever possible.

6.1.1 Screening

The study informed consent form must be signed and dated before any screening procedures are performed, except for laboratory and radiological evaluations performed as part of standard of care.

Patients will be evaluated against study inclusion and exclusion criteria as described in Sections 4.1 and 4.2. Screening assessments as detailed in Table 7 and Section 3.3.1. must be repeated if performed outside of the specified screening window.

Exceptions are allowed on a case-by-case basis in discussion with the Principal Investigator. Patients are allowed to re-screen after abnormal labs or symptoms are corrected and/or treated.

Information to be collected on screening failures

A patient who signed an Informed Consent Form but failed to be started on treatment for any reason will be considered a screen failure. If a patient is found to be not eligible after signing the main study consent, he/she will be considered as a screen failure, and data will be handled in the same manner.

The demographic information, informed consent, and Inclusion/Exclusion pages must also be completed for screen failure patients. No other data will be entered into the clinical database for patients who are screen failures, unless the patient experienced a SAE during screening.

Patient demographics and other baseline characteristics

Data to be collected will include general patient demographics, relevant medical history and current medical conditions, diagnosis and extent of tumor, prior medication, procedures, significant non-drug therapies and any other assessments that are done for the purpose of determining eligibility for inclusion in the study.

6.1.2 Treatment period

Timing of required assessments and visit windows are detailed in Table 7. Treatment period is 21 to 28 days, with a 48 hours washout period prior to day of surgery.

6.1.3 Safety follow up period

All patients will have a safety evaluation visit 4-8 weeks after surgery. All AEs suspected to be related to study treatment should be followed up weekly or as clinically indicated until resolution or stabilization. See Section 7 for details on safety monitoring and reporting.

Any ongoing or planned antineoplastic therapies and/or procedures since discontinuation of study drug will be collected during this follow-up period.

6.1.4 Disease progression follow up period

Upon completion of the 4-8 weeks safety follow up visit, patients will be followed for disease progression every 3 months (can be done by telephone call and/or through medical record follow up evidence) until the end of the study is reached, unless they withdraw consent or are lost to follow-up. Antineoplastic therapies and/or procedures since discontinuation of study drug will be collected during this follow-up period.

	Screening[1]	Day 1[1]	Day 15[20]	Presurgery[4,15]	Surgery[16]	Follow-up/EOT[17]
Sitravatinib[2]		X	X	X		
Nivolumab[3]			X			
Written informed consent	X					
**History and Physical Examination**						
Demographics	X					
Medical history	X					
Physical examination	X	X	X	X[4]		
ECOG PS	X	X	X	X[4]		
Vital signs	X	X[5]	X	X[4,5]		
Weight	X	X	X	X[4]		
Height	X					
Pregnancy test (serum/urine)[6]	X					
**Photographic Assessment**						
Photographic documentation[7]	X		X		X	
**Radiological Assessments**						
CT or MRI	X					
**Laboratory Assessments**						
Hematology[8]	X	X	X	X[4]		
Serum chemistry[9]	X	X	X	X[4]		
Coagulation[10]	X			X[4]		
Urinalysis	X		X	X[4]		
**Pharmacodynamic assessments**						
Blood collection	X		X[11]	X		
Tumor biopsy/Collection	X[12]		X[13]		X	

**Pharmacokinetic assessments**						
Blood collection		X[14]	X[14,15]		X[14,15]	
**Adverse events/safety**						
Adverse Events	X	X	X	X	X	X
Concomitant medications	X	X	X	X		X
MUGA	X					
Single 12-Lead ECG	X[18]		X[18]	X[18]		
**Exploratory (optional)**						
^{18}FAZA PET[19]	X			X		

Table 7. Schedule of Assessments

1. All screening assessments must be performed $\leq$ 10 days prior to the first dose of Sitravatinib. The exception is CT scans or MRI scans which should be done as a standard of care within clinically acceptable windows. If the assessments required on Day 1 are performed as part of the screening evaluations within 72 hours prior to the first dose of study treatment, they do not need to be repeated on Day 1. Laboratory and radiological assessments performed as part of standard of care prior to signing informed consent may be used if performed $\leq$ 10 days to first dose of Sitravatinib (with exception of CT scans or MRI scans). MUGA scan is to be performed within 4 weeks prior to the first dose of Sitravatinib.
2. Sitravatinib is to be given once daily on a continuous basis starting on Day 1 until 48 hours prior to scheduled surgery, or for a maximum of 28 days.
3. Nivolumab to be given on Day 15, as a single infusion at a dose of 240mg, over 30 minutes of duration. Patients are to be monitored (pulse rate and blood pressure) pre and post infusion.
4. Pre-surgery assessments are to be performed $\leq$ 7 days prior to surgery. Surgery will be planned between day 23 and 30.
5. Scheduled vital signs should precede pharmacokinetic sample collection in all cases.
6. Women of childbearing potential.
7. Photographic documentation of primary tumor is required when feasible at baseline, Day 15 and operatively. These assessments may be performed within a clinically acceptable window, per Investigator discretion.
8. Complete blood counts with differentials, and platelet counts.
9. Sodium, chloride, potassium, bicarbonate, creatinine, AST (alanine aminotransferase), ALT (alanine aminotransferase), ALP (alkaline phosphatase), albumin, bilirubin, albumin, calcium, magnesium, phosphate, amylase and lipase.
10. PT (prothrombin time), APTT (activated partial thromboplastin time), INR (international normalized ratio).
11. Blood collection on Day 15 is to be performed at $\leq$48 hours prior to Nivolumab treatments on Day 15. Should a patient require a delay of Day 15 study treatments, please refer to section 5.3.5.4 for guidance on pharmacodynamics sample collection.
12. Baseline biopsy is to be performed $\leq$ 10 days prior to the start of study treatment. Archival tissue sample will be requested if available.

13. On-treatment tumor biopsy is to be performed $\leq$ 48 hours prior to Nivolumab treatment on day 15. Should a patient require a delay of day 15 study treatments, please refer to section 5.3.5.6 for guidance on tumor biopsy.
14. Pharmacokinetic sample collections are to be performed at a single time-point at baseline, on the day of on-treatment biopsy and on the day of surgery.
15. A 24-hour window is allowed for pharmacokinetic sample collection.
16. Surgery will be performed within approximately 23-30 days following the start of study treatment (Day 1).
17. Follow-up assessments are to be performed approximately four to eight weeks after completion of surgery.
18. ECG assessments will include evaluation of rhythm, heart rate and QT intervals.
19. ^{18}FAZA PET testing is optional, however is strongly encouraged to be performed within screening and pre-surgery assessment periods.
20. History, physical and laboratory assessments on Day 15 may be performed up to 1-day prior to Day 15 Nivolumab dosing.

6.2 Clinical assessments

Physical examination will be performed according to Table 7.

At Screening and study Day 1 prior to Sitravatinib dosing, a complete physical examination will be performed and will include the examination of general appearance, skin, neck, eyes, ears, nose, throat, lungs, heart, abdomen, back, lymph nodes, extremities and neurological system. If indicated based on medical history and/or symptoms, rectal, external genitalia, breast, and pelvic exams will be performed.

From Day 15 onwards, a short physical examination will be performed. A short physical exam will include the examination of general appearance, vital signs (body temperature, pulse rate, blood pressure, and pulse oximetry on room air) and body sites as directed by symptoms.

Significant findings that were present prior to the signing of the informed consent must be included in the Medical History CRF page. Significant new findings that begin or worsen after informed consent must be recorded on the Adverse Event CRF page.

6.2.1 Vital signs

Vital signs (body temperature, pulse rate, blood pressure, and pulse oximetry on room air) must be performed before dosing on Days 1 and 15 as indicated in Table 7.

Vital signs should be assessed on the scheduled day, even if study treatment is being withheld. More frequent examinations may be performed at the discretion of the Investigator if medically indicated, and will be recorded as unscheduled assessment.

6.2.2 Height and weight

Height in centimeters (cm) and body weight (to the nearest 0.1 kilogram in indoor clothing, but without shoes) will be measured as indicated in Table 7.

6.2.3 Performance status

Performance status is determined as indicated in Table 8.

ECOG Status	
0	Fully active, able to carry on all pre-disease performance without restriction
1	Restricted in physically strenuous activity but ambulatory and able to carry out work of a light or sedentary nature (e.g., light house work, office work)
2	Ambulatory and capable of all self-care but unable to carry out any work activities. Up and about more than 50% of waking hours
3	Capable of only limited self-care, confined to bed or chair more than 50% of waking hours
4	Completely disabled. Cannot carry on any self-care. Totally confined to bed or chair

Table 8. Eastern Cooperative Oncology Group (ECOG) performance status

Oliva M, et al. J Immunother Cancer 2022; 9:e003476. doi: 10.1136/jitc-2021-003476

6.2.4 Laboratory evaluations

All laboratory parameters assessed for safety purposes will be evaluated locally. Refer to Table 9 for a summary of the parameters to be evaluated. On day 15, samples for these parameters will be collected prior to the oral dosing of Sitravatinib and infusion of Nivolumab.

More frequent evaluations may be performed at the Investigator's discretion if medically indicated. These results should be recorded as unscheduled assessments.

If at any time a patient has laboratory parameters obtained from a different outside laboratory, Tumor Immunotherapy Program must be provided with a copy of the certification and a tabulation of the normal ranges for that laboratory.

6.2.5 Cardiac assessments

An assessment of cardiac function by MUGA and ECG will be performed as per the assessment schedule in Table 7.

Clinically significant abnormalities present at screening should be reported on the Medical History CRF page. New or worsened clinically significant findings occurring after informed consent must be recorded on the Adverse Events CRF page.

6.2.6 Photographic documentation

Whenever feasible, intra-oral lesions will be photographed with high-resolution digital techniques per schedule described in Table 7, to document changes in the morphology of tumor lesion(s) while on study.

7. SAFETY MONITORING AND REPORTING

7.1 Adverse events

7.1.1 Definitions and reporting

An adverse event is defined as the appearance of, or worsening of any pre-existing, undesirable sign(s), symptom(s), or medical condition(s) that occur after a patient's signed informed consent has been obtained.

Abnormal laboratory values or test results occurring after informed consent constitute adverse events only if they induce clinical signs or symptoms, are considered clinically significant, require therapy (e.g., hematologic abnormality that requires transfusion or haematological stem cell support), or require changes in study medication(s).

Adverse events that begin or worsen after informed consent should be recorded in the Adverse Events CRF. Conditions that were already present at the time of informed consent should be recorded in the Medical History page of the patient's CRF.

Adverse event monitoring should be continued for 180 days following the last dose of Sitravatinib or Nivolumab, whichever occurs later. Adverse events (including lab abnormalities that constitute AEs) should be described using a diagnosis whenever

possible, rather than individual underlying signs and symptoms. When a clear diagnosis cannot be identified, each sign or symptom should be reported as a separate Adverse Event.

During follow-up period, only Adverse Events considered at least possibly related to study drug(s) need to be captured.

Adverse events will be assessed according to the Common Terminology Criteria for Adverse Events (CTCAE) version 5.0.

As far as possible, each adverse event should be evaluated to determine:

- The severity grade (CTCAE Grade 1-4),
- Its duration (start and end dates),
- Its relationship to the study treatment (reasonable possibility that AE is related: No, Yes),
- Action taken with respect to study or investigational treatment (none, dose adjusted, temporarily interrupted, permanently discontinued, unknown, not applicable),
- Whether medication or therapy was given (no concomitant medication/non-drug therapy, concomitant medication/non-drug therapy),
- Outcome (not recovered/not resolved, recovered/resolved, recovering/resolving, recovered/resolved with sequelae, fatal, unknown), and
- Whether it is serious, where a serious adverse event (SAE) is defined as in section 7.2.

All adverse events should be treated appropriately. If a concomitant medication or non-drug therapy is given, this action should be recorded on the Concomitant Medications CRF.

Once an adverse event is detected, it should be followed until its resolution or until it is judged to be permanent, and assessment should be made at each visit (or more frequently, if necessary) of any changes in severity, the suspected relationship to the study treatment, the interventions required to treat it, and the outcome.

The Investigator is responsible for evaluating all adverse events to determine whether criteria for a serious adverse event, as defined in section 7.2 below, are met. The Investigator is responsible for reporting serious adverse events as described in section 7.2.

7.1.2 Laboratory test abnormalities

Definitions and reporting

Laboratory abnormalities that constitute an adverse event in their own right (are considered clinically significant, induce clinical signs or symptoms, require concomitant therapy or require changes in study treatment), should be recorded on the Adverse Events CRF. Whenever possible, a diagnosis, rather than a symptom should be provided (e.g. anemia instead of low hemoglobin). Laboratory abnormalities that meet the criteria for Adverse Events should be followed until they have returned to normal or an adequate explanation of the abnormality is found. When an abnormal

laboratory or test result corresponds to a sign/symptom of an already reported adverse event, it is not necessary to separately record the lab/test result as an additional event. Laboratory abnormalities that do not meet the definition of an adverse event should not be reported as adverse events. Grade 3 or 4 adverse events (severe) as per CTCAE version 5.0 do not automatically indicate an SAE unless it meets the definition of serious as defined below and/or as per Investigator's discretion.

7.2 Serious adverse events

7.2.1 Definitions

Serious adverse event (SAE) is defined as one of the following:

- Is fatal or life-threatening
- Results in persistent or significant disability/incapacity
- Constitutes a congenital anomaly/birth defect
- Is medically significant, i.e., defined as an event that jeopardizes the patient or may require medical or surgical intervention to prevent one of the outcomes listed above, or
- Requires inpatient hospitalization or prolongation of existing hospitalization.

Note that hospitalizations for the following reasons should not be reported as serious adverse events:

- Routine treatment or monitoring of the studied indication, not associated with any deterioration in condition (specify what this includes),
- Elective or pre-planned treatment for a pre-existing condition that is unrelated to the indication under study and has not worsened since signing the informed consent, or
- Social reasons and respite care in the absence of any deterioration in the patient's general condition.

Note that treatment on an emergency outpatient basis that does not result in hospital admission and involves an event not fulfilling any of the definitions of a SAE given above is not a serious adverse event. If there is any uncertainty about an adverse event being due only to the disease under study, it should be reported as an AE or SAE.

Any adverse event that result in a delay of day 15 study treatments for >7 days, or if the delay would result in the surgery date occurring after the initially planned window at study commencement, should be reported as a SAE.

Post-surgical complications that require a prolonged and/or an additional hospital admission should be reported both as a SAE, as defined in Sections 7.2 and 7.5.

Disease progression as defined per RECIST v1.1 criteria during the treatment and pre-operative periods (day 1 up to day prior to surgery) will not be considered a SAE, as the treatment window proposed in this study is within institutional standard practices. In the event of disease progression during study, the patient should whenever possible, undergo full radiological restaging including CT of the head, neck, chest, and abdomen.

7.2.2 SAE Reporting

For patients who sign the study informed consent form (ICF), SAE collection starts at time of main study informed consent whether the patient is a screen failure or not.

Information about all SAEs is collected and recorded on the Serious Adverse Event Report Form. All applicable sections of the form must be completed in order to provide a clinically thorough report. The Investigator must assess and record the relationship of each SAE to each specific study treatment (if there is more than one study treatment). The site staff will send the completed, signed SAE Report Form by fax or email to the Sponsor - Tumor Immunotherapy Program - Central Office within 24 hours/1 business day of learning of its occurrence. The Sponsor is also required to notify Mirati Therapeutics, using the contact details supplied in Section 7.2.4.
Follow-up information is sent to the same contact(s) to whom the original SAE Report Form was sent, using a new SAE Report Form stating that this is a follow-up to the previously reported SAE and giving the date of the original report. Each re-occurrence, complication, or progression of the original event should be reported as a follow-up to that event regardless of when it occurs. The follow-up information should describe whether the event has resolved or continues, if and how it was treated, whether the blind was broken or not, and whether the patient continued or withdrew from study participation.

If the SAE is not previously documented in the Investigator's Brochure or Package Insert (new occurrence) and is thought to be related to the study treatment, the Sponsor will send a notification to Mirati Therapeutics, as it sees appropriate, who may need to issue an Investigator Notification (IN), to inform all Investigators involved in any study with the same drug that this SAE has been reported. Suspected Unexpected Serious Adverse Reactions (SUSARs) will be collected and reported to the competent authorities and relevant ethics committees.

Adverse events and serious adverse events will be recorded from time of signature of informed consent, throughout the treatment period and including the follow-up period (180 days after the last dose of Sitravatinib or Nivolumab, which ever occur later).

During the course of the study all AEs and SAEs should be proactively followed up for each subject. Every effort should be made to obtain a resolution for all events, even if the events continue after discontinuation/study completion.

The investigator is responsible for following all SAEs until resolution, until the subject returns to baseline status, or until the condition has stabilized with the expectation that it will remain chronic, even if this extends beyond study participation.

7.2.3 Other Events Requiring Immediate Reporting

Overdose

An overdose is defined as a subject receiving a dose of investigational product in excess of that specified in the Investigator's Brochures, unless otherwise specified in this protocol.

An overdose with associated AEs is recorded as the AE diagnosis/symptoms on the relevant AE modules in the eCRF.

An overdose without associated symptoms is reported on source documents.

If an overdose on a study regimen occurs during the course of the study, then the investigator or other site personnel should inform the appropriate Mirati Therapeutics representative(s) within 24 hours of when he or she becomes aware of it. Overdose does not automatically make an AE serious, but if the consequences of the overdose are serious, for example death or hospitalization, the event is serious and must be reported as an SAE (see Sections 7.1 and 7.2).

The investigator will use clinical judgment to treat any Nivolumab or Sitravatinib overdose. Additionally, episodes of overdose will be investigated to determine the cause and review the processes. Any contributing factors will be assessed and a management plan to ensure that future episodes of overdosing do not happen, will be implemented.

Hepatic Function Abnormality

Adverse events of hepatic function abnormality of special interest to the Sponsor are defined as any increase in ALT or AST to greater than 3 × ULN and concurrent increase in bilirubin to greater than 2 × ULN (i.e., Hy's law cases). Concurrent findings are those that derive from a single blood draw or from separate blood draws taken within 8 days of each other. In the event of hepatic function abnormality where the etiology is unknown, timely follow-up investigations and inquiries should be initiated by the investigational site, based on medical judgment, to make an informed decision regarding the etiology of the event.

If the underlying diagnosis for the hepatic function abnormality is known (including progression of pre-existing disease) the diagnosis should be recorded as an AE/SAE.

If the underlying diagnosis for the hepatic function abnormality remains unknown, the term "hepatic function abnormal" should be used to report the AE/SAE.

Hepatic function abnormality of unknown etiology, or which is considered attributable to investigational product, is required to be reported as "hepatic function abnormal" within 24 hours of knowledge of the event to the Sponsor as an SAE (see Section 7.2.2). The investigator will review the data with the medical monitor. The investigator should then use clinical judgment to establish the cause based on local standard of care and follow the subject by conducting testing as clinically indicated.

If, after appropriate workup, in the opinion of the investigator, the underlying diagnosis for the abnormality remains unexplained, or is considered attributable to investigational product, permanent discontinuation of dosing for the study subject should be considered.

Each reported event of hepatic function abnormality will be followed by the investigator and evaluated by the Sponsor. The Sponsor is also required to notify

Mirati Therapeutics. If the etiology of the event remains unconfirmed and/or is considered related to investigational product, a prompt cumulative review of safety data and the circumstances of the event in question will be conducted and assessed by the safety review committee (or equivalent) to determine whether continued dosing of current study subjects and/or study entry should be interrupted, whether the protocol will be modified, or whether the study will be discontinued permanently. Review and approval by the safety review committee (or equivalent) is required for resumption of subject dosing or study entry in the event that the study is interrupted. When applicable, regulatory authorities and local REBs will be notified of any actions taken with the study.

Pregnancy Maternal exposure

If a patient becomes pregnant during the course of the study, the investigational products should be discontinued immediately.

Pregnancy itself is not regarded as an AE unless there is a suspicion that the investigational product(s) under study may have interfered with the effectiveness of a contraceptive medication. Congenital abnormalities or birth defects and spontaneous miscarriages should be reported and handled as SAEs. Elective abortions without complications should not be handled as AEs. The outcome of all pregnancies (spontaneous miscarriage, elective termination, ectopic pregnancy, normal birth, or congenital abnormality) should be followed up and documented even if the patient was discontinued from the study.

If any pregnancy occurs in the course of the study, then the Investigator or other site personnel should inform the Tumor Immunotherapy Program Central Office within 24 hours/1 business day, i.e. immediately.

The Tumor Immunotherapy Program Central Office coordinator will work with the Investigator to ensure that all relevant information is provided to Mirati Therapeutics within 1 to 5 business days for SAEs and within 30 days for all other pregnancies.

The same timelines apply when outcome information is available.

Paternal exposure

Male patients should refrain from fathering a child or donating sperm during the study and for 180 days after the last dose of Sitravatinib or Nivolumab, whichever occurs later.

Pregnancy of the patient's partner is not considered to be an AE. However, the outcome of all pregnancies (spontaneous miscarriage, elective termination, ectopic pregnancy, normal birth, or congenital abnormality) occurring from the date of the first dose until 180 days after the last dose of Sitravatinib or Nivolumab, whichever occurs later, should, if possible, be followed up and documented.

Where a report of pregnancy is received, prior to obtaining information about the pregnancy, the Investigator must obtain the consent of the patient's partner.

Therefore, the local study team should adopt the generic ICF template in line with local procedures and submit it to the local REB prior to use.

7.2.4 Expedited Reporting to Tumor Immunotherapy Program and Mirati

The Investigator should inform the Tumor Immunotherapy Program Central Office of any **SAE within 24 hours/1 business day of being aware of the event**. This must be documented on the Tumor Immunotherapy Program study-specific SAE form.

This SAE form must be completed and supplied to the Tumor Immunotherapy Program Central Office within 24 hours/1 business day at the latest on the following working day. The Tumor Immunotherapy Program Central Office is required to notify Mirati Therapeutics within 24 hours/1 business day at the latest on the following working day. SAEs requiring a Suspected Unexpected Serious Adverse Reactions (SUSARs) that are unexpected and related to Sitravatinib need to be forwarded to Mirati Therapeutics.

The initial report must be as complete as possible, including details of the current illness and serious adverse event, and an assessment of the causal relationship between the event and the investigational product(s). Information not available at the time of the initial report (e.g., an end date for the adverse event or laboratory values received after the report) must be documented on a follow-up SAE form.

Tumor Immunotherapy Program Central Office Contact:

TIP@uhn.ca

Mirati Therapeutics Contact:

Mirati Therapeutics
Email: wilsafety@ppdi.com
Fax: +1-888-488-9697
Phone (for fax issues): +1-800-201-8725

Investigative site must also indicate, either in the SAE report or the cover page, the causality of events in relation to all study medications and if the SAE is related to disease progression, as determined by the principal investigator.

All SAEs have to be reported to Mirati Therapeutics, whether or not considered causally related to the investigational product. All SAEs will be documented. The investigator is responsible for informing the IRB and/or the Regulatory Authority of the SAE as per local requirements.

7.2.5 Pregnancy reporting by Investigator to Mirati Therapeutics

To ensure patient safety, each pregnancy occurring while the patient is on study treatment must be reported to Mirati Therapeutics within 24 hours/1 business day of learning of its occurrence. The pregnancy should be followed up to determine outcome, including spontaneous or voluntary termination, details of the birth, and the presence or absence of any birth defects, congenital abnormalities, or maternal and/or

newborn complications. After the mother has provided consent, the newborn will be followed-up for 3 months.

Any SAE experienced during pregnancy must be reported on the SAE Report Form.

7.2.6 Reporting to Health Canada

Adverse drug reactions that are **Serious, Unexpected, and at least Possibly Related to the drug,** and that have not previously been reported in the Investigator's Brochure, or reference safety information document will be reported promptly to Health Canada in writing by the Tumor Immunotherapy Program. A clear description of the suspected reaction should be provided along with an assessment as to whether the event is drug or disease related. The Tumor Immunotherapy Program shall notify Health Canada by telephone or by fax of any unexpected fatal or life threatening experience associated with the use of the drugs as soon as possible but no later than 7 days after becoming aware of the information. Where the Adverse Drug Reaction is neither fatal nor life-threatening, it will be reported within 15 days after becoming aware of the information.

7.3 Reporting of adverse events to the Research Ethics Board (REB)

The Principal Investigator is required to notify her Research Ethics Board (REB) of a serious adverse event according to institutional policy.

7.4 Adverse Event updates

Mirati Therapeutics shall notify the Investigator of any AE associated with the use of study drugs in this study or in other studies that is both serious and unexpected.

Mirati Therapeutics will forward SUSARs occurring with Sitravatinib to the Tumor Immunotherapy Program in the following timelines:

- Reports of fatal or life threatening Serious Adverse Drug Reactions will be sent within five (5) calendar days of Receipt Date.
- Reports of Serious Adverse Drug Reactions (other than fatal or life threatening) will be sent within twelve (12) calendar days of Receipt Date.

Any finding from tests in laboratory animals that suggests a significant risk for human subjects including reports of mutagenicity, teratogenicity or carcinogenicity.

The Investigator shall notify his/her REB promptly of these new serious and unexpected AE(s) or significant risks to subjects. The Investigator must keep copies of all AE information, including correspondence with the Sponsor, Mirati Therapeutics and the REB, on file.

7.5 Adverse events of special interest

An adverse event of special interest (AESI) is one of scientific and medical interest specific to understanding of the Investigational Product and may require close

monitoring and rapid communication by the investigator to the Sponsor and Mirati Therapeutics. An AESI may be serious or non-serious. The rapid reporting of AESIs allows ongoing surveillance of these events in order to characterize and understand them in association with the use of this investigational product.

AESIs require expedited reporting within 24 hours/1 business day of learning of its occurrence to the Sponsor (Tumor Immunotherapy Program), using the contact details outlined in section 7.2.4.

No AESI have been related to Sitravatinib. Further information can be found in the current version of the Sitravatinib Investigator Brochure.

AESIs for Nivolumab include but are not limited to events with a potential inflammatory or immune-mediated mechanism and which may require more frequent monitoring and/or interventions such as steroids, immunosuppressants and/or hormone replacement therapy. An immune-related adverse event (irAE) is defined as an adverse event that is associated with drug exposure and is consistent with an immune-mediated mechanism of action and where there is no clear alternate aetiology. Serologic, immunologic, and histologic (biopsy) data, as appropriate, should be used to support an irAE diagnosis. Appropriate efforts should be made to rule out neoplastic, infectious, metabolic, toxin, or other etiologic causes of the irAE.

If the Investigator has any questions in regards to an adverse event (AE) being an irAE, the Investigator should promptly contact the Principal Investigator.

Specific to this study, there are no AESI expected for the combination.

8. CORRELATIVE STUDIES

If the Day 15 treatment dosing is delayed, patients should undergo blood and tumor collection for Day 15 correlative studies, as scheduled and described in section 5.3.5.6, and outlined in Table 7.

Omission of correlative study sample collection(s) must be discussed and approved on a case-by-case basis by the Principal Investigator.

8.1 Pharmacokinetic assessments

Sitravatinib plasma levels as monotherapy and in combination with Nivolumab will be collected from all enrolled patients. Please refer to Table 7 for timelines and windows for sample collection.

Residual blood samples used for Sitravatinib plasma levels may also be used for exploratory pharmacokinetics (PK) and pharmacodynamics (PD) analyses related to Sitravatinib treatment alone or in combination with Nivolumab. This could include using leftover blood samples for exploratory, alternative PK assay development and analysis.

8.1.1 Sample handling, labeling, and shipping instructions

Approximately 50 mL of blood will be collected at each time point. Please refer to the SNOW-001 Laboratory Manual for detailed instructions about collection, handling and shipment of samples.

The actual collection date and time of each sample will be entered on the Pharmacokinetics Blood Collection eCRF pages.

8.2 Biomarker assessments

Biomarker analyses will be used to investigate the pharmacodynamic effects of treatment with single agent Sitravatinib and its combination with Nivolumab to determine how changes in the markers may relate to investigational therapy exposures. This is a proof-of-concept study to investigate the validity of preclinical findings in human subjects.

Please refer to Table 7 for timelines of biomarker sample collection.

Priorities for biomarker assessment are described in, but not restricted to, Tables 9 and 10.

Residual tissue samples used for biomarker analysis may also be used for exploratory biomarker analyses related to Sitravatinib treatment alone or in combination with Nivolumab. This could include using leftover tissue for exploratory, fluorescence-activated cell sorting, and alternative pharmacodynamic assay development and analysis.

The sample collection information must be entered on the appropriate Biomarker eCRF page(s) and requisition form(s). Detailed instructions for the collection, handling, and shipment of tumor samples are outlined in the SNOW-001 laboratory manual.

^{18}FAZA PET testing is optional, however is strongly encouraged to be performed within screening and pre-surgery assessment periods.

	Days of sample collection	Sample	Processing
**Tumor Based**			
**<u>Priority 1:</u>**<br>PD-L1 IHC and immunoprofiling	• Pre-treatment: Screening (baseline day -10 to -1)<br>• Post-Sitravatinib (on the day of on-treatment biopsy)<br>• Surgical specimen post-Sitravatinib and post-Nivolumab	1st and 4th core of tumor tissue	Obtained tumor cores will be processed according to the SNOW-001 laboratory manual.
**<u>Priority 2:</u>**<br>Flow Cytometry<br>*Including T-cell subsets, NK cells and myeloid-derived cell subsets.*	• Pre-treatment: Screening (baseline day -10 to -1)<br>• Post-Sitravatinib (on the day of on-treatment biopsy)<br>• Surgical specimen post-Sitravatinib and post-Nivolumab	2nd, 3rd, 5th and subsequent cores of tumor tissue will be pooled and distributed for Priorities 2, 3 and 4.<br><br>Priority 3 will be only performed on the Surgical specimen. Baseline and Post-Sitravatinib samples will be initially banked and DNA/RNA sequencing and/or alternate transcriptomic assays will be performed at a later date if appropriate.	
**<u>Priority 3:</u>**<br>DNA/RNA Sequencing	• Pre-treatment: Screening (baseline day -10 to -1)<br>• Post-Sitravatinib (on the day of on-treatment biopsy)<br>• Surgical specimen post-Sitravatinib and post-Nivolumab		
**<u>Priority 4:</u>**<br><u>FACS sorting</u><br>*Including bulk and single-cell RNA-seq for immune and tumor cell populations*	• Pre-treatment: Screening (baseline day -10 to -1)<br>• Post-Sitravatinib (on the day of on-treatment biopsy)<br>• Surgical specimen post-Sitravatinib and post-Nivolumab		

Table 9. Tumor-based biomarker collection

	Days of sample collection	Sample	Processing
**Blood Based**			
**Immune Assessment:**<br>Flow cytometry<br>*Including circulating tumor DNA (ctDNA), T-cell subsets, NK cells and myeloid-derived cell subsets.*	• Pre-treatment: Screening (baseline day -10 to -1)<br>• Post-Sitravatinib (day 15)<br>• Surgical specimen post- Sitravatinib and post-Nivolumab	Three 10-ml lavender-top EDTA tubes at each time point.	Obtained blood samples will be processed according to the SNOW-001 laboratory manual.
**Immune Assessment:**<br>Serum-based cytokines and HPLC<br>*Including PIP panel (IFN-g, IL-1b, IL-2, IL-4, IL-6, IL-8, IL-10, IL-12p70, TNF-a), CD8A, CXCL9-11, TBX21, GZMB, HPLC includes IDO and arginase*	• Pre-treatment: Screening (baseline day -10 to -1)<br>• Post-Sitravatinib (day 15)<br>• Surgical specimen post-Sitravatinib and post-Nivolumab	Two 4-ml SST tubes	
**Normal DNA Sequence**	Pre-treatment: Screening (baseline day -10 and -1)	One 10-mL whole blood once only (lavender tube – EDTA)	
**Sitravatinib Plasma levels**	• At baseline<br>• On the day of on-treatment biopsy<br>• On the day of surgery	One 6-mL green top (sodium heparin) at each time point.	

Table 10. Blood-based biomarker collection

8.3 Preliminary activity assessments

All patients enrolled in the study are to be evaluated for disease activity as described in Table 7. Tumor assessment at baseline should include CT or MRI of the head and neck. No further imaging will be performed before surgery unless clinical indicated due to suspected disease progression. Disease progression will be determined as per RECIST v1.1.

Preliminary activity will be evaluated by examination and photographic assessments.

Potential histopathological changes observed in the resected primary tumor and lymph nodes as well as potential pathologic response will be evaluated including, but not limited to, rate of downstaging, rate of positive margins, rate of extracapsular extension and Ki67 proliferative index activity.

8.4 ^{18}FAZA PET assessments

^{18}FAZA PET imaging will be an optional component of the study. If the patient consents to assessment by ^{18}FAZA PET, imaging studies will be performed as described in Table 7. Each imaging session will take place over approximately 2.5 hours, and includes an initial dynamic scan to completely characterize the initial rapid phase of ^{18}FAZA uptake, followed by a later static scan.

^{18}F-FAZA 5.2 MBq/kg (minimum 250 MBq, maximum 600 MBq) will be administered to patients with each PET scan. The ^{18}F-FAZA effective radiation dose equivalent is estimated to be 0.0165 mSv/MBq[76]which translates to between 1.6 and 10 mSv for the range of activities to be used for each of 2 scans in this study. A previous clinical biodistribution study of ^{123}I-IAZA in normal, healthy volunteers showed the bladder to receive the highest radiation dose (0.2 mGy/mBq) because of renal clearance and accumulation of the tracer[77]. Applying this biodistribution model to ^{18}F-FAZA, the patients in this study will receive a bladder dose of 50-120 mGy for each scan, or 100-240 mGy total for 2 scans.

Details on ^{18}FAZA PET procedures and image analysis are described in the SNOW-001 laboratory manual.

9. ETHICAL AND REGULATORY REQUIREMENTS

9.1 Ethical conduct of the study

The Principal Investigator will ensure that this study is conducted in agreement with the Declaration of Helsinki. The protocol has been written, and the study will be conducted according to the ICH Harmonized Tripartite Guideline for Good Clinical Practice.

The protocol will be approved by the Local, Regional or National Ethics Committees.

9.2 REB composition

The composition and procedures of the REB will be compliant with the ICH-Good Clinical Practice Guidelines and be consistent with Canadian regulatory requirements.

9.3 Initial approval

Documentation of full board approval of the initial protocol and the consent form must be received prior to local activation.

9.4 Annual re-approvals

Annual re-approval is required for as long as the trial is open to patient accrual or patients are receiving protocol treatment or undergoing protocol-mandated interventions.

9.5 Amendments / Revisions

All amendments or revisions to the protocol must undergo review by local REB and Mirati prior to implementation. If full board approval of an amendment is required it will be specified.

Amendments will be reviewed and approved by Health Canada (if applicable) and institutional REB prior to implementation, EXCEPT when the amendment eliminates an immediate hazard to clinical trial subjects.

9.6 Informed consent document

The REB of an institution must approve the consent form document, which will be used at that center prior to its activation; changes to the consent form in the course of the study will also require REB notification/approval.

It is essential that the consent form contain a clear statement that gives permission for 1) information to be sent to and 2) source medical records to be reviewed by TIP and other agencies as necessary. In addition, the consent form should include all elements required by ICH-Good Clinical Practice Guidelines.

Patients who cannot give informed consent (i.e. mentally incompetent patients, or those physically incapacitated such as comatose patients) are not to be recruited into the study. Patients competent but physically unable to sign the consent form may have the document signed by their nearest relative or legal guardian. Each patient will be provided with a full explanation of the study before consent is requested.

9.7 Serious adverse events, safety updates, and Investigator brochure updates

During the course of the study, serious adverse events, safety updates or Investigator brochure updates may be sent to the site for reporting to the REB.

9.8 Warnings and precautions

No evidence available at the time of the approval of this study protocol indicated that special warnings or precautions were appropriate, other than those noted in the accompanying Investigator's Brochures (IBs). Additional safety information collected between IB updates will be communicated by Mirati to the Tumor Immunotherapy Program. This information will be included in the amended patient informed consent and should be discussed with the patient during the study as needed.

10. PUBLICATION POLICY

10.1 Authors

The first and last authors will generally be the Principal Investigator(s) of the study, or their appointed delegate(s). A limited number of the members of the institutions involved on the trial and representatives of Mirati may be credited as authors depending upon their level of involvement in the study. Additional authors will be those who have made a significant contribution to the overall success of the study. This contribution will be assessed, in part but not entirely, in terms of patients enrolled and will be reviewed at the end of the trial by the Principal Investigator. The Tumor Immunotherapy Program publication guidelines will be followed.

10.2 Responsibility for publication

It will be the responsibility of the study chair to write up the results of the study within a reasonable time of its completion. If after a period of six months following the analysis of study results the draft is not substantially complete, the central office reserves the right to make other arrangements to ensure timely publication. Although the study chair or central office have full discretion to publish some or all of the results of the study, this material will be submitted to Mirati for review in advance of submission for publication.

10.3 Submission of materials for presentation or publication

Material may not be submitted for presentation or publication without approval by the Principal Investigator and without prior review by Mirati. Supporting groups and agencies will be acknowledged.

11. DATA COLLECTION AND MANAGEMENT

11.1 Data confidentiality

Information about study subjects will be kept confidential and managed under the applicable local and institutional laws and regulations.

In the event that a subject revokes authorization to collect or use his/her health information, the Investigator, by regulation, retains the ability to use all information collected prior to the revocation of subject authorization. For subjects that have revoked authorization to collect or use his/her, attempts should be made to obtain permission to collect follow-up safety information (e.g. has the patient experienced any new or worsened AEs) at the end of their scheduled study period.

11.2 Source documents

Source documents provide evidence for the existence of the patient and substantiate the integrity of the data collected. Source documents are filed at the Investigator's site.

Data entered in the eCRFs that are transcribed from source documents must be consistent with the source documents or the discrepancies must be explained. The Investigator may need to request previous medical records or transfer records, depending on the study; also current medical records must be available.

For eCRFs all data must be derived from source documents.

11.3 Direct access to source data and documents

The Investigator / institution will permit study-related monitoring, audits, ethics committee review and regulatory inspection, providing direct access to all related source data / documents. eCRFs and all source documents, including progress notes and copies of laboratory and medical test results must be available at all times for review by the on-site monitor, auditor and inspection by health authorities (e.g. Health Canada). The Clinical Research Associate (CRA) / on site monitor and auditor may review all eCRFs, and written informed consents. The accuracy of the data will be verified by reviewing the available source documents.

11.4 Retention of patient records and study files

This study is conducted under a CTA with Health Canada, therefore ICH Good Clinical Practice guidelines apply. All essential documents should be retained until at least two years after the last approval of a marketing application in an ICH region and until there are no pending or contemplated marketing applications in ICH region or at least two years have elapsed since the formal discontinuation of clinical development of the investigational product or for 25 years, whichever is longer. These documents should be retained for a longer period however if required by the applicable regulatory requirements or by an agreement with the Tumor Immunotherapy Program. It is the responsibility of the Tumor Immunotherapy Program to inform the Investigator/institution as to when these documents no longer need to be retained. The Investigator/institution should take measures to prevent accidental or premature destruction of these documents.

TIP will notify all the trial Investigators/institutions and all the regulatory authorities if clinical development of an investigational product discontinues or when trial related records are no longer needed.

12. STUDY MANAGEMENT

12.1 Training of study site personnel

Protocol training will be completed and documented for all study site personnel listed on the delegation log. Documentation of training will be filed with the regulatory documents.

12.2 Monitoring of the study

This is an investigator-initiated study and study monitoring will be performed by the Tumor Immunotherapy Program Central Office. Data in the Medidata Rave eCRFs will be reviewed on a regular basis and quality assurance measures will be performed. Electronic data queries as well as paper query letters may be issued to the site.

13. DATA MANAGEMENT GUIDELINES

13.1 Case report form completion

At the time of patient registration, the paper Eligibility Checklist CRF must be completed using black or blue ink. Any errors must be crossed out so that the original entry is still visible, the correction clearly indicated and then initialed and dated by the individual making the correction.

This study will utilize electronic data capture using the Medidata Rave® platform. Site staff access to Medidata Rave will be initiated at the time of site activation. eCRFs will be completed using any internet-ready computer, and each assigned user will have a password-protected secure sign-in. Study-specific document forms may be developed directly from eCRFs for ease of data collection and can be considered as source. eCRF entries will be made on site at the participating centre.

13.2 Case report form submission schedule

eCRFs will be completed according to the schedule noted below. The Investigator must electronically sign and date a declaration on the electronic CRF attesting to his/her responsibility for the quality of all data entered and that the data represents a complete and accurate record of each patient's participation in the study.

Case Report Form	Completion Schedule
Eligibility Checklist	At the time of registration
Baseline eCRFs	Within 2 weeks of on-study date
On Treatment Visits	Within 2 weeks of visit date
Surgery Visit	Within 2 weeks of visit date
Safety Follow Up Visit	Within 2 weeks of patient visit/contact
Disease Progression Follow Up	Within 2 weeks of patient contact/chart review

Table 11. Case report form completion schedule

14. APPENDICES

14.1 Pre-existing autoimmune diseases and immune deficiencies

Subjects should be carefully questioned regarding their history of acquired or congenital immune deficiencies or autoimmune disease. Subjects with any history of immune deficiencies or autoimmune disease listed in the table below are excluded from participating in the study. Possible exceptions to this exclusion could be subjects with a medical history of such entities as atopic disease or childhood arthralgias where the clinical suspicion of autoimmune disease is low. Patients with a history of autoimmune-related hypothyroidism on a stable dose of thyroid replacement hormone may be eligible for this study. In addition, transient autoimmune manifestations of an acute infectious disease that resolved upon treatment of the infectious agent are not excluded (e.g., acute Lyme arthritis). Please contact the Principal Investigator regarding any uncertainty over autoimmune exclusions.

Acute disseminated encephalomyelitis Addison's disease<br>Ankylosing spondylitis<br>Antiphospholipid antibody syndrome<br>Aplastic anemia<br>Autoimmune hemolytic anemia<br>Autoimmune hepatitis<br>Autoimmune hypoparathyroidism<br>Autoimmune hypophysitis<br>Autoimmune myocarditis<br>Autoimmune oophoritis<br>Autoimmune orchitis<br>Autoimmune thrombocytopenic purpura<br>Behcet's disease<br>Bullous pemphigold<br>Chronic fatigue syndrome<br>Chronic inflammatory demyelinating polyneuropathy<br>Chung-Strauss syndrome<br>Crohn's disease	Dermatomyositis<br>Diabetes mellitus type 1<br>Dysautonomia<br>Epidermolysis bullosa acquista<br>Gestational pemphigoid<br>Giant cell arteritis<br>Goodpasture's syndrome<br>Graves' disease<br>Guillain-Barré syndrome<br>Hashimoto's disease<br>IgA nephropathy<br>Inflammatory bowel disease<br>Interstitial cystitis<br>Kawasaki's disease<br>Lambert-Eaton myasthenia syndrome<br>Lupus erythematosus<br>Lyme disease<br>Chronic Meniere's syndrome<br>Mooren's ulcer<br>Morphea<br>Multiple sclerosis<br>Myasthenia gravis	Neuromyotonia<br>Opsoclonus myoclonus syndrome<br>Optic neuritis<br>Ord's thyroiditis<br>Pemphigus<br>Pernicious anemia<br>Polyarteritis nodusa<br>Polyarthritis<br>Polyglandular autoimmune syndrome<br>Primary biliary cirrhosis<br>Psoriasis<br>Reiter's syndrome<br>Rheumatoid arthritis<br>Sarcoidosis<br>Scleroderma<br>Sjögren's syndrome<br>Stiff-Person syndrome<br>Takayasu's arteritis<br>Ulcerative colitis<br>Vogt-Kovanagi-Harada disease<br>Wegener's granulomatosis

Table 12. Autoimmune disease

14.2 Toxicity management guidelines

14.2.1 Immune-mediated toxicities

The following are guidelines for management of potential immune-related toxicities. Alternative approaches to the recommendations are allowed in discussion with the Principal Investigator.

Patients should be thoroughly evaluated to rule out any alternative etiology such as disease progression, concomitant medications, infections, etc. In the absence of a clear alternative etiology, all events should be considered potentially immune-related.

<table>
<tr><th colspan="2">Dose Modifications*</th><th>Toxicity Management</th></tr>
<tr><td colspan="2">Drug administration modifications of study drug/study regimen will be made to manage potential immune-related AEs based on severity of treatment-emergent toxicities graded per NCI CTCAE v5.0.
In addition to the criteria for permanent discontinuation of study drug/regimen based on CTC grade/severity (table below) , permanently discontinue study drug/study regimen for the following conditions:
• Inability to reduce corticosteroid to a dose of ≤10 mg of prednisone per day (or equivalent) within 12 weeks after last dose of study drug/regimen
• Recurrence of a previously experienced Grade 3 treatment-related AE following resumption of dosing.</td><td rowspan="3">It is recommended that management of irAEs follow the guidelines presented in this table
– Patients should be thoroughly evaluated to rule out any alternative etiology (e.g., disease progression, concomitant medications, infections, etc.)
– In the absence of a clear alternative etiology, all events should be considered potentially immune related.
– Symptomatic and topical therapy should be considered for low-grade (Grade 1 or 2, unless otherwise specified) events
– For persistent (greater than 3 to 5 days) low-grade (Grade 2) or severe (Grade ≥3) events promptly start prednisone PO 1-2mg/kg/day or IV equivalent
– If symptoms recur or worsen during corticosteroid tapering 28 days of taper), increase the corticosteroid dose (prednisone dose [e.g. up to 2-4mg/kg/day PO or IV equivalent]) until stabilization or improvement of symptoms, then resume corticosteroid tapering at a slower rate (> 28 days of taper)</td></tr>
<tr><td>Grade 1</td><td>No dose modifications.</td></tr>
<tr><td>Grade 2</td><td>Hold study drug/study regimen dose until Grade 2 resolution to ≤ Grade 1
• If toxicity worsens then treat as Grade 3 or Grade 4.
Study drug/study treatment can be resumed once event stabilizes to Grade ≤1 after completion of steroid taper.</td></tr>
</table>

	Patients with endocrinopathies who may require prolonged or continued steroid replacement can be retreated with study drug/study regimen on the following conditions: 1) the event stabilizes and is controlled, 2) the patient is clinically stable as per Investigator or treating physician's clinical judgment, and 3) doses of prednisone are at less than or equal to 10mg/day or equivalent.	– More potent immunosuppressives such as TNF inhibitors (e.g. infliximab) – (also refer to the individual sections of the immune related adverse event for specific type of immunosuppressive) should be considered for events not responding to systemic steroids.<br>– Discontinuation of study drug is not mandated for Grade 3 / Grade 4 inflammatory reactions attributed to local tumor response (e.g. inflammatory reaction at sites of metastatic disease, lymph nodes etc.). Continuation of study drug in this situation should be based upon a benefit/risk analysis for that patient
Grade 3	Depending on the individual toxicity, may permanently discontinue study drug/study regimen. Please refer to guidelines below.	
Grade 4	Permanently discontinue study drug/study regimen.<br><br>Note: For Grade 3 and above asymptomatic amylase or lipase levels hold study drug/regimen and if complete work up shows no evidence of pancreatitis, may continue or resume study drug/regimen	

Table 13. Overall management for immune-related adverse events.

*Dose modification guidelines apply to all study drugs that is considered at least possibly contributing to relevant immune-mediated toxicity.

Clinical Study Protocol
Protocol Number: SNOW / Version 04/ 01 Jul 2020

14.2.2 Management algorithms for Nivolumab immune-mediated toxicities

Renal Adverse Event Management Algorithm

Rule out non-inflammatory causes. If non-inflammatory cause, treat accordingly and continue I-O therapy

Grade of Creatinine Elevation (NCI CTCAE v4)	Management	Follow-up
**Grade 1** Creatinine > ULN and > than baseline but ≤ 1.5x baseline	• Continue I-O therapy per protocol<br>• Monitor creatinine weekly	<u>If returns to baseline</u>:<br>•Resume routine creatinine monitoring per protocol<br><u>If worsens:</u><br>•Treat as Grade 2 or 3/4
**Grade 2-3** Creatinine > 1.5x baseline to ≤ 6x ULN	• Delay I-O therapy per protocol<br>• Monitor creatinine every 2-3 days<br>• 0.5 to 1.0 mg/kg/day methylprednisolone IV or oral equivalent<br>• Consider renal biopsy with nephrology consult	<u>If returns to Grade 1:</u><br>•Taper steroids over at least 1 month, consider prophylactic antibiotics for opportunistic infections, and resume I-O therapy and routine creatinine monitoring per protocol<br><u>If elevations persist > 7 days or worsen:</u><br>•Treat as Grade 4
**Grade 4** Creatinine > 6x ULN	• Discontinue I-O therapy per protocol<br>• Monitor creatinine daily<br>• 1.0-2.0 mg/kg/day methylprednisolone IV or IV equivalent<br>• Consult nephrologist<br>• Consider renal biopsy	<u>If returns to Grade 1</u> : Taper steroids over at least 1 month and add prophylactic antibiotics for opportunistic infections

GI Adverse Event Management Algorithm

Rule out non-inflammatory causes. If non-inflammatory cause is identified, treat accordingly and continue I-O therapy. Opiates/narcotics may mask symptoms of perforation. Infliximab should not be used in cases of perforation or sepsis.

Grade of Diarrhea/ Colitis (NCI CTCAE v4)	Management	Follow-up
**Grade 1**<br>Diarrhea:< 4 stools/day over baseline;<br>Colitis: asymptomatic	• Continue I-O therapy per protocol<br>• Symptomatic treatment	• Close monitoring for worsening symptoms.<br>• Educate patient to report worsening immediately<br>If worsens:<br>• Treat as Grade 2 or 3/4
**Grade 2**<br>Diarrhea: 4-6 stools per day over baseline; IV fluids indicated <24 hrs; not interfering with ADL<br>Colitis: abdominal pain; blood in stool	• Delay I-O therapy per protocol<br>• Symptomatic treatment	If improves to grade 1:<br>• Resume I-O therapy per protocol<br>If persists > 5-7 days or recur:<br>• 0.5-1.0 mg/kg/day methylprednisolone or oral equivalent<br>• When symptoms improve to grade 1, taper steroids over at least 1 month, consider prophylactic antibiotics for opportunistic infections, and resume I-O therapy per protocol.<br>If worsens or persists > 3-5 days with oral steroids:<br>• Treat as grade 3/4
**Grade 3-4**<br>Diarrhea (G3): ≥7 stools per day over baseline; incontinence; IV fluids ≥24 hrs; interfering with ADL<br>Colitis (G3): severe abdominal pain, medical intervention indicated, peritoneal signs<br>G4: life-threatening, perforation	• Discontinue I-O therapy per protocol<br>• 1.0 to 2.0 mg/kg/day methylprednisolone IV or IV equivalent<br>• Add prophylactic antibiotics for opportunistic infections<br>• Consider lower endoscopy	If improves:<br>• Continue steroids until grade 1, then taper over at least 1 month<br>If persists > 3-5 days, or recurs after improvement:<br>• Add infliximab 5 mg/kg (if no contraindication). Note: Infliximab should not be used in cases of perforation or sepsis

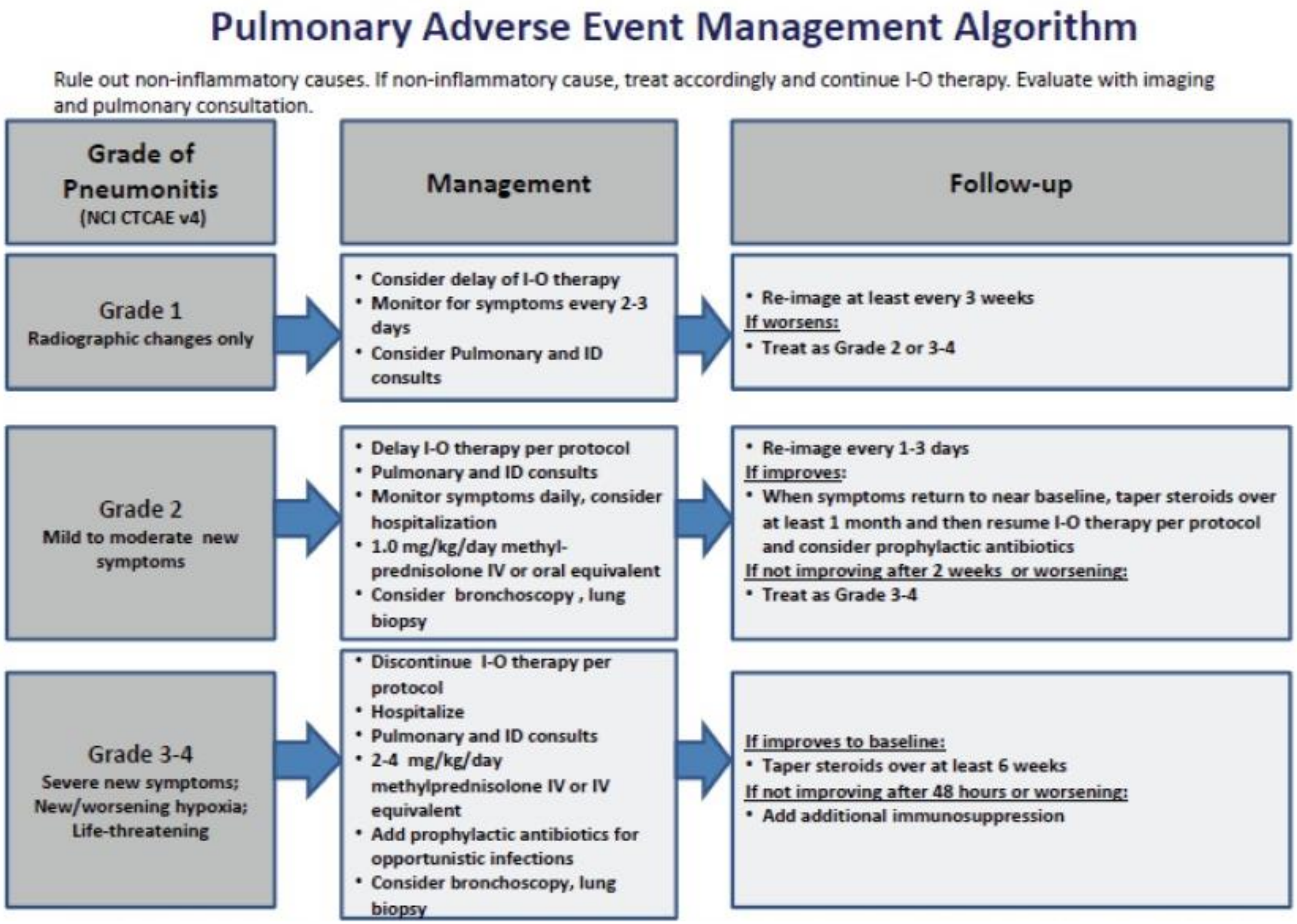
Pulmonary Adverse Event Management Algorithm
Rule out non-inflammatory causes. If non-inflammatory cause, treat accordingly and continue I-O therapy. Evaluate with imaging and pulmonary consultation.
Grade of Pneumonitis (NCI CTCAE v4)
Management
Follow-up
Grade 1
Radiographic changes only
• Consider delay of I-O therapy
• Monitor for symptoms every 2-3 days
• Consider Pulmonary and ID consults
• Re-image at least every 3 weeks
If worsens:
• Treat as Grade 2 or 3-4
Grade 2
Mild to moderate new symptoms
• Delay I-O therapy per protocol
• Pulmonary and ID consults
• Monitor symptoms daily, consider hospitalization
• 1.0 mg/kg/day methyl-prednisolone IV or oral equivalent
• Consider bronchoscopy , lung biopsy
• Re-image every 1-3 days
If improves:
• When symptoms return to near baseline, taper steroids over at least 1 month and then resume I-O therapy per protocol and consider prophylactic antibiotics
If not improving after 2 weeks or worsening:
• Treat as Grade 3-4
Grade 3-4
Severe new symptoms; New/worsening hypoxia; Life-threatening
• Discontinue I-O therapy per protocol
• Hospitalize
• Pulmonary and ID consults
• 2-4 mg/kg/day methylprednisolone IV or IV equivalent
• Add prophylactic antibiotics for opportunistic infections
• Consider bronchoscopy, lung biopsy
If improves to baseline:
• Taper steroids over at least 6 weeks
If not improving after 48 hours or worsening:
• Add additional immunosuppression

Hepatic Adverse Event Management Algorithm

Rule out non-inflammatory causes. If non-inflammatory cause, treat accordingly and continue I-O therapy. Consider imaging for obstruction.

Grade of Liver Test Elevation (NCI CTCAE v4)	Management	Follow-up
**Grade 1**<br>AST or ALT > ULN to 3.0 x ULN <u>and/or</u> T. bili > ULN to 1.5 x ULN	• Continue I-O therapy per protocol	• Continue LFT monitoring per protocol<br><u>If worsens:</u><br>• Treat as Grade 2 or 3-4
**Grade 2**<br>AST or ALT > 3.0 to ≤ 5 x ULN <u>and/or</u> T. bili > 1.5 to ≤ 3 x ULN	• Delay I-O therapy per protocol<br>• Increase frequency of monitoring to every 3 days	<u>If returns to baseline</u>:<br>•Resume routine monitoring, resume I-O therapy per protocol<br><br><u>If elevations persist > 5-7 days or worsen :</u><br>• 0.5-1 mg/kg/day methylprednisolone or oral equivalent and when LFT returns to grade 1 or baseline, taper steroids over at least 1 month, consider prophylactic antibiotics for opportunistic infections, and resume I-O therapy per protocol
**Grade 3-4**<br>AST or ALT > 5 x ULN <u>or</u> T.bili >3 x ULN	• Discontinue I-O therapy*<br>• Increase frequency of monitoring to every 1-2 days<br>• 1.0 to 2.0 mg/kg/day methylprednisolone IV or IV equivalent**<br>• Add prophylactic antibiotics for opportunistic infections<br>• Consult gastroenterologist	<u>If returns to grade 2</u>:<br>• Taper steroids over at least 1 month<br><br><u>If does not improve in >3-5 days, worsens or rebounds:</u><br>• Add mycophenolate mofetil 1 g BID<br>• If no response within an additional 3-5 days, consider other immunosuppressants per local guidelines

Endocrinopathy Management Algorithm

Rule out non-inflammatory causes. If non-inflammatory cause, treat accordingly and continue I-O therapy. Consider visual field testing, endocrinology consultation, and imaging.

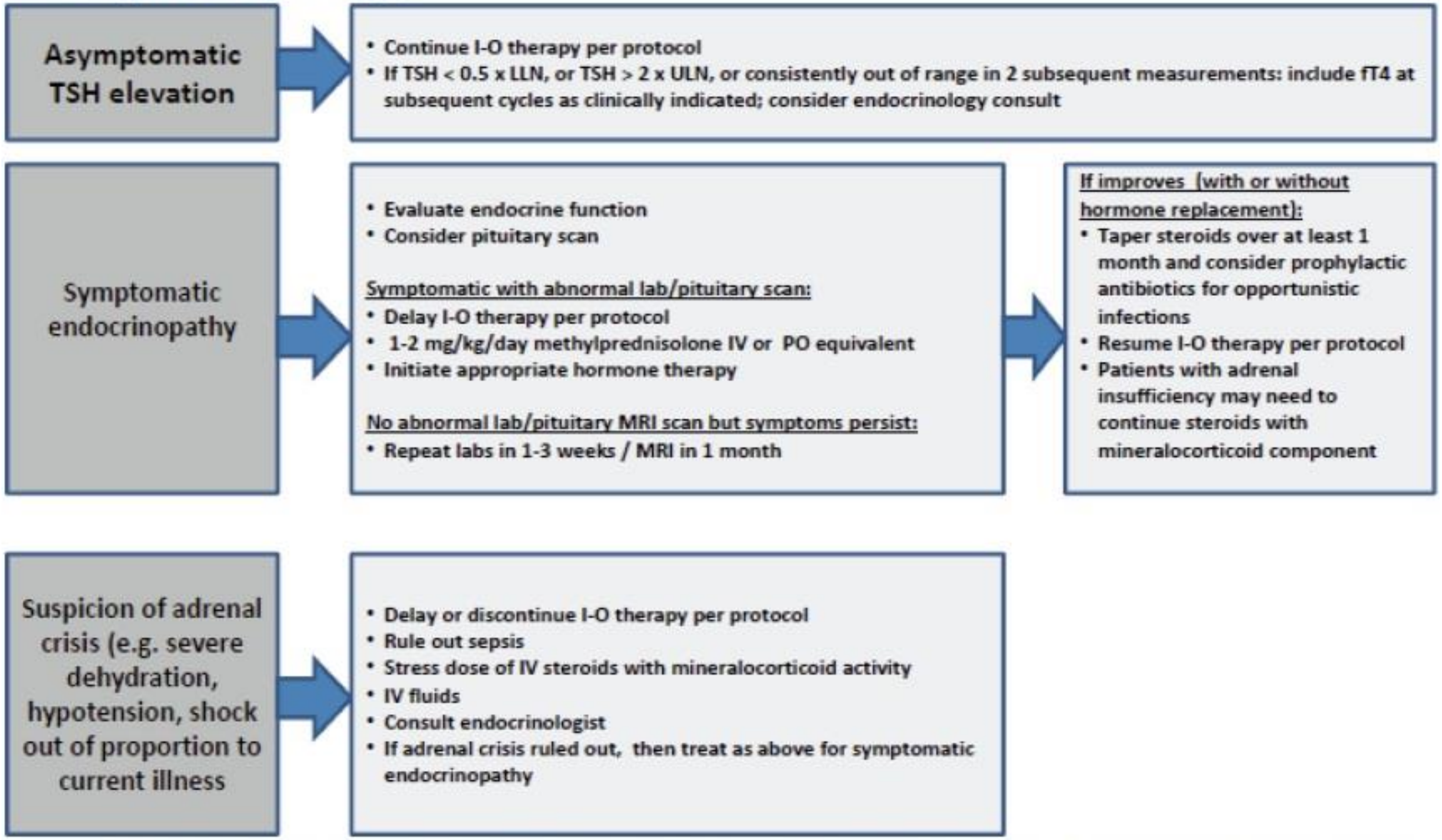

Clinical Study Protocol
Protocol Number: SNOW / Version 04/ 01 Jul 2020

Skin Adverse Event Management Algorithm

Rule out non-inflammatory causes. If non-inflammatory cause, treat accordingly and continue I-O therapy.

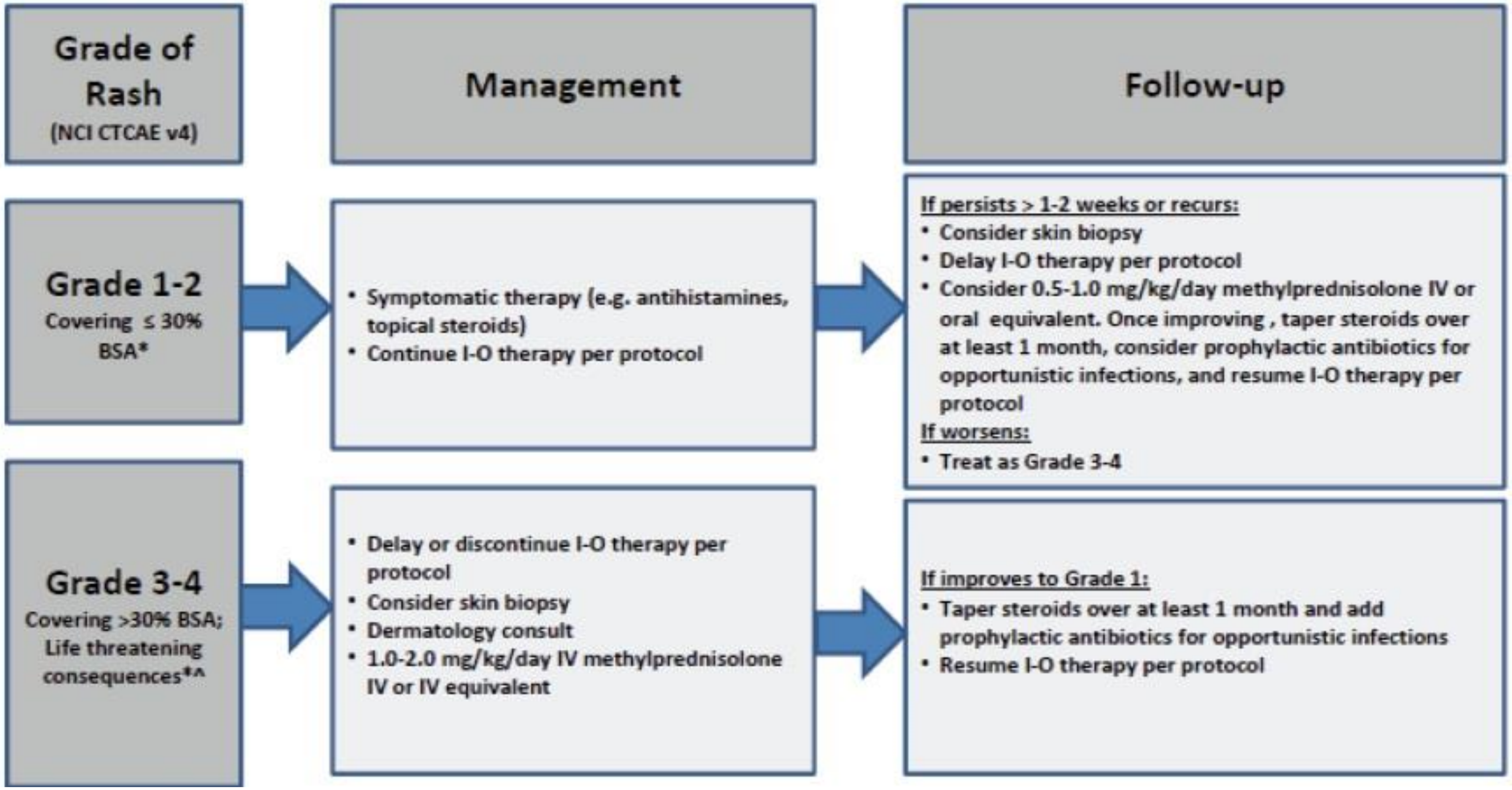

Page 84

Neurological Adverse Event Management Algorithm

Rule out non-inflammatory causes. If non-inflammatory cause, treat accordingly and continue I-O therapy.

Grade of Neurological Toxicity (NCI CTCAE v4)	Management	Follow-up
**Grade 1**<br>Asymptomatic or mild symptoms; Intervention not indicated	• Continue I-O therapy per protocol	Continue to monitor the patient.<br><u>If worsens:</u><br>• Treat as Grade 2 or 3-4
**Grade 2**<br>Moderate symptoms; Limiting instrumental ADL	• Delay I-O therapy per protocol<br>• Treat symptoms per local guidelines<br>• Consider 0.5 to 1.0 mg/kg/day methylprednisolone IV or PO equivalent	<u>If improves to baseline</u>:<br>•Resume I-O therapy per protocol when improved to baseline<br><u>If worsens</u>:<br>• Treat as Grade 3-4
**Grade 3-4**<br>Severe symptoms; Limiting self-care ADL; Life-threatening	• Discontinue I-O therapy per protocol<br>• Obtain neurology consult<br>• Treat symptoms per local guidelines<br>• 1.0-2.0 mg/kg/day IV methylprednisolone IV or IV equivalent<br>• Add prophylactic antibiotics for opportunistic infections	<u>If improves to Grade 2</u>:<br>• Taper steroids over at least 1 month<br><br><u>If worsens or atypical presentation:</u><br>• Consider IVIG or other immunosuppressive therapies per local guidelines

14.2.3 Infusion-related reactions

The following are guidelines for management of infusion-related reactions. Alternative approaches to the recommendations are allowed in discussion with the Principal Investigator.

CTC Grade/Severity	Dose Modifications	Toxicity Management
General guidance		Management per institutional standard at the discretion of investigator<br>Monitor patients for signs and symptoms of infusion-related reactions (e.g., fever and/or shaking chills, flushing and/or itching, alterations in heart rate and blood pressure, dyspnea or chest discomfort, skin rashes etc.) and anaphylaxis (e.g., generalized urticaria, angioedema, wheezing, hypotension, tachycardia, etc.)
Grade 1	The infusion rate of study drug/study regimen may be decreased by 50% or temporarily interrupted until resolution of the event	**For Grade 1 or Grade 2:**<br>– Acetaminophen and/or antihistamines may be administered per institutional standard at the discretion of the investigator.<br>– Consider premedication per institutional standard prior to subsequent doses.<br>– Steroids should not be used for routine premedication of ≤ Grade 2 infusion reactions.
Grade 2	The infusion rate of study drug/study regimen may be decreased 50% or temporarily interrupted until resolution of the event<br>Subsequent infusions may be given at 50% of the initial infusion rate	
Grade 3/4	Permanently discontinue study drug/study regimen	**For Grade 3 or 4:**<br>Manage severe infusion-related reactions per institutional standards (e.g., IM epinephrine, followed by IV diphenhydramine and ranitidine, and IV glucocorticoid).

Table 14. Infusion-related reactions management guidelines.

14.2.4 Non-immune mediated toxicities

The following are guidelines for management of non-immune mediated toxicities. Alternative approaches to the recommendations are allowed in discussion with the Principal Investigator.

Patients should be thoroughly evaluated to rule out any alternative etiology such as disease progression, concomitant medications, infections, etc. In the absence of a clear alternative etiology, all events should be considered potentially study drug-related.

CTC Grade/Severity	Dose Modification	Toxicity Management
**Any Grade**	Note: dose modifications are not required for adverse	Treat accordingly as per

CTC Grade/Severity	Dose Modification	Toxicity Management
	events not deemed to be related to study treatment (i.e. events due to underlying disease) or for laboratory abnormalities not deemed to be clinically significant.	institutional standard
**1**	No dose adjustment.	Treat accordingly as per institutional standard
**2**	Hold study drug/study regimen until resolution to ≤ Grade 1 or baseline.	Treat accordingly as per institutional standard
**3**	Hold study drug/study regimen until resolution to ≤ Grade 1 or baseline.<br><br>Following resolution of the toxicity to grade 1 or to the patient's baseline value, the patient may resume study treatment at Sitravatinib dose level lower. Resumption of therapy before the toxicity has resolved to grade 1, or to resume without a dose reduction, is permitted in discussion with the Principal Investigator.<br>An omitted dose of Sitravatinib will not be replaced, and the next dose should continue as per the initial schedule without date adjustments for the missed dose, with the exception of day 8 dosing, which is to be managed as described in section 5.3.5.6.<br><br>Delay of surgery beyond the initial 30-day window calculated from the start of study treatment is not allowed.	Treat accordingly as per institutional standard
**4**	Discontinue Study drug/study regimen (Note for Grade 4 labs, decision to discontinue would be based on accompanying clinical signs/symptoms and as per Investigator's clinical judgment and in consultation with the Principal Investigator.	Treat accordingly as per institutional standard

Table 15. Non-immune mediated toxicities management guidelines.

14.2.5 Sitravatinib (MGCD516) adverse events of interest

Based on review of the adverse events reported with Sitravatinib in context of the mechanism of action and nonclinical data, frequency, investigator assessment of causality, and medical literature, the adverse events listed in are considered to be adverse drug reactions (ADRs, i.e. adverse events with at least a reasonable possibility of a causal relationship to investigational product) for single-agent Sitravatinib. This data has been extracted from 2017 Sitravatinib Investigator Brochure.

MedDRA System Organ Class	MedDRA Preferred Term	Frequency of Treatment Emergent Adverse Events (All Grades) N= 86	Frequency of Treatment Emergent Adverse Events (Grade 3/4) N=86
**General Disorders and Administration Site Conditions**	Fatigue	46 (53.5%)	8 (9.3%)
	Asthenia	10 (11.6%)	0 (0.0%)
	Mucosal Inflammation[1]	6 (7.0%)	2 (2.3%)
**Gastrointestinal Disorders**	Diarrhea	41 (47.7%)	10 (11.6%)
	Nausea	32 (37.2%)	2 (2.3%)

	Vomiting	29 (33.7%)	3 (3.5%)
	Stomatitis[1]	8 (9.3%)	0 (0.0%)
**Vascular Disorders**	Hypertension	36 (41.9%)	20 (23.3%)
**Metabolism and Nutritional Disorders**	Decreased Appetite	31 (36.0%)	1 (1.2 %)
**Endocrine Disorders**	Thyroid Disorder[2]	20 (23.3%)	0 (0.0%)
**Skin and Subcutaneous Tissue Disorders**	Palmar-Plantar Erythrodysaesthesia Syndrome	12 (14.0%)	2 (2.3%)
**Investigations**	Ejection Fraction Decreased[3]	6 (7.0%)	4 (4.7%)

Table 16. Adverse Drug Reactions Associated with Sitravatinib (MGCD516)[4]

1. Mucositis was collectively assessed with MedDRA PTs Mucosal Inflammation and Stomatitis.
2. Thyroid Disorder was collectively assessed with MedDRAPTs Hypothyroidism (n=16), Hyperthyroidism (n=1), and Blood Thyroid Stimulating Hormone Increased (n=3)
3. Ejection Fraction Decreased < 50% includes events reported as adverse events (n=4) and events identified during review of Study 516-001 MUGA/Echocardiogram results (n=2)
4. Sources: Study 516-001 (2017 Sitravatinib IB data cut, Table 5.2).

14.3 Additional Safety Guidance

14.3.1 Assessment of Severity

Assessment of severity is one of the responsibilities of the investigator in the evaluation of AEs and SAEs. Severity will be graded according to the NCI CTCAE v5.0 as provided below. The determination of severity for all other events not listed in the NCI CTCAE v5.0 should be made by the investigator based upon medical judgment and the severity categories of Grade 1 to 5 as defined below.

Grade 1 (mild)	Asymptomatic or mild symptoms; clinical or diagnostic observations only; intervention not indicated.
Grade 2 (moderate)	Moderate; minimal, local or noninvasive intervention indicated; limiting age-appropriate instrumental ADL[1].
Grade 3 (severe)	Severe or medically significant but not immediately life-threatening; hospitalization or prolongation of hospitalization indicated; disabling; limiting self-care ADL[2]
Grade 4 (life threatening)	Life-threatening consequences; urgent intervention indicated.
Grade 5 (fatal)	Death (loss of life) as a result of an event.

Table 17. NCI CTCAE version 5.0

1. Instrumental ADL refer to preparing meals, shopping for groceries or clothes, using the telephone, managing money, etc. 2. Self-care ADL refer to bathing, dressing and undressing, feeding self, using the toilet, taking medications, and not bedridden.

It is important to distinguish between serious criteria and severity of an AE. Severity is a measure of intensity whereas seriousness is defined by the criteria outlined in Section 7.2.1. A Grade 3 AE need not necessarily be considered an SAE. For example, a Grade 3

headache that persists for several hours may not meet the regulatory definition of an SAE and would be considered a non-serious event, whereas a Grade 2 seizure resulting in a hospital admission would be considered an SAE.

The investigator is required to provide an assessment of relationship of AEs and SAEs to the investigational product.

An event will be considered "not related" to use of the investigational product if any of the following tests outlined in sections 14.4.1.1 and 14.4.1.2 are met.

14.3.1.1 Assessment of relationship

An unreasonable temporal relationship between administration of the investigational product and the onset of the event (eg, the event occurred either before, or too long after, administration of the investigational product for it to be considered product-related).

A causal relationship between the investigational product and the event is biologically implausible (eg, death as a passenger in an automobile accident).

A clearly more likely alternative explanation for the event is present (eg, typical adverse reaction to a concomitant drug and/or typical disease-related event).

Individual AE/SAE reports will be considered "related" to use of the investigational product if the "not related" criteria are not met.

"Related" implies that the event is considered to be "associated with the use of the drug" meaning that there is "a reasonable possibility" that the event may have been caused by the product under investigation (ie, there are facts, evidence, or arguments to suggest possible causation).

14.3.1.2 Relationship to Protocol Procedures

The investigator is also required to provide an assessment of relationship of SAEs to protocol procedures on the SAE Report Form. This includes nontreatment-emergent SAEs (ie, SAEs that occur prior to the administration of investigational product) as well as treatment-emergent SAEs. A protocol-related SAE may occur as a result of a procedure or intervention required during the study (eg, blood collection, washout of an existing medication).

The following guidelines should be used by investigators to assess the relationship of SAEs to the protocol:

Protocol related:	The event occurred due to a procedure/intervention that was described in the protocol for which there is no alternative etiology present in the subject's medical record.
Not protocol related:	The event is related to an etiology other than the procedure/ intervention that was described in the protocol (the alternative etiology must be

	documented in the study subject's medical record).

Table 18. Event relationship to protocol procedure.

14.4 Medications or substances to be avoided or used with caution during study treatment

14.4.1 Drugs that may prolong QTc interval

Bold font indicates medications or substances that might be relatively commonly used.

Italic font indicates medications for indications that are exclusionary for the current study or would likely result in discontinuation from study treatment with Sitravatinib for management of a concurrent illness. Please see https://crediblemeds.org/index.php. for most up to date information.

14.4.1.1 Drugs to be avoided (Known Risk of Torsades de Pointes)

Amiodarone, anagrelide, *arsenic trioxide*, astemizole (off US market), **azithromycin**, bepridil (off US market), chloroquine, **chlorpromazine**, cilostazol, **ciprofloxacin**, cisapride (off US market), **citalopram**, **clarithromycin**, cocaine, disopyramide, dofetilide, domperidone (not on US market), donepezil, dronedarone, **droperidol**, **erythromycin**, **escitalopram**, flecainide, fluconazole, gatifloxacin (off US market), grepafloxacin (not on US market), halofantrine (not on US market), haloperidol, ibogaine (not on US market), ibutilide, levofloxacin, levomepromazine / methotrimeprazine (not on US market), levomethadyl (off US market), levosulpiride (not on US market), mesoridazine (off US market), **methadone**, moxifloxacin, **ondansetron**, *oxaliplatin*, *pentamidine*, pimozide, probucol (off US market), procainamide, propofol, quinidine, roxithromycin (not on US market), sevoflurane, sotalol, sparfloxacin (off US market), sulpiride (not on US market), sultopride (non on US market), terfenadine (off US market), terlipressin (not on US market), terodiline (not on US market), thioridazine, *vandetanib*.

14.4.1.2 Drugs to be used with caution (Conditional Risk of Torsades de Pointes)

Amantadine, amisulpride (not on US market), amitriptyline, amphotericin B, *atazanavir*, bendroflumethiazide / bendrofluazide (not on US market), chloral hydrate, **diphenhydramine**, doxepin, **esomeprazole**, **famotidine**, **fluoxetine**, fluvoxamine, **furosemide / frusemide**, galantamine, garenoxacin (not on US market), **hydrochlorothiazide**, hydroxychloroquine, hydroxyzine, indapamide, itraconazole, ivabradine, ketoconazole, **lansoprazole**, **loperamide**, **metoclopramide**, metolazone, metronidazole, *nelfinavir*, olanzapine, **omeprazole**, **pantoprazole**, **paroxetine**, piperacillin/tazobactam, posaconazole, propafenone, quetiapine, quinine sulfate, ranolazine, **sertraline**, solifenacin, *telaprevir*, torsemide / torasemide, trazodone, voriconazole, ziprasidone.

14.4.2 Transporter substrates

Sensitive Substrates and Substrates with Narrow Therapeutic Index for P-gp and BCRP transporters

**Enzyme**	
P-gp	Aliskiren, ambrisentan, colchicine, dabigatran etexilate, **digoxin**, *everolimus*, **fexofenadine**, *imatinib*, *lapatinib*, *maraviroc*, *nilotinib*, posaconazole, ranolazine, saxagliptin, sirolimus, **sitagliptin**, talinolol, tolvaptan, *topotecan.*
BCRP	Methotrexate, *mitoxantrone*, *imatinib*, *irinotecan*, *lapatinib*, **rosuvastatin**, sulfasalazine, *topotecan.*

Sensitive Substrates and Substrates with Narrow Therapeutic Index for the indicated CYP Enzymes

**Enzyme**	
CYP2B6	Bupropion.
CYP2C8	Repaglinide.
CYP2D6	Atomoxetine, desipramine, **dextromethorphan**, *eliglustat,* nebivolol, **nortriptyline**, perphenazine, tolterodine, venlafaxine.
CYP3A	Alfentanil, avanafil, budesonide, buspirone, conivaptan, darifenacin, *darunavir*, *dasatinib*, dronedarone, ebastine, eletriptan, eplerenone, *everolimus*, **felodipine**, ibrutinib, *indinavir*, lomitapide, **lovastatin**, lurasidone, *maraviroc*, **midazolam**, naloxegol, nisoldipine, quetiapine, *saquinavir*, sildenafil, **simvastatin**, sirolimus, tacrolimus, ticagrelor, *tipranavir*, tolvaptan, triazolam, vardenafil.

The use of loperamide is allowed if needed as per investigator criteria.

15. LIST OF REFERENCES

1. Statistics CCSsACoC: Canadian Cancer Statistics 2015. Toronto, ON: Canadian Cancer Society, 2015.

2. Chinn SB, Myers JN: Oral Cavity Carcinoma: Current Management, Controversies, and Future Directions. J Clin Oncol 33:3269-76, 2015.

3. Blume-Jensen P., Hunter T: Oncogenic kinase signalling. Nature 411:355-65, 2001.

4. Christensen JG, Burrows J, et al: c-Met as a target for human cancer and characterization of inhibitors for therapeutic intervention. Cancer Lett; 225(1):1-26, 2005.

5. Gherardi E, Birchmeier W, et al: Targeting MET in cancer: rationale and progress. Nat Rev Cancer; 12(2):89-103, 2012.

6. Levi Arnold, Enders J, et al: Activated HGF-c-Met Axis in Head and Neck Cancer. Cancers; 9 (169) 2017.

7. Cortesina, G, Martone T, et al: Staging of head and neck squamous cell carcinoma using the MET oncogene product as marker of tumor cells in lymph node metastases. Int. J. Cance;, 89, 286–292, 2000.

8. Tao X, Hill K.S, et al: Silencing Met receptor tyrosine kinase signaling decreased oral tumor growth and increased survival of nude mice. Oral Oncol.; 50, 104–112, 2014.

9. Tepper S.R, Zuo Z, et al: Growth factor expression mediates resistance to EGFR inhibitors in head and neck squamous cell carcinomas. Oral Oncol: 56, 62–70, 2016.

10. Uehara M, Sano K, et al: Expression of vascular endothelial growth factor and prognosis of oral squamous cell carcinoma. Oral Oncol: **40**, 321-5, 2004.

11.Wilkie MD, Emmett MS, et al: Relative expression of vascular endothelial growth factor isoforms in squamous cell carcinoma of the head and neck. Head Neck, **38**, 775-81, 2016.

12. Brand T, Lida M, et al: AXL is a logical molecular target in head and neck squamous cell carcinoma. Clin Cancer Res; 21(11): 2601–2612, 2015.

13. Nakagawa T, Tohyama O, et al: E7050: A dual c-Met and VEGFR-2 tyrosine kinase inhibitor promotes tumor regression and prolongs survival in mouse xenograft models. Cancer Sci., 101, 210–215, 2010.

14. Kumar D, Kandl, C, et al : Mitigation of tumor-associated fibroblast-facilitated head and neck cancer progression with anti-hepatocyte growth factor antibody ficlatuzumab. JAMA Otolaryngol. Head Neck Surg. 141, 1133–1139, 2015.

15. Seiwert T, Sarantopoulos, J, et al: Phase II trial of single-agent foretinib (GSK1363089) in patients with recurrent or metastatic squamous cell carcinoma of the head and neck. Investig.; New Drugs, 31, 417–424, 2013.

16. Brands RC., Knierim LM., et al. Targeting VEGFR and FGFR in head and neck squamous cell carcinoma in vitro. Oncol Rep., 38(3):1877-1885. 2017

17. Lemke G, Rothlin CV: Immunobiology of the TAM receptors. Nat Rev Immunol.; 8(5):327-36. 2008

18. Lu Q, Lemke G: Homeostatic regulation of the immune system by receptor tyrosine kinases of the Tyro 3 family. Science; 293(5528):306-11. 2001.

19. Cook RS, Jacobsen KM, et al: MerTK inhibition in tumor leukocytes decreases tumor growth and metastasis. J Clin Invest; 123(8):3231-42. 2013.

20. Paolino M, Choidas A, et al: The E3 ligase Cbl-b and TAM receptors regulate cancer metastasis via

natural killer cells. Nature; 507(7493):508-12; 2014.

21. Chen PM, Liu KJ, et al: Induction of immunomodulatory monocytes by human mesenchymal stem cell-derived hepatocyte growth factor through ERK1/2. J Leukoc Biol; 96(2):295-303. 2014.

22. Pircher A, Wolf D, et al : Synergies of targeting tumor angiogenesis and immune checkpoints in non-small cell lung cancer and renal cell cancer: From basic concepts to clinical reality. Int J Mol Sci;18(11):1–15; 2017.

23. Terme M, Pernot S, et al : VEGFA-VEGFR pathway blockade inhibits tumor-induced regulatory T-cell proliferation in colorectal cancer. Cancer Res; 73(2):539-49; 2013.

24. Hendry S.A, Farnsworth R.H, et al: The role of the tumor vasculature in the host immune response: Implications for therapeutic strategies targeting the tumor microenvironment. Front. Immunol.; 7, 621, 2016.

25. Huang Y, Yuan J, et al: Vascular normalizing doses of antiangiogenic treatment reprogram the immunosuppressive tumor microenvironment and enhance immunotherapy. Proc. Natl. Acad. Sci. USA, 109, 17561–17566. 2012.

26. Shrimali R.K, Yu Z, et al: Antiangiogenic agents can increase lymphocyte infiltration into tumor and enhance the effectiveness of adoptive immunotherapyof cancer. Cancer Res;70, 6171–6180; 2010.

27. Apolo A, Tomita Y, et al: Effect of cabozantinib on immunosuppressive subsets in metastatic urothelial carcinoma. J Clin Oncol.; 32(Suppl 15): Abstract 4501; 2014.

28. Ozao-Choy J, Ma G, et al : The novel role of tyrosine kinase inhibitor in the reversal of immune suppression and modulation of tumor microenvironment for immune-based cancer therapies. Cancer Res.;69(6):2514-22; 2009.

29. Zou W, Chen L: Inhibitory B7-family molecules in the tumour microenvironment. Nat Rev Immunol 8:467-77, 2008.

30. Dong H, Strome SE, et al: Tumor-associated B7-H1 promotes T-cell apoptosis: a potential mechanism of immune evasion. Nat Med 8:793-800, 2002.

31. Cho YA, Yoon HJ, et al: Relationship between the expressions of PD-L1 and tumor-infiltrating lymphocytes in oral squamous cell carcinoma. Oral Oncol 47:1148-53, 2011.

32.Lyford-Pike S, Peng S, et al: Evidence for a role of the PD-1:PD-L1 pathway in immune resistance of HPV-associated head and neck squamous cell carcinoma. Cancer Res 73:1733-41, 2013.

33. Malm IJ, Bruno TC, et al: Expression profile and in vitro blockade of programmed death-1 in human papillomavirus-negative head and neck squamous cell carcinoma. Head Neck, 2014.

34. Biggar RJ, Chaturvedi AK, et al: AIDS-related cancer and severity of immunosuppression in persons with AIDS. J Natl Cancer Inst 99:962-72, 2007.

35. Long JL, Engels EA, et al: Incidence and outcomes of malignancy in the HAART era in an urban cohort of HIV-infected individuals. AIDS; 22:489-96, 2008.

36. Yilmaz T, Gedikoglu G, et al: Prognostic significance of Langerhans cell infiltration in cancer of the larynx. Otolaryngol Head Neck Surg 132:309-16, 2005.

37. Wei N, Tahan SR: S100+ cell response to squamous cell carcinoma of the lip: inverse correlation with metastasis. J Cutan Pathol 25:463-8, 1998.

38. Sakakura K, Chikamatsu K: Immune Suppression and Evasion in Patients with Head and Neck Cancer. Advances in Cellular and Molecular Otolaryngology 1: 21809, 2013.

39. Alexandrov LB, Nik-Zainal S, et al: Signatures of mutational processes in human cancer. Nature 500:415 -

21, 2013.

40. Cancer Genome Atlas N: Comprehensive genomic characterization of head and neck squamous cell carcinomas. Nature 517:576-82, 2015.

41. Segal NH, Antonia SJ, et al: Preliminary data from a multi-arm expansion study of MEDI4736, an anti-PD-L1 antibody. J Clin Oncol; 32, 2014.

42. Tumeh PC, Harview CL, et al: PD-1 blockade induces responses by inhibiting adaptive immune resistance. Nature 515:568-71, 2014.

43. Segal NH, Ou SI, et al: Safety and efficacy of MEDI4736, an anti-PD-L1 antibody, in patients from a squamous cell carcinoma of the head and neck (SCCHN) expansion cohort, 2015 ASCO Annual Meeting. Chicago, Il, J Clin Oncol, 2015, pp suppl; abstr 3011

44. Ferris RL, Gillison ML: Nivolumab for Recurrent Squamous-Cell Carcinoma of the Head and Neck. N Engl J Med, 2016.

45. Bahleda R., Braiteh F.S., et al : Long-Term Safety and Clinical Outcomes of Atezolizumab in Head and Neck Cancer: Phase Ia Trial Results. ESMO Annual Meeting. Annals of Oncology. 28 (suppl_5): v372-v394.

46. Zandberg D, Mesia R, et al. Durvalumab fo recurrent/metastatic (R/M) head and neck squamous cell carcinoma (HNSCC): preliminary results from a single-arm, phase 2 study. ESMO Annual Meeting. Annals of Oncology 28 (suppl_5): v372-v394. 2017.

47. Hoos A: Evolution of end points for cancer immunotherapy trials. Ann Oncol 23 Suppl 8:viii47-52, 2012.

48. Hanahan, D.; Weinberg, R.A. Hallmarks of cancer: The next generation. Cell, 144, 646–674.2010.

49. Fridman WH, Zitvogel L, et al: The immune contexture in cancer prognosis and treatment. Nat Rev Clin Oncol; 14(12):717–34. 2017.

50. De Ruiter EJ, Ooft ML, et al. The prognostic role of tumor infiltrating T-lymphocytes in squamous cell carcinoma of the head and neck: A systematic review and meta-analysis. Oncoimmunology;6(11):1–10. 2017

51. Zandberg DP, Strome SE. The role of the PD-L1:PD-1 pathway in squamous cell carcinoma of the head and neck. Oral Oncol;50(7):627–32. 2014.

52. Kansy BA, Concha-Benavente F, et al. PD-1 status in CD8+T cells associates with survival and anti-PD-1 therapeutic outcomes in head and neck cancer. Cancer Res.;77(22):6353–64. 2017.

53. Mattox A, Lee J, et al. PD-1 expression in head and neck squamous cell carcinomas derives primarily from functionally anergic CD4+ TILs in the presence of PD-L1+ TAMs. Cancer Res; 3453, 2017.

54. Hansen AR, Siu LL: PD-L1 Testing in Cancer: Challenges in Companion Diagnostic Development. JAMA Oncol:1-2, 2015.

55. Ferris R.L., Gonçalves A. et al: An Open-label, Multicohort, Phase 1/2 Study in Patients With Virus-Associated Cancers (CheckMate 358): Safety and Efficacy of Neoadjuvant Nivolumab in Squamous Cell Carcinoma of the Head and Neck. ESMO annual Meeting 2017. Annals of Oncology, 28 (suppl_5): v605-v649.

56. Chen L, Flies DB: Molecular mechanisms of T cell co-stimulation and co-inhibition. Nat Rev Immunol 13:227-42, 2013.

57. Gajewski TF, Schumacher T: Cancer immunotherapy. Curr Opin Immunol 25:259-60, 2013.

58. Gajewski TF, Woo SR, et al: Cancer immunotherapy strategies based on overcoming barriers within the

tumor microenvironment. Curr Opin Immunol 25:268-76, 2013.

59. Gajewski TF: Molecular profiling of melanoma and the evolution of patient-specific therapy. Semin Oncol 38:236-42, 2011.

60. Gajewski TF, Fuertes M, et al: Molecular profiling to identify relevant immune resistance mechanisms in the tumor microenvironment. Curr Opin Immunol 23:286-92, 2011.

61. Donia M, Lyngaa R, Hadrup SR: "Immunotherapy of cancer: present status and future promise": Danish Cancer Society Symposium, Copenhagen, Denmark, 23rd-25th September 2013. Cancer Immunol Immunother 63:985-9, 2014.

62. Isa AY, Ward TH, et al: Hypoxia in head and neck cancer. Br J Radiol 79:791-8, 2006.

63.Dos Santos M, Mercante AM, et al: HIF1-alpha expression predicts survival of patients with squamous cell carcinoma of the oral cavity. PLoS One 7:e45228, 2012.

64. Dunkel J, Vaittinen S, et al: Prognostic markers in stage I oral cavity squamous cell carcinoma. Laryngoscope 123:2435-41, 2013.

65. Eckert AW, Lautner MH, et al: Co-expression of Hif1alpha and CAIX is associated with poor prognosis in oral squamous cell carcinoma patients. J Oral Pathol Med 39:313-7, 2010.

66. Eckert AW, Lautner MH, et al: Coexpression of hypoxia-inducible factor-1alpha and glucose transporter-1 is associated with poor prognosis in oral squamous cell carcinoma patients. Histopathology 58:1136-47, 2011.

67. Klimowicz AC, Bose P, et al: The prognostic impact of a combined carbonic anhydrase IX and Ki67 signature in oral squamous cell carcinoma. Br J Cancer 109:1859-66, 2013.

68. Oliver RJ, Woodwards RT, et al: Prognostic value of facilitative glucose transporter Glut-1 in oral squamous cell carcinomas treated by surgical resection; results of EORTC Translational Research Fund studies. Eur J Cancer 40:503-7, 2004.

69. Perez-Sayans M, Suarez-Penaranda JM, et al: Expression of CA-IX is associated with advanced stage tumors and poor survival in oral squamous cell carcinoma patients. J Oral Pathol Med 41:667-74, 2012.

70. Noman MZ, Buart S, et al: The cooperative induction of hypoxia-inducible factor-1 alpha and STAT3 during hypoxia induced an impairment of tumor susceptibility to CTL-mediated cell lysis. J Immunol 182:3510-21, 2009.

71. Barsoum IB, Smallwood CA, et al: A mechanism of hypoxia-mediated escape from adaptive immunity in cancer cells. Cancer Res 74:665-74, 2014.

72. Messai Y, Gad S, et al: Renal Cell Carcinoma Programmed Death-ligand 1, a New Direct Target of Hypoxia-inducible Factor-2 Alpha, is Regulated by von Hippel-Lindau Gene Mutation Status. Eur Urol, 2015.

73. Noman MZ, Desantis G, et al: PD-L1 is a novel direct target of HIF-1alpha, and its blockade under hypoxia enhanced MDSC-mediated T cell activation. J Exp Med 211:781-90, 2014.

74. Sato J, Kitagawa Y, et al: 18F-fluoromisonidazole PET uptake is correlated with hypoxia-inducible factor-1alpha expression in oral squamous cell carcinoma. J Nucl Med 54:1060-5, 2013.

75. Halmos GB, Bruine de Bruin L, et al: Head and neck tumor hypoxia imaging by 18F-fluoroazomycin-arabinoside (18F-FAZA)-PET: a review. Clin Nucl Med 39:44-8, 2014.

76. Postema EJ, McEwan AJ, et al: Initial results of hypoxia imaging using 1-alpha-D: -(5-deoxy-5-[18F]-fluoroarabinofuranosyl)-2-nitroimidazole (18F-FAZA). Eur J Nucl Med Mol Imaging 36:1565-73, 2009.

77. Stypinski D, Wiebe LI., et al: Clinical pharmacokinetics of 123I-IAZA in healthy volunteers. Nucl Med

Commun 20:559-67, 1999.
